# Supplementary material for: Specialist healthcare services for concussion/mild traumatic brain injury in England: a consensus statement using modified Delphi methodology
Source: BMJ Open. 2023 Dec 9;13(12):e077022. doi: 10.1136/bmjopen-2023-077022 (PMC10729241; doi:10.1136/bmjopen-2023-077022)
Supplement: Supplementary data [file bmjopen-2023-077022supp001.pdf]

Supplementary material 1: Literature search strategy

The search was conducted in MEDLINE and Embase. Below is the search used for Embase and the equivalent search was done for MEDLINE.

Embase search strategy

1 brain concussion/

2 postconcussion syndrome/

3 "mild traumatic brain injur*".mp. [mp=title, abstract, heading word, drug trade name, original title, device
manufacturer, drug manufacturer, device trade name, keyword heading word, floating subheading word, candidate term word]

4 "concussion*".mp. [mp=title, abstract, heading word, drug trade name, original title, device manufacturer, drug
manufacturer, device trade name, keyword heading word, floating subheading word, candidate term word]

5 "concussive*".mp. [mp=title, abstract, heading word, drug trade name, original title, device manufacturer, drug
manufacturer, device trade name, keyword heading word, floating subheading word, candidate term word]

6 "mild head injur*".mp. [mp=title, abstract, heading word, drug trade name, original title, device manufacturer,
drug manufacturer, device trade name, keyword heading word, floating subheading word, candidate term word]

7 "mild brain injur*".mp. [mp=title, abstract, heading word, drug trade name, original title, device manufacturer,
drug manufacturer, device trade name, keyword heading word, floating subheading word, candidate term word]

8 "mtbi*".mp. [mp=title, abstract, heading word, drug trade name, original title, device manufacturer, drug
manufacturer, device trade name, keyword heading word, floating subheading word, candidate term word]

9 "mild TBI*".mp. [mp=title, abstract, heading word, drug trade name, original title, device manufacturer, drug

Karvandi E, *et al. BMJ Open* 2024; 13:e077022. doi: 10.1136/bmjopen-2023-077022

manufacturer, device trade name, keyword heading word, floating subheading word, candidate term word]

10 "commotio cerebri".mp. [mp=title, abstract, heading word, drug trade name, original title, device manufacturer,
drug manufacturer, device trade name, keyword heading word, floating subheading word, candidate term word]

11 "post-concussion symptom*".mp. [mp=title, abstract, heading word, drug trade name, original title, device
manufacturer, drug manufacturer, device trade name, keyword heading word, floating subheading word, candidate term word]

12 "post-concussion syndrome*".mp. [mp=title, abstract, heading word, drug trade name, original title, device
manufacturer, drug manufacturer, device trade name, keyword heading word, floating subheading word, candidate term word]

13 1 or 2 or 3 or 4 or 5 or 6 or 7 or 8 or 9 or 10 or 11 or 12

14 practice guideline/

15 clinical practice/

16 "guideline*".mp. [mp=title, abstract, heading word, drug trade name, original title, device manufacturer, drug
manufacturer, device trade name, keyword heading word, floating subheading word, candidate term word]

17 "clinical practic*".mp. [mp=title, abstract, heading word, drug trade name, original title, device manufacturer,
drug manufacturer, device trade name, keyword heading word, floating subheading word, candidate term word]

18 "recommend*".mp. [mp=title, abstract, heading word, drug trade name, original title, device manufacturer, drug
manufacturer, device trade name, keyword heading word, floating subheading word, candidate term word]

19 "best practic*".mp. [mp=title, abstract, heading word, drug trade name, original title, device manufacturer, drug
manufacturer, device trade name, keyword heading word, floating subheading word, candidate term word]

Karvandi E, *et al. BMJ Open* 2024; 13:e077022. doi: 10.1136/bmjopen-2023-077022

20 "clinical decision*".mp. [mp=title, abstract, heading word, drug trade name, original title, device manufacturer,
drug manufacturer, device trade name, keyword heading word, floating subheading word, candidate term word]
21 "clinical support*".mp. [mp=title, abstract, heading word, drug trade name, original title, device manufacturer,
drug manufacturer, device trade name, keyword heading word, floating subheading word, candidate term word]
22 "consensus".mp. [mp=title, abstract, heading word, drug trade name, original title, device manufacturer, drug
manufacturer, device trade name, keyword heading word, floating subheading word, candidate term word]
23 "position statement*".mp. [mp=title, abstract, heading word, drug trade name, original title, device
manufacturer, drug manufacturer, device trade name, keyword heading word, floating subheading word, candidate term word]
24 healthcare service.mp. or health service/
25 "*care system*".mp. [mp=title, abstract, heading word, drug trade name, original title, device manufacturer, drug
manufacturer, device trade name, keyword heading word, floating subheading word, candidate term word]
26 "service*".mp. [mp=title, abstract, heading word, drug trade name, original title, device manufacturer, drug
manufacturer, device trade name, keyword heading word, floating subheading word, candidate term word]
27 14 or 15 or 16 or 17 or 18 or 19 or 20 or 21 or 22 or 23 or 24 or 25 or 26 (3935187)
28 13 and 27

Supplementary material 2: Statements voting round 1

Supplementary material 2.1 Consensus Statement Voting Round 1

Statement	Agreement	Outcome
There should be a direct patient pathway from ED to specialist outpatient services available to	71%	None

all patients to ensure patients with persistent symptoms will be identified early.		
There is not sufficient evidence for injury-related events (i.e. presence of loss of consciousness, post-traumatic amnesia, vomiting) to predict prolonged symptoms.	71%	None
There is not sufficient evidence to support the clinical application of objective biomarkers (i.e. fluid, imaging) for identifying and screening for persistent symptoms after a mild TBI.	81%	None
There is not enough evidence to use prognostic tools to predict the risk of developing persistent symptoms.	81%	None
All mild TBI patients should be screened for persistent symptoms	67%	Eliminated
All mild TBI patients should receive follow-up in specialist clinics	19%	Eliminated
There should be a system for patients to be triaged to a specialist in (mild) TBI clinics based on persistent symptoms.	95%	Amended
All patients with history of 2 or more head injuries should be seen in specialist clinics regardless of whether they experience persistent symptoms.	29%	Eliminated
All patients with history of neurological conditions (i.e. migraines, epilepsy) should be seen in specialist clinics regardless of whether they experience persistent symptoms.	14%	Eliminated
All patients with history of psychological problems (i.e. depression, anxiety, PTSD) should be seen in specialist clinics regardless of whether they experience persistent symptoms.	10%	Eliminated

Karvandi E, *et al. BMJ Open* 2024; 13:e077022. doi: 10.1136/bmjopen-2023-077022

Only symptomatic patients should be seen in specialist clinics.	81%	Amended
Digital tools should be utilised to screen for persistent symptoms.	71%	Amended
Symptom scales are currently the most appropriate method available for measuring symptom burdens in patients.	71%	Amended
Symptom scales are currently the most appropriate method available to screen for persistent symptoms in patients.	71%	None
A bespoke symptom scale, such as the Rivermead or Post-Concussion Symptom Scale, should be used for screening symptoms.	80%	Amended
All patients should complete a symptom scale at the first point of contact with a clinician in the acute phase.	71%	None
Patients screening negative for PPCS on the survey at any point should be discharged from specialist service.	67%	Eliminated
Patients should be under the care of their regional mild TBI clinic until symptom resolution, and only discharged once fully recovered and returned to work, where applicable.	52%	Eliminated
The extended Glasgow Outcome Scale (eGOS) should be used to measure functional outcomes after mild TBI in routine clinical practice.	50%	Amended
The SF-36 or EQ-5D should be used to measure quality of life after mild TBI in routine clinical practice.	35%	Eliminated
A bespoke patient-reported outcome measure (PROM) should be developed for mild TBI and used in routine clinical practice	70%	None

There should be dedicated mild TBI clinics, or where not possible, TBI clinics with dedicated time to mild TBI patients.	100%	None
Specialist (mild) TBI clinics should comprise of a multidisciplinary team.	90%	None
All patients should be directed to a single bespoke online information portal on mild TBI to help with expectations and recommendations.	90%	None
Patients should be advised to rest (cognitive and physical rest) for 24-48 hours, after which they should gradually increase activity.	75%	None
Patients should be advised to rest (cognitive and physical rest) for as long as they are experiencing symptoms	25%	Eliminated
There is not sufficient evidence to support the use of advanced imaging techniques in clinical practice.	80%	None
There is not sufficient evidence to support screening for symptom subtypes. 10. Investigations and treatments should be symptom-based.	80%	Amended
All patients with persistent symptoms should complete a cognitive assessment reviewed by a neuropsychologist	-*	Amended
All clinics should have access to standardised protocols for assessing pituitary dysfunction.	90%	None
Adjusting expectation and education should be the primary initial approach when managing mild TBI, regardless of symptoms.	85%	None
Co-morbidities should be factored into clinical decision-making on management.	100%	None

Socioeconomic status and social support should be considered during clinical decision-making on management.	90%	None
Where patients are involved in sports, they should be advised to return to work or studies without symptom exacerbation before returning to unsupervised physical exercise.	70%	None
Low-intensity physical activity should be recommended as long as it does not exacerbate symptoms.	100%	None
Treatments should be assessed on a case-by-case basis.	-**	Amended

*The format of the response options for the statement was amended following voting and degree of agreement is therefore unavailable. **9 statements about symptom specific treatments were combined. The format of the response options for the statement was amended following voting and degree of agreement is therefore unavailable.
ED, Emergency Department; EQ-5D, EurQol-5 Dimensions; PPCS, Persistent Post-Concussion Symptoms; PTSD, Post Traumatic Stress Disorder; SF-36, Short-Form 36; TBI, Traumatic Brain Injury

Supplementary material 2.2 Additional statements

Statements	Response options
All patients should be screened for symptoms	2 weeks after injury<br>4 weeks after injury<br>6 weeks after injury<br>8 weeks after injury<br>10 weeks after injury<br>12 weeks after injury<br>All the above<br>Never<br>Other (open text)

Patients should be treated in primary care settings and only referred to specialist services if they do not improve within	2 weeks of injury<br>4 weeks of injury<br>6 weeks of injury<br>8 weeks of injury<br>10 weeks of injury<br>12 weeks of injury<br>Never<br>Other (open text)
Patients with persistent symptoms should first be reviewed in specialist clinic within	2 weeks of injury<br>4 weeks of injury<br>6 weeks of injury<br>8 weeks of injury<br>10 weeks of injury<br>12 weeks of injury<br>Never<br>Other (open text)
Patients should be followed-up in specialist clinic (in-person or telephone follow-up, depending on need)	Every 2 weeks until full recovery<br>Every 4 weeks until full recovery<br>Every 6 weeks until full recovery<br>Every 8 weeks until full recovery<br>Every 10 weeks until full recovery<br>Every 12 weeks until full recovery<br>None of the above, interval between reviews should increase with time as patients improve<br>None of the above, patients should only be reviewed once in clinic<br>Case-by-case assessment<br>Other (open text)
Criteria for clinic follow-up should be if they have	1 or more mild-severe symptoms<br>2 or more mild-severe symptoms<br>3 or more mild-severe symptoms

	1 or more moderate-severe symptoms<br>2 or more moderate-severe symptoms<br>3 or more moderate-severe symptoms.<br>None of the above (no set criteria) – symptom scales should always be interpreted by an experienced physician on a case-by-case basis<br>Other (open text)
The initial screening of patients should be done by	Automated system<br>Specialist nurse<br>Occupational therapist<br>Physical therapist<br>Neuropsychologist<br>Neurologist<br>Neurosurgeon<br>Neurorehabilitation specialist<br>Emergency medicine physician<br>Sport and exercise medicine physician<br>Other (open text)
The first review in specialist clinic should be done by	Automated system<br>Specialist nurse<br>Occupational therapist<br>Physical therapist<br>Neuropsychologist<br>Neurologist<br>Neurosurgeon<br>Neurorehabilitation specialist<br>Emergency medicine physician

Karvandi E, *et al. BMJ Open* 2024; 13:e077022. doi: 10.1136/bmjopen-2023-077022

	Sport and exercise medicine physician<br>Other (open text)
The first review in specialist clinic should be	Face-to-face<br>Telephone follow-up<br>Always depend on need based on the triage<br>None of the above<br>Other (open text)
New or worsening symptoms developed more than 1 month after injury should be investigated for non-head injury related causes under	The mild TBI specialist outpatient service.<br>Primary care services.<br>Other (open text)
A bespoke patient-reported outcome measure (PROM) should be developed for mild TBI and used in routine clinical practice	To measure functional outcomes<br>To measure quality of life<br>To measure symptom burden<br>All the above<br>None of the above<br>Other (open text)
At a minimum, the multidisciplinary team should consist of	Neurosurgeons<br>Neurologists<br>Neuropsychologist<br>Vestibular specialist<br>Physiotherapists<br>Specialist nurse<br>Occupational therapist<br>Neurorehabilitation specialist<br>Sport and exercise medicine physician<br>All the above<br>None of the above<br>Other (open text)

Karvandi E, *et al. BMJ Open* 2024; 13:e077022. doi: 10.1136/bmjopen-2023-077022

In addition to the core MDT, there should be a direct access to	Ophthalmologists<br>Sleep clinics<br>Headache/migraine specialist clinic<br>Internet-based Cognitive<br>Behavioural Therapy<br>Community-level services<br>All the above<br>None of the above<br>Other (open text)
Patients with the following symptom(s) should have an MRI to exclude gross structural abnormalities.	Low mood<br>Anxiety<br>Memory problems<br>Concentration problems<br>Taking longer to think<br>Restlessness<br>Irritable<br>Feeling frustrated<br>Fatigue<br>Sleep disturbance<br>Nausea<br>Dizziness (without an obvious underlying vestibular pathology)<br>Headache<br>Sensitivity to noise<br>Sensitivity to light<br>Visual symptoms (without an obvious underlying visual pathology)<br>None of the above<br>All the above<br>Case-by-case assessment

Karvandi E, *et al. BMJ Open* 2024; 13:e077022. doi: 10.1136/bmjopen-2023-077022

All patients with persistent symptoms should complete a cognitive assessment reviewed by a neuropsychologist*	In-person in clinic<br>Online in clinic<br>Online at home<br>Any of the above<br>None of the above<br>Other (open text)
Patients with the following symptom(s) should be referred to neuropsychology or psychiatry for support or cognitive rehabilitation**	Low mood<br>Anxiety<br>Memory problems<br>Concentration problems<br>Taking longer to think<br>Restlessness<br>Irritable<br>Feeling frustrated<br>Fatigue<br>Sleep disturbance<br>Nausea<br>Dizziness (without an obvious underlying vestibular pathology)<br>Headache<br>Sensitivity to noise<br>Sensitivity to light<br>Visual symptoms (without an obvious underlying visual pathology)<br>None of the above<br>All the above<br>Case-by-case assessment
Patients with the following symptom(s) should be referred for physical therapy for physical rehabilitation**	Low mood<br>Anxiety<br>Memory problems<br>Concentration problems<br>Taking longer to think

	Restlessness<br>Irritable<br>Feeling frustrated<br>Fatigue<br>Sleep disturbance<br>Nausea<br>Dizziness (without an obvious underlying vestibular pathology)<br>Headache<br>Sensitivity to noise<br>Sensitivity to light<br>Visual symptoms (without an obvious underlying visual pathology)<br>None of the above<br>All the above<br>Case-by-case assessment
Patients with the following symptom(s) should be referred for cervical rehabilitation**	Low mood<br>Anxiety<br>Memory problems<br>Concentration problems<br>Taking longer to think<br>Restlessness<br>Irritable<br>Feeling frustrated<br>Fatigue<br>Sleep disturbance<br>Nausea<br>Dizziness (without an obvious underlying vestibular pathology)<br>Headache<br>Sensitivity to noise<br>Sensitivity to light

Karvandi E, *et al. BMJ Open* 2024; 13:e077022. doi: 10.1136/bmjopen-2023-077022

	Visual symptoms (without an obvious underlying visual pathology)<br>None of the above<br>All the above<br>Case-by-case assessment
Patients with the following symptom(s) should be referred for subsymptom threshold aerobic exercise**	Low mood<br>Anxiety<br>Memory problems<br>Concentration problems<br>Taking longer to think<br>Restlessness<br>Irritable<br>Feeling frustrated<br>Fatigue<br>Sleep disturbance<br>Nausea<br>Dizziness (without an obvious underlying vestibular pathology)<br>Headache<br>Sensitivity to noise<br>Sensitivity to light<br>Visual symptoms (without an obvious underlying visual pathology)<br>None of the above<br>All the above<br>Case-by-case assessment
Patients with the following symptom(s) should be referred for vestibular rehabilitation**	Low mood<br>Anxiety<br>Memory problems<br>Concentration problems<br>Taking longer to think

Karvandi E, *et al. BMJ Open* 2024; 13:e077022. doi: 10.1136/bmjopen-2023-077022

	Restlessness<br>Irritable<br>Feeling frustrated<br>Fatigue<br>Sleep disturbance<br>Nausea<br>Dizziness (without an obvious underlying vestibular pathology)<br>Headache<br>Sensitivity to noise<br>Sensitivity to light<br>Visual symptoms (without an obvious underlying visual pathology)<br>None of the above<br>All the above<br>Case-by-case assessment
Patients with the following symptom(s) should be referred for cervical rehabilitation**	Low mood<br>Anxiety<br>Memory problems<br>Concentration problems<br>Taking longer to think<br>Restlessness<br>Irritable<br>Feeling frustrated<br>Fatigue<br>Sleep disturbance<br>Nausea<br>Dizziness (without an obvious underlying vestibular pathology)<br>Headache<br>Sensitivity to noise<br>Sensitivity to light

	Visual symptoms (without an obvious underlying visual pathology)<br>None of the above<br>All the above<br>Case-by-case assessment
Patients with the following symptom(s) should be referred for hyperbaric oxygen therapy**	Low mood<br>Anxiety<br>Memory problems<br>Concentration problems<br>Taking longer to think<br>Restlessness<br>Irritable<br>Feeling frustrated<br>Fatigue<br>Sleep disturbance<br>Nausea<br>Dizziness (without an obvious underlying vestibular pathology)<br>Headache<br>Sensitivity to noise<br>Sensitivity to light<br>Visual symptoms (without an obvious underlying visual pathology)<br>None of the above<br>All the above<br>Case-by-case assessment
Patients with the following symptom(s) should be referred for transcranial magnetic stimulation.**	Low mood<br>Anxiety<br>Memory problems<br>Concentration problems<br>Taking longer to think

	Restlessness<br>Irritable<br>Feeling frustrated<br>Fatigue<br>Sleep disturbance<br>Nausea<br>Dizziness (without an obvious underlying vestibular pathology)<br>Headache<br>Sensitivity to noise<br>Sensitivity to light<br>Visual symptoms (without an obvious underlying visual pathology)<br>None of the above<br>All the above<br>Case-by-case assessment
There is not sufficient evidence for any pharmacological interventions to be routinely prescribed for persistent symptoms after mild TBI for the following symptom(s).**	Low mood<br>Anxiety<br>Memory problems<br>Concentration problems<br>Taking longer to think<br>Restlessness<br>Irritable<br>Feeling frustrated<br>Fatigue<br>Sleep disturbance<br>Nausea<br>Dizziness (without an obvious underlying vestibular pathology)<br>Headache<br>Sensitivity to noise<br>Sensitivity to light

Karvandi E, *et al. BMJ Open* 2024; 13:e077022. doi: 10.1136/bmjopen-2023-077022

	Visual symptoms (without an obvious underlying visual pathology)<br>None of the above<br>All the above<br>Case-by-case assessment

*Statement was amended and added as consensus statements in the second round of voting.
**Statements were added to get an overview of the panel's opinion regarding specific therapies for symptom profiles. The results resulted in the final statement denoted in supplementary material 2.1.
MDT, multidisciplinary team; MRI, Magnetic Resonance Imaging; TBI, Traumatic Brain Injury

Supplementary material 3: Consensus Statement Voting Round 2

Statement included in voting round 2	Agreement	Outcome
There should be a direct patient pathway from ED to specialist outpatient services available to all patients to ensure patients with persistent symptoms will be identified early.	81%	None
There is not sufficient evidence for injury-related events (i.e. presence of loss of consciousness, post-traumatic amnesia, vomiting) to predict prolonged symptoms.	80%	None
There is not sufficient evidence to support the clinical application of objective biomarkers (i.e. fluid, imaging) for identifying and screening for persistent symptoms after a mild TBI.	90%	None
There is not enough evidence to use prognostic tools to predict the risk of developing persistent symptoms.	75%	None
There should be a system for patients to be triaged to a specialist in (mild) TBI clinics based on persistent symptoms.	100%	None

Karvandi E, *et al. BMJ Open* 2024; 13:e077022. doi: 10.1136/bmjopen-2023-077022

Mild TBI patients with persistent symptoms should be seen in specialist clinics.	90%	None
Digital tools should be utilised to screen for persistent symptoms.	89%	None
Symptom scales are currently the most appropriate method available for measuring symptom burdens in patients.	74%	None
Symptom scales are currently the most appropriate method available to screen for persistent symptoms in patients.	74%	None
A bespoke symptom scale should be used for screening symptoms.	86%	None
All patients should complete a symptom scale at the first point of contact with a clinician in the acute phase.	70%	None
A bespoke patient-reported outcome measure (PROM) should be developed for mild TBI and used in routine clinical practice.	95%	None
There should be dedicated mild TBI clinics, or where not possible, TBI clinics with dedicated time to mild TBI patients.	95%	None
Specialist (mild) TBI clinics should comprise of a multidisciplinary team.	95%	None
All patients should be directed to a single bespoke online information portal on mild TBI to help with expectations and recommendations.	81%	None
Patients should be advised to rest (cognitive and physical rest) for 24-48 hours, after which they should gradually increase activity.	75%	None
There is not sufficient evidence to support the use of advanced imaging techniques in clinical practice.	81%	None

Karvandi E, *et al. BMJ Open* 2024; 13:e077022. doi: 10.1136/bmjopen-2023-077022

There is not sufficient evidence to support screening for clinical subtypes based on symptom clusters.	67%	Eliminated
All patients with persistent symptoms should complete a cognitive assessment reviewed by a neuropsychologist.	42%	Eliminated
All clinics should have access to standardised protocols for assessing pituitary dysfunction.	76%	None
Adjusting expectation and education should be the primary initial approach when managing mild TBI, regardless of symptoms.	90%	None
Co-morbidities should be factored into clinical decision-making on management.	100%	None
Socioeconomic status and social support should be considered during clinical decision-making on management.	86%	None
Where patients are involved in sports, they should be advised to return to work or studies without symptom exacerbation before returning to unsupervised physical exercise.	62%	Eliminated
Low-intensity physical activity should be recommended as long as it does not exacerbate symptoms.	95%	None
Treatment should be symptom-based and assessed on a case-by-case basis as there is currently not sufficient evidence to support a specific treatment approach for all mild TBI patients.	95%	None

ED, Emergency Department; TBI, Traumatic Brain Injury

Karvandi E, *et al. BMJ Open* 2024; 13:e077022. doi: 10.1136/bmjopen-2023-077022
