# Supplementary material for: Specialist healthcare services for concussion/mild traumatic brain injury in England: a consensus statement using modified Delphi methodology
Source: BMJ Open. 2023 Dec 9;13(12):e077022. doi: 10.1136/bmjopen-2023-077022 (PMC10729241; doi:10.1136/bmjopen-2023-077022)
Supplement: Author's manuscript [file bmjopen-2023-077022.draft_revisions.pdf]

BMJ Open is committed to open peer review. As part of this commitment we make the peer review history of every article we publish publicly available.

When an article is published we post the peer reviewers' comments and the authors' responses online. We also post the versions of the paper that were used during peer review. These are the versions that the peer review comments apply to.

The versions of the paper that follow are the versions that were submitted during the peer review process. They are not the versions of record or the final published versions. They should not be cited or distributed as the published version of this manuscript.

BMJ Open is an open access journal and the full, final, typeset and author-corrected version of record of the manuscript is available on our site with no access controls, subscription charges or pay-per-view fees (http://bmjopen.bmj.com).

If you have any questions on BMJ Open's open peer review process please email info.bmjopen@bmj.com

BMJ Open

Specialist Healthcare Services for Concussion/Mild Traumatic Brain Injury in England: An Expert Consensus Statement

Journal:	*BMJ Open*
Manuscript ID	bmjopen-2023-077022
Article Type:	Original research
Date Submitted by the Author:	23-Jun-2023
Complete List of Authors:	Karvandi, Elika; University of Cambridge, Neurosurgery Department of Neurosurgery<br>Helmy, Adel; University of Cambridge, Department of Neurosurgery<br>Kolias, Angelos; University of Cambridge, Department of Neurosurgery<br>belli, antonio; University of Birmingham, Inflammation and Ageing<br>Ganau, Mario; University of Oxford Nuffield Department of Clinical Neurosciences<br>Gomes, Clint; Royal Liverpool University Hospital; UK Sports Institute<br>Grey, Michael; Loughborough University, School of Sport, Exercise and Health Sciences<br>Griffiths, Michael; University of Liverpool, Department of Clinical Infection, Microbiology and Immunology, Institute of Infection, Veterinary and Ecological Sciences; Alder-Hey Children's NHS Trust, Department of Paediatric Neurology<br>Griffiths, Timothy; Newcastle University, Department of Cognitive Neurology; University College London, Institute of Neurology<br>Griffiths, Philippa; Northumberland Tyne and Wear NHS Foundation Trust, Sunderland & South Tyneside Community Acquired Brain Injury Service<br>Holliman, Damian; Royal Victoria Infirmary, Department of Neurosurgery<br>Jenkins, Peter; Southampton General Hospital, Wessex Neuroscience Centre; Imperial College London<br>Jones, Ben; Leeds Beckett University - Headingley Campus, Carnegie Applied Rugby Research (CARR) centre; Rugby Football League Ltd, England Performance Unit<br>Lawrence, Tim; University of Oxford, Nuffield Department of Clinical Neurosciences<br>McLoughlin, Terence; Royal Liverpool University Hospital<br>McMahon, Catherine; The Walton Centre NHS Foundation Trust<br>Messahel, Shrouk; Alder Hey Children's Hospital NHS Foundation Trust<br>Newton, Joanne; Newcastle Upon Tyne Hospitals NHS Foundation Trust<br>Noad, Rupert; University Hospitals Plymouth NHS Trust<br>Raymont, Vanessa; University of Oxford, Department of Psychiatry<br>Sharma, Kanchan; North Bristol NHS Trust, Department of Neurology<br>Sylvester, Richard; National Hospital for Neurology and Neurosurgery; University College London, Institute of Exercise and Health<br>Tadmor, Daniel; Leeds Beckett University, Carnegie School of Sport; Leeds Rhinos Rugby League Club, Medical<br>Whitfield, Peter; University Hospitals Plymouth NHS Trust

Wilson, Mark; Imperial College Healthcare NHS Trust, Department of Neurosurgery; Imperial College London
Woodberry, Emma; University of Cambridge, Department of Neuropsychology
Parker, Michael; Love of the Game
Hutchinson, Peter; University of Cambridge, Neurosurgery

Keywords: Neurological injury < NEUROLOGY, Patient-Centered Care, PUBLIC HEALTH

SCHOLARONE™ Manuscripts

I, the Submitting Author has the right to grant and does grant on behalf of all authors of the Work (as defined in the below author licence), an exclusive licence and/or a non-exclusive licence for contributions from authors who are: i) UK Crown employees; ii) where BMJ has agreed a CC-BY licence shall apply, and/or iii) in accordance with the terms applicable for US Federal Government officers or employees acting as part of their official duties; on a worldwide, perpetual, irrevocable, royalty-free basis to BMJ Publishing Group Ltd ("BMJ") its licensees and where the relevant Journal is co-owned by BMJ to the co-owners of the Journal, to publish the Work in this journal and any other BMJ products and to exploit all rights, as set out in our licence.

The Submitting Author accepts and understands that any supply made under these terms is made by BMJ to the Submitting Author unless you are acting as an employee on behalf of your employer or a postgraduate student of an affiliated institution which is paying any applicable article publishing charge ("APC") for Open Access articles. Where the Submitting Author wishes to make the Work available on an Open Access basis (and intends to pay the relevant APC), the terms of reuse of such Open Access shall be governed by a Creative Commons licence – details of these licences and which Creative Commons licence will apply to this Work are set out in our licence referred to above.

Other than as permitted in any relevant BMJ Author's Self Archiving Policies, I confirm this Work has not been accepted for publication elsewhere, is not being considered for publication elsewhere and does not duplicate material already published. I confirm all authors consent to publication of this Work and authorise the granting of this licence.

For peer review only

Specialist Healthcare Services for Concussion/Mild Traumatic Brain Injury in England: An Expert Consensus Statement

ABSTRACT

A significant number of concussion patients experience prolonged recovery and require timely and appropriate medical attention in the outpatient setting. However, current practice in England is not standardised and the structure and processes in the healthcare system for concussion prevent delivery of best-practice clinical care.

The purpose of this work is to establish a consensus on the structure and process of healthcare services for concussion patients in England to facilitate better healthcare quality and patient outcome.

This consensus study followed the modified Delphi methodology with five phases; participant identification, item development, two rounds of voting, and a meeting to finalise the consensus statements. The pre-defined threshold for agreement was set at ≥70%. Members of the UK Concussion Network were invited to participate. The network consists of clinical specialists in head injury practising in emergency medicine, neurology, neuropsychology, neurosurgery, paediatric medicine, rehabilitation medicine and sports and exercise medicine in England.

56 items were voted on in the first round. 30 items were removed following the first voting round and 3 items were removed following the second voting round. Items were modified where appropriate. A final 18 statements reached consensus covering three main topics in specialist healthcare services for concussion; care pathway to structured follow-up, prognosis and measures of recovery, and provision of outpatient clinics.

This work presents statements on how the structure and process of care for concussion patients in England could be improved to meet their health needs. Future work will seek to implement these into the clinical pathway.

For peer review only

KEY POINT

- The recovery trajectories after a concussion varies between individuals and a significant number of patients experience prolonged recoveries
- These patients require timely and appropriate medical review and treatment
- The outpatient management of concussion remains a challenge due to the current structure and process of the healthcare system
- This is a barrier for the delivery of evidence-based best-practice clinical care in specialist outpatient clinics
- This consensus statement outlines structural and process-based factors needed to move towards standardised best-practice care in concussion in England to improve healthcare quality and patient outcome
- To our knowledge, no studies have reported on the healthcare system for concussion in England
- We engaged with various stakeholders involved in concussion care to ensure different perspectives are represented

For peer review only

INTRODUCTION

In England and Wales alone, more than a million people suffer a head injury every year (1,2). These injuries occur as a result of direct or indirect impacts to the head and are commonly encountered in sports, both at professional and grassroots levels. Injuries to the head, particularly in the context of sport, are often labelled “concussions”. There is no consensus definition for what constitutes a concussion, and it is often used to describe both the symptoms suffered following a head injury as well as the pathophysiological mechanisms causing these symptoms (3). Despite some controversies in the field, concussion is widely recognised by the public and those playing sport, and for this reason, we have used the term for the purpose of this paper. A concussion is diagnosed on clinical grounds with evidence of immediate and transient neurological dysfunction that may or may not present with a wide range of signs and symptoms, such as loss of consciousness, post-traumatic amnesia, headache and vomiting (4). However, most concussions do not present with obvious neurological signs, such as loss of consciousness, making recognition and diagnosis challenging (5–8).

The post-concussion sequelae are assumed to recover spontaneously within a few days to a couple of weeks. Yet a proportion of individuals experience persistent symptoms for months to years after injury that requires timely and appropriate medical assessment and treatment (9,10). Despite this known fact, guidance on clinical practice in the outpatient setting after the initial medial review is limited and ambiguous leaving the concussion population vulnerable to variations and sub-optimal care. There is no standardised, structured follow-up for those who experience persistent symptoms and current clinical services largely fail to meet the healthcare needs of these patients.

Nonetheless, the past two decades have seen an exponential growth in concussion research which has improved our understanding of the injury and strategies to enhance recovery and quality of care (11). Additionally, there is increasingly more awareness around the potential lasting effects of concussions, especially in sports, where there are growing concerns and calls for action (12). The government and sports’ national governing bodies are attempting to improve concussion management in all levels of sports by standardising protocols, allocating more funds for concussion research and incorporating new playing rules to reduce concussion risk and improve management (13). Consequently, the demand for care is evolving and to meet patient needs changes to clinical services are required to provide evidence-based best-

For peer review only - http://bmjopen.bmj.com/site/about/guidelines.xhtml

practice care whilst being cost-effective, delivering better healthcare value and quality and improving health outcome for concussion patients (6,14). This necessitates fundamental changes to the healthcare system in concussion care to facilitate the delivery of best-practice outpatient care. This consensus statement addresses the healthcare structure and processes in outpatient concussion care required to meet healthcare needs and improve patient outcome in the concussion population.

METHODS

Study design

A modified Delphi approach was used for this study. We followed a five-stage process; (1) panel identification, (2) literature search for initial statement development, (3-4) two voting rounds followed by item removal and modifications and (5) consensus meeting and live voting to finalise the statements. The meeting was chaired by a working group member with previous experience in the Delphi consensus method.

The guidance on Conducting and REporting DElphi Studies was followed for reporting this consensus study (15).

Panel Selection

The UK Concussion Network is a panel of clinical experts with extensive experience in head injury care in England, and setup to improve access to and quality of concussion outpatient services – for sports-related and non-sports-related concussions – and provide a network to foster research collaborations. The network consists of a multidisciplinary panel of experts from academia and clinical medicine with specialists in emergency medicine, neurology, neuropsychology, neurosurgery, paediatric medicine, rehabilitation medicine and sports and exercise medicine. The group work with Love of the Game, a national sports concussion charity, to achieve these goals.

Members of the UK Concussion Network were invited to vote and attend the final consensus meeting. The group were geographically diverse including representatives from a range of healthcare settings across England. The various specialties involved ensures a more informed decision-making for the different stages of care.

For peer review only - http://bmjopen.bmj.com/site/about/guidelines.xhtml

Evidence Review

The need for a consensus statement had been discussed in previous meetings and highlighted in work completed by the group (for publication). As the field of concussion has several contentious issues, we took a wide approach to topics covered in the initial statement development.

Statement development was guided by a literature search, topics of discussion within the network and results from previous work. The literature was searched for relevant studies, primarily published guidelines, randomised controlled trials and systematic reviews. Published clinical practice guidelines were used to aid statement development to determine whether the clinical experts agreed with published statements and their application into clinical practice in England. Similarly, systematic reviews and randomised controlled trials were used to determine whether the level of evidence was sufficient for implementation into nation-wide clinical practice.

A subgroup of network members was responsible for this stage of the process (EK, PJH, AH). The initial statements were circulated to all network members for comments on the scope, structure and wording prior to starting the official rounds of voting. Keywords used in the literature searches included concussion, mild traumatic brain injury, recovery, management, follow up, clinical practice, guidelines and therapies.

Consensus Process

Two rounds of online surveys were administered between October and December 2022 followed by a virtual meeting in February 2023. The surveys were distributed for voting using the Qualtrics XM platform (Qualtrics, Provo, UT).

All voting was anonymous to ensure uninhibited voting. The cut-off threshold for agreement was set at ≥70% for the two rounds of voting (16–18). A statement was only included if agreement was above the pre-defined threshold for consensus. Free text comments were incorporated to allow members to suggest modifications or refinements of the statements. Statements that did not meet the threshold were excluded after each round of voting and

For peer review only - http://bmjopen.bmj.com/site/about/guidelines.xhtml

statements were modified for further considerations if suggestions were made by the participants.

A virtual consensus meeting was held to finalise the statements and ensure clarity and accuracy. Statements were discussed and underwent further modifications where necessary before a final vote took place within the meeting. The threshold for agreement was raised to ≥85% for this meeting to ensure a robust degree of consensus.

RESULTS

Evidence Review

Whilst several papers were identified on therapeutic interventions, educational interventions, and biomarkers for diagnosis and prognosis in concussion, as expected the literature on healthcare processes and structures in concussion is scarce. Thus, the final statements were primarily guided by previous discussions, interpretation of how research evidence on best-practice could be formulated into the structure and process of the healthcare system, and research work by the network and patient focus group sessions (manuscript for publication).

Key papers identified during the literature review included clinical practical guidelines for concussion management (19–22) and systematic reviews on therapeutic interventions (23–28), prognosis (29–38), recovery (39,40) and biomarkers (41,42). The clinical practice guidelines included a synthesis of practical guidelines primarily focused on four published papers; Center for Disease Control and Prevention Guideline on the Diagnosis and Management of Mild Traumatic Brain Injury Among Children (2019) (43), Consensus Statement on Concussion in Sport – the 5th International Conference on Concussion in Sport (2016), Ontario Neurotrauma Foundation Guideline for Concussion/Mild Traumatic Brain Injury & Persistent Symptoms (3rd edition; 2018), and Department of Veterans Affairs/Department of Defense Clinical Practice Guideline for the Management of Concussion-Mild Traumatic Brain Injury (2016) (20).

Whilst it was agreed that covering a wide range of topics was appropriate during the initial statement development, the statements in this consensus report were narrowed down to specifically focus on the delivery of healthcare provisions through processes and structures in concussion care. This was deemed to be the most appropriate and relevant due to (a) some

For peer review only - http://bmjopen.bmj.com/site/about/guidelines.xhtml

disagreement on clinical practice and management issues in concussion among the experts, (b) determining that these statements were beyond the purpose of this group and some of this guidance would likely to be featured in the next international *Consensus Statement on Concussion in Sport*, and (c) recognition that the need for the healthcare service to be improved at a system level prior to changes in day-to-day clinical practice. These points became clear after the first round of voting and the majority of these statement were removed after this stage. The decision to remove the remaining statements on day-to-day practice was made during the final meeting.

Consensus Process

Both rounds of voting had 21 votes (75% of network members). Following the initial statement development, the first round of voting had 56 items (figure 1, supplementary material 1). This resulted in 18 statements being eliminated as they were below the threshold of agreement, 12 were eliminated as to the format of the statements did not follow a consensus process and 6 statements were amended based on comments from voting members. As a result, the second round of voting included 26 statements that were put to a vote (supplementary material 2). Three items did not reach the threshold of agreement and were removed, leaving 23 statements to be discussed during the meeting. No statements were amended at this stage.

The virtual consensus meeting resulted in four statements being eliminated, two pairs of statements were combined and one statement was added. Of the eliminated statements, three were deemed to be beyond the scope of this consensus statement. The statement added during the meeting was suggested by one member and voted on twice as the level of agreement was just below the threshold following the first vote. The statement was modified and included following the second vote as the level of agreement was above the threshold.

Figure 1: Modified Delphi rounds

This resulted in a final 18 statements (table 1), all above the raised threshold of 85% agreement. 14 statements achieved unanimous agreement. The final statements were organised into three themes; (1) care pathway to structured follow-up, (2) prognosis and measures of recovery, and (3) provision of outpatient clinics.

For peer review only - http://bmjopen.bmj.com/site/about/guidelines.xhtml

For peer review only

Table 1: Final consensus statements

	Agreement %
**1 Care Pathway to Structured Follow-up**	
Concussion patients with persistent symptoms should be able to access specialist clinics.	100%
There should be dedicated concussion clinics, or where not possible, clinics with dedicated time for concussion patients.	100%
Specialist head injury clinics should comprise a multidisciplinary team that should include, as a minimum, a clinician, neuropsychologist and vestibular physiotherapist.	100%
There should be a direct patient pathway for patients with persistent symptoms to specialist outpatient services from the ED, GP and other sources.	100%
**2 Prognosis and Measures of Recovery**	
There should be a system for patients to be triaged to a specialist in head injury clinics based on persistent symptoms.	100%
There may be a role for digital tools (e.g., platforms, apps) to be used for screening for persistent symptoms following concussion.	100%
Symptom scales can aid the assessment of symptom burden.	100%
Screening for persistent symptoms can be aided by symptom scales.	100%
A new patient-reported tool for the assessment of symptoms and outcome should be developed and validated.	90%

For peer review only - http://bmjopen.bmj.com/site/about/guidelines.xhtml

Prolonged symptoms may develop even in the absence of specific acute signs, such as loss of consciousness, post-traumatic amnesia and vomiting.	100%
There are no prognostic tools currently available with sufficient clinical accuracy to predict the risk of developing persistent symptoms.	100%
There is currently insufficient evidence to support the clinical application of objective biomarkers (i.e., fluid, imaging) for identifying and screening for persistent symptoms after a concussion.	100%
Symptomatic patients with abnormal cranial/brain imaging should be considered for follow-up in a specialist clinic.	89%
There is not yet sufficient evidence to support the routine use of advanced imaging techniques (e.g., DTI, fMRI, MEG) in clinical practice.	90%
**Provision of Outpatient Clinics**	
All patients should be directed to online information on concussion to help with expectations and recommendations.	100%
All clinics should have access to standardised protocols for assessing pituitary dysfunction.	100%
Co-morbidities, socioeconomic status and social support should be factored into clinical decision-making on management.	90%
The underlying cause(s) of persistent symptoms should be established and treated on a case-by-case basis.	100%

DTI, Diffusion Tensor Imaging; ED, Emergency Department; fMRI, functional Magnetic Resonance Imaging; GP, General Practice; MEG, Magnetoencephalography

For peer review only

Plain language summary

A plain language summary was constructed by a core subgroup (EK, AH, AK, PJAH) with comments from the rest of the network (supplementary material).

DISCUSSION

Healthcare services for concussion patients need to be transformed to advance quality of care and patient outcome. Current healthcare services fail to meet the needs of a significant number of concussion patients which results in suboptimal recovery and failure of the healthcare system to fulfil its purpose of improving quality of life through enhancing health. There is an urgent need to improve the delivery of and access to concussion care in order to reduce healthcare inequalities and errors in management.

The statements in this consensus report primarily focus on the structure and process of specialist healthcare services in concussion care. These are the components in healthcare services that facilitate the implementation and delivery of best-practice care to achieve optimal health for the target patient population. Ultimately, patient outcome is determined by the structure and process of care which describe the care context and the sum of actions in the delivery of care, respectively (44). The statements in this consensus study prioritise a patient-centric approach with a focus on timely, integrated, effective, efficient and evidence-based care and urge standardisation of these practices nationally.

Whilst the statements are aimed at all concussions, sports-related and non-sports-related concussions, the implementation of these services into the National Health Services in England requires reallocation and reprioritisation of resources, as well as a restructuring of the system. Applying these changes for the subgroup of concussions occurring in sports – at both professional and grassroot levels – could be more practical and achievable at this stage, especially accounting for the high number of all-cause concussions. For example, direct patient pathways from local sports clubs could feasibly be implemented with quick-access clinics given the manageable number of sports-related concussions. A short-term goal of the UK Concussion Network is the development of regional sports concussion clinics across the country to alleviate the burden of concussions in all levels of sports. These clinics aim to provide evidence-based care. However, it is intended that these services will ultimately be expanded and offered to all-cause concussion patients. Thus, while the implementation of these statements will primarily change clinical services for sports-related concussions in the

For peer review only - http://bmjopen.bmj.com/site/about/guidelines.xhtml

near future, the statements are directed at national health services offered to all patients in the longer term.

Clinical Implications

Care Pathway to structured follow-up

Patients with persistent symptoms require further medical attention following the initial medical review– which is typically performed in emergency departments, general practice surgeries or by sport physicians – to recover to their pre-injury level of health and function. Yet, in a CENTER-TBI study, 90% of 71 neurotrauma centres involved in the Europe-based study do not routinely schedule follow-up appointments for head injury patients discharged from the emergency department (45). In the UK, there is no care pathway to ensure access to structured follow-up which is likely a fundamental underlying cause for the unmet needs in concussion care. The absence of such a service may be attributed to the heterogeneity of concussions and the vast differences in patient needs. Concussion services need a multidisciplinary team approach to accommodate and offer effective management to all concussion patients with persistent symptoms and prolonged recovery. Appropriate resources – time, equipment and staffing – need to be allocated to these services to ensure there is capacity to meet the diversity and scale in need.

Providing timely care is fundamental to meet patients' health needs. Delaying intervention can prolong recovery further and result in poorer outcome. One study found patients who sought care more than 30 days after a concussion had worse outcome at three months compared with patients who sought care earlier (46). Concussion services should be redesigned with care pathways that offer access to structured follow-up for all concussion patients as necessary to ensure these patients receive timely and appropriate care. These statements echo the recommendations in the recent Lancet Commission on traumatic brain injury (47).

Prognosis and Measures of Recovery

Objective measures of disease and prognostic factors provide a means for directing care. One of the greatest challenges in concussion management is the lack of objective markers and the absence of reliable prognostic factors for recovery. Thus, early identification of patients at

For peer review only - http://bmjopen.bmj.com/site/about/guidelines.xhtml

risk of prolonged recovery is not possible and therefore assessing the need for follow-up for individuals at an early stage is challenging. Consequently, no predictive tool can be used reliably in the clinical setting to assess the risk of prolonged recovery and the need for follow-up in the outpatient setting. Even patients without marked acute signs and symptoms are at risk of prolonged recovery and should not be dismissed inadvertently. Of note, the panel recommends that patients with abnormal scans during the acute phase should always be offered follow-up as there is significant observable pathology during the acute phase to warrant further medical assessment. However, we suggest that anything beyond standard structural imaging modalities – to rule out other pathologies – should not be used in the clinical setting. Whilst advanced imaging methods are showing promise in research, they are yet to demonstrate reliable clinical utility and we do not recommend using these tools in clinical practice.

In the absence of validated objective measures, ongoing self-reporting is currently the most reliable primary indicator of health status and method for identification of patients with persistent symptoms. Of note, underreporting of concussion is a concern that needs to be addressed and is particularly prevalent in professional and community sports (48). To filter out people who have recovered and identify individuals who need further medical review, we recommend a screening system. This would need to be cost-effective and scalable due to the size of the concussion population. We suggest digital tools as a platform for the screening system as they offer a remote, convenient, efficient and scalable solution (49). Once set up, costs remain comparatively low whilst also overcoming traditional barriers related to geographical, financial and resource limitations. Such a system could also be efficiently and effectively used to triage patients to the most appropriate clinician in the multidisciplinary clinic based on patient-reported symptoms and disabilities.

Standardising evaluation of recovery in the absence of objective markers is challenging. However, symptom scales may aid assessment of symptom burden and provide a means for screening patients, particularly as history taking and patient-reported symptoms are currently the only methods for measuring recovery. Long-term, we recommend developing and validating a patient-reported outcome measure for the concussion population to help assess recovery and determine care needs. This may include assessment of symptom burden, functional status, return to work or activity and quality of life. However, the specifics need to be determined in an appropriate study.

For peer review only - http://bmjopen.bmj.com/site/about/guidelines.xhtml

In the era of patient-centric and value-based healthcare, patient-reported outcome measures are increasingly important to capture the patient's perspective of their health. Even when objective measures are identified in the future, these tools should be utilised to capture a holistic patient-centric assessment of health status and recovery.

Provision of Outpatient Clinics

Providing early information and awareness is essential to managing expectations and can enhance recovery. A TRACK-TBI study found that only 42% of patients reported receiving head injury information at discharge from the emergency department (50). Providing proper discharge information can be challenging in the emergency department due to the busy environment in these clinical settings and the cognitive state of the patients initially after injury which may prevent them from comprehending and retaining information (51). Thus, we suggest directing patients to a single online platform for evidence-based information. The online modality allows anyone to access the information at any time, provides the same information to all regardless of geographical region and is easier to keep up to date with the evidence, which is particularly important in a fast-evolving field.

For patients with persistent symptoms, determining the most appropriate therapy can be challenging, especially in the absence of sufficiently strong evidence on therapeutic efficacy and timing of interventions. Given the vast heterogeneity in cases, no single treatment plan can be utilised for all concussion patients and clinicians must evaluate the most appropriate intervention(s) on a case-by-case basis. Whilst further research is required in this domain some therapeutic interventions have shown promise. Recommendations for therapeutic approaches are beyond the scope of this consensus statement and we recommend referring to other literature on this topic (23–28). Biopsychosocial factors should be considered when assessing clinical and social care needs as these have been shown consistently to influence patient recovery (35).

Lastly, we suggest that concussion clinics should have access to a standardised protocol for assessing pituitary function. Pituitary dysfunction can result in a range of different symptoms, many of which overlap with concussion symptoms, including fatigue, anxiety and mood disturbances. The literature on the incidence of pituitary dysfunction after concussions and

For peer review only - http://bmjopen.bmj.com/site/about/guidelines.xhtml

screening protocols is still an area of active research. We suggest referring to a guidance paper by the British Neurotrauma Group on screening and management of pituitary dysfunction after traumatic brain injury (52).

Limitations

The literature on concussion, though rapidly evolving, is contentious and there are still numerous areas of disagreement among experts. As discussed above, a wide approach was taken for the initial statement development which was later narrowed to focus on the healthcare structure and processes, in part. It cannot be excluded that some disagreement on statements was in part due to differences in definitions and terminology use despite probable broad agreement on the statement in principle. However, establishing agreement on terminology is a different consensus process on its own and was beyond the scope of this study.

We engaged with different stakeholders including academics, clinicians from different specialties and charities, and drew from previous patient-focus group sessions (unpublished). To ensure more robust statements, we chose a high threshold for consensus. Whilst these statements reached agreement in this cohort of experts, we cannot exclude the possibility or to what degree the results are influenced by the experience and expertise of these individuals both for the included and excluded statements. However, due to the high level of agreement and consistency with the literature, we believe the statements can be integrated reliably in the national healthcare system.

CONCLUSION

This consensus statement outlines structural and process-based factors needed to move towards standardised best-practice care in concussion. This includes a final 18 statements on the care pathway for structured follow-up, prognosis and measures of recovery and provision of outpatient clinics. The statements are aimed at both the sports-related and all-cause concussion populations, however, they should be implemented for sports-related concussions as the first step before expanding to all concussion patients given the greater feasibility of this approach. Further research and stakeholder engagement is needed to determine best-practice clinical concussion management in outpatient clinics and the recommendations in this

For peer review only - http://bmjopen.bmj.com/site/about/guidelines.xhtml
For peer review only

statement provide the structures and processes whereby best-practice patient-centred care can be delivered in a timely, integrated, efficient and effective manner.

Acknowledgements

We would like to thank Love of the Game for their support with this work. Peter Hutchinson would also like to thank the NIHR Senior Investigator Award, Cambridge Biomedical Research Centre, Medtech Brain Injury Co-operative, Global Health Research Group on Acquired Brain and Spine Injury and Royal College of Surgeons of England.

REFERENCES

1. NICE guideline. Head injury: assessment and early management. 2023.
2. NICE. NATIONAL INSTITUTE FOR HEALTH AND CARE EXCELLENCE Health and social care directorate Quality standards and indicators. Briefing paper. [Internet]. 2014 [cited 2023 Mar 20]. Available from: https://www.nice.org.uk/guidance/qs74/documents/head-injury-briefing-paper2
3. Sharp DJ, Jenkins PO. Concussion is confusing us all. Pract Neurol [Internet]. 2015;15(3):172–86. Available from: https://www.scopus.com/inward/record.uri?eid=2-s2.0-84929330344&doi=10.1136%2fpractneurol-2015-001087&partnerID=40&md5=c2ae641c33158dcbe06dc0b311be793f
4. Levin HS, Diaz-Arrastia RR. Diagnosis, prognosis, and clinical management of mild traumatic brain injury. Lancet Neurol. 2015 May;14(5):506–17.
5. Marshall SW, Guskiewicz KM, Shankar V, McCrea M, Cantu RC. Epidemiology of sports-related concussion in seven US high school and collegiate sports. Inj Epidemiol. 2015;2(1).
6. Langer L, Levy C, Bayley M. Increasing Incidence of Concussion: True Epidemic or Better Recognition? Journal of Head Trauma Rehabilitation. 2020 Jan;35(1):E60–6.
7. McCrea M, Guskiewicz KM, Marshall SW, Barr W, Randolph C, Cantu RC, et al. Acute Effects and Recovery Time Following Concussion in Collegiate Football Players. JAMA. 2003 Nov 19;290(19):2556.
8. McCrory P, Meeuwisse WH, Echemendia RJ, Iverson GL, Dvořák J, Kutcher JS. What is the lowest threshold to make a diagnosis of concussion? Br J Sports Med. 2013 Apr 11;47(5):268–71.

For peer review only

For peer review only - http://bmjopen.bmj.com/site/about/guidelines.xhtml

9. Voormolen DC, Cnossen MC, Polinder S, von Steinbuechel N, Vos PE, Haagsma JA. Divergent Classification Methods of Post-Concussion Syndrome after Mild Traumatic Brain Injury: Prevalence Rates, Risk Factors, and Functional Outcome. J Neurotrauma. 2018 Jun;35(11):1233–41.
10. Clark CN, Edwards MJ, Ong BE, Goodliffe L, Ahmad H, Dilley MD, et al. Reframing postconcussional syndrome as an interface disorder of neurology, psychiatry and psychology. Brain. 2022;145(6).
11. National Institute for Health and Excellence. Practical steps to improving the quality of care and services using NICE guidance. 2018.
12. Wiebe DJ, Comstock RD, Nance ML. Concussion research: a public health priority. Injury Prevention. 2011 Feb 1;17(1):69–70.
13. UK Government. UK Concussion Guidelines for Non-Elite (Grassroots) Sport. 2023.
14. Veliz P, McCabe SE, Eckner JT, Schulenberg JE. Trends in the Prevalence of Concussion Reported by US Adolescents, 2016-2020. JAMA. 2021 May 4;325(17):1789.
15. Jünger S, Payne SA, Brine J, Radbruch L, Brearley SG. Guidance on Conducting and REporting DElphi Studies (CREDES) in palliative care: Recommendations based on a methodological systematic review. Vol. 31, Palliative Medicine. 2017.
16. Diamond IR, Grant RC, Feldman BM, Pencharz PB, Ling SC, Moore AM, et al. Defining consensus: A systematic review recommends methodologic criteria for reporting of Delphi studies. J Clin Epidemiol. 2014 Apr;67(4):401–9.
17. Slade SC, Dionne CE, Underwood M, Buchbinder R. Standardised method for reporting exercise programmes: protocol for a modified Delphi study. BMJ Open. 2014 Dec 30;4(12):e006682.
18. Vogel C, Zwolinsky S, Griffiths C, Hobbs M, Henderson E, Wilkins E. A Delphi study to build consensus on the definition and use of big data in obesity research. Int J Obes. 2019 Dec 17;43(12):2573–86.
19. Giza CC, Kutcher JS, Ashwal S, Barth J, Getchius TSD, Gioia GA, et al. Summary of evidence-based guideline update: Evaluation and management of concussion in sports: Report of the Guideline Development Subcommittee of the American Academy of Neurology. Neurology. 2013 Jun 11;80(24):2250–7.
20. Silverberg ND, Iaccarino MA, Panenka WJ, Iverson GL, McCulloch KL, Dams-O'Connor K, et al. Management of Concussion and Mild Traumatic Brain Injury: A Synthesis of Practice Guidelines. Arch Phys Med Rehabil. 2020 Feb;101(2):382–93.

For peer review only - http://bmjopen.bmj.com/site/about/guidelines.xhtml
For peer review only

21. Marshall S, Bayley M, McCullagh S, Velikonja D, Berrigan L, Ouchterlony D, et al. Updated clinical practice guidelines for concussion/mild traumatic brain injury and persistent symptoms. Brain Inj. 2015 May 12;29(6):688–700.

22. Quatman-Yates CC, Hunter-Giordano A, Shimamura KK, Landel R, Alsalaheen BA, Hanke TA, et al. Physical Therapy Evaluation and Treatment After Concussion/Mild Traumatic Brain Injury. Journal of Orthopaedic & Sports Physical Therapy. 2020 Apr;50(4):CPG1–73.

23. Boussard CN de, Holm LW, Cancelliere C, Godbolt AK, Boyle E, Stålnacke BM, et al. Nonsurgical Interventions After Mild Traumatic Brain Injury: A Systematic Review. Results of the International Collaboration on Mild Traumatic Brain Injury Prognosis. Arch Phys Med Rehabil. 2014 Mar;95(3):S257–64.

24. Schneider KJ, Leddy JJ, Guskiewicz KM, Seifert T, McCrea M, Silverberg ND, et al. Rest and treatment/rehabilitation following sport-related concussion: a systematic review. Br J Sports Med. 2017 Jun;51(12):930–4.

25. Murray DA, Meldrum D, Lennon O. Can vestibular rehabilitation exercises help patients with concussion? A systematic review of efficacy, prescription and progression patterns. Br J Sports Med. 2017 Mar;51(5):442–51.

26. Lal A, Kolakowsky-Hayner SA, Ghajar J, Balamane M. The Effect of Physical Exercise After a Concussion: A Systematic Review and Meta-analysis. Am J Sports Med. 2018 Mar 1;46(3):743–52.

27. Reid SA, Farbenblum J, McLeod S. Do physical interventions improve outcomes following concussion: a systematic review and meta-analysis? Br J Sports Med. 2022 Mar;56(5):292–8.

28. Heslot C, Azouvi P, Perdrieau V, Granger A, Lefèvre-Dognin C, Cogné M. A Systematic Review of Treatments of Post-Concussion Symptoms. J Clin Med. 2022 Oct 21;11(20):6224.

29. Rausa VC, Anderson V, Babl FE, Takagi M. Predicting Concussion Recovery in Children and Adolescents in the Emergency Department. Curr Neurol Neurosci Rep. 2018 Nov 19;18(11):78.

30. Mercier E, Tardif PA, Cameron PA, Émond M, Moore L, Mitra B, et al. Prognostic value of neuron-specific enolase (NSE) for prediction of post-concussion symptoms following a mild traumatic brain injury: a systematic review. Brain Inj. 2018 Jan 2;32(1):29–40.

For peer review only - http://bmjopen.bmj.com/site/about/guidelines.xhtml

31. Zetterberg H, Morris HR, Hardy J, Blennow K. Update on fluid biomarkers for concussion. Concussion. 2016 Dec;1(3):CNC12.

32. McCrea M, Meier T, Huber D, Ptito A, Bigler E, Debert CT, et al. Role of advanced neuroimaging, fluid biomarkers and genetic testing in the assessment of sport-related concussion: a systematic review. Br J Sports Med. 2017 Jun;51(12):919–29.

33. O'Connell B, Kelly ÁM, Mockler D, Orešič M, Denvir K, Farrell G, et al. Use of Blood Biomarkers in the Assessment of Sports-Related Concussion—A Systematic Review in the Context of Their Biological Significance. Clinical Journal of Sport Medicine. 2018 Nov;28(6):561–71.

34. Iverson GL, Gardner AJ, Terry DP, Ponsford JL, Sills AK, Broshek DK, et al. Predictors of clinical recovery from concussion: a systematic review. Br J Sports Med. 2017 Jun 31;51(12):941–8.

35. van Ierssel J, Pennock KF, Sampson M, Zemek R, Caron JG. Which psychosocial factors are associated with return to sport following concussion? A systematic review. J Sport Health Sci. 2022 Jul;11(4):438–49.

36. Karantali E, Kazis D, McKenna J, Chatzikonstantinou S, Petridis F, Mavroudis I. Neurofilament light chain in patients with a concussion or head impacts: a systematic review and meta-analysis. European Journal of Trauma and Emergency Surgery. 2022 Jun 18;48(3):1555–67.

37. Silverberg ND, Gardner AJ, Brubacher JR, Panenka WJ, Li JJ, Iverson GL. Systematic Review of Multivariable Prognostic Models for Mild Traumatic Brain Injury. J Neurotrauma. 2015 Apr 15;32(8):517–26.

38. Cancelliere C, Hincapié CA, Keightley M, Godbolt AK, Côté P, Kristman VL, et al. Systematic Review of Prognosis and Return to Play After Sport Concussion: Results of the International Collaboration on Mild Traumatic Brain Injury Prognosis. Arch Phys Med Rehabil. 2014 Mar;95(3):S210–29.

39. Kamins J, Bigler E, Covassin T, Henry L, Kemp S, Leddy JJ, et al. What is the physiological time to recovery after concussion? A systematic review. Br J Sports Med. 2017 Jun;51(12):935–40.

40. Manley G, Gardner AJ, Schneider KJ, Guskiewicz KM, Bailes J, Cantu RC, et al. A systematic review of potential long-term effects of sport-related concussion. Br J Sports Med. 2017 Jun;51(12):969–77.

For peer review only - http://bmjopen.bmj.com/site/about/guidelines.xhtml

41. Lindsey HM, Hodges CB, Greer KM, Wilde EA, Merkley TL. Diffusion-Weighted Imaging in Mild Traumatic Brain Injury: A Systematic Review of the Literature. Neuropsychol Rev. 2023 Mar 15;33(1):42–121.
42. Khong E, Odenwald N, Hashim E, Cusimano MD. Diffusion Tensor Imaging Findings in Post-Concussion Syndrome Patients after Mild Traumatic Brain Injury: A Systematic Review. Front Neurol. 2016 Sep 19;7.
43. Lumba-Brown A, Yeates KO, Sarmiento K, Breiding MJ, Haegerich TM, Gioia GA, et al. Centers for Disease Control and Prevention Guideline on the Diagnosis and Management of Mild Traumatic Brain Injury Among Children. JAMA Pediatr. 2018 Nov 5;172(11):e182853.
44. NHS England and NHS Improvement. A model for measuring quality care. 2021.
45. Foks KA, Cnossen MC, Dippel DWJ, Maas AIR, Menon D, van der Naalt J, et al. Management of Mild Traumatic Brain Injury at the Emergency Department and Hospital Admission in Europe: A Survey of 71 Neurotrauma Centers Participating in the CENTER-TBI Study. J Neurotrauma. 2017 Sep;34(17):2529–35.
46. Bunt SC, LoBue C, Hynan LS, Didehbani N, Stokes M, Miller SM, et al. Early vs. delayed evaluation and persisting concussion symptoms during recovery in adults. Clin Neuropsychol. 2022 Sep 9;1–18.
47. Maas AIR, Menon DK, Manley GT, Abrams M, Åkerlund C, Andelic N, et al. Traumatic brain injury: progress and challenges in prevention, clinical care, and research. Lancet Neurol. 2022 Nov;21(11):1004–60.
48. Longworth T, McDonald A, Cunningham C, Khan H, Fitzpatrick J. Do rugby league players under-report concussion symptoms? A cross-sectional study of elite teams based in Australia. BMJ Open Sport Exerc Med. 2021 Jan 19;7(1):e000860.
49. NIHR. What is digital health technology and what can it do for me? NIHR. 2022.
50. Seabury SA, Gaudette É, Goldman DP, Markowitz AJ, Brooks J, McCrea MA, et al. Assessment of Follow-up Care After Emergency Department Presentation for Mild Traumatic Brain Injury and Concussion. JAMA Netw Open. 2018 May 25;1(1):e180210.
51. Bergman K, Louis S. Discharge Instructions for Concussion: Are We Meeting the Patient Needs? Journal of Trauma Nursing. 2016 Nov;23(6):327–33.
52. Tan CL, Alavi SA, Baldeweg SE, Belli A, Carson A, Feeney C, et al. The screening and management of pituitary dysfunction following traumatic brain injury in adults:

For peer review only

British Neurotrauma Group guidance. J Neurol Neurosurg Psychiatry. 2017 Nov;88(11):971–81.

For peer review only

Figure 1: Modified Delphi rounds

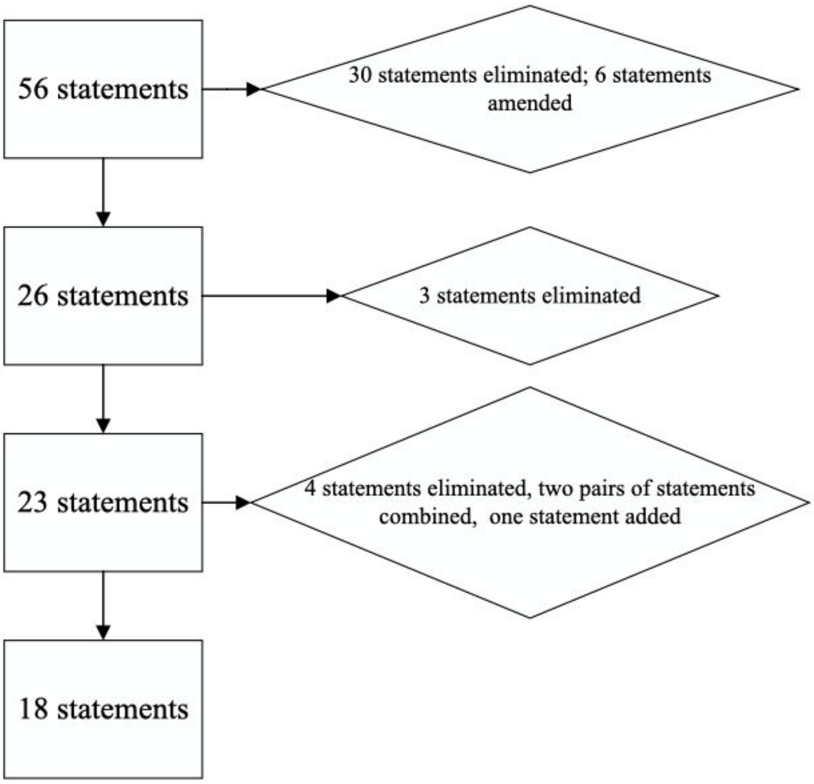

For peer review only

Supplementary material 1: Consensus Statement Voting Round 1

**Statement**	**Agreement**	**Outcome**
There should be a direct patient pathway from ED to specialist outpatient services available to all patients to ensure patients with persistent symptoms will be identified early.	71%	None
There is not sufficient evidence for injury-related events (i.e. presence of loss of consciousness, post-traumatic amnesia, vomiting) to predict prolonged symptoms.	71%	None
There is not sufficient evidence to support the clinical application of objective biomarkers (i.e. fluid, imaging) for identifying and screening for persistent symptoms after a mild TBI.	81%	None
There is not enough evidence to use prognostic tools to predict the risk of developing persistent symptoms.	81%	None
All mild TBI patients should be screened for persistent symptoms	67%	Eliminated
All mild TBI patients should receive follow-up in specialist clinics	19%	Eliminated
There should be a system for patients to be triaged to a specialist in (mild) TBI clinics based on persistent symptoms.	95%	Amended
All patients with history of 2 or more head injuries should be seen in specialist clinics regardless of whether they experience persistent symptoms.	29%	Eliminated
All patients with history of neurological conditions (i.e. migraines, epilepsy) should be seen in specialist clinics regardless of whether they experience persistent symptoms.	14%	Eliminated

All patients with history of psychological problems (i.e. depression, anxiety, PTSD) should be seen in specialist clinics regardless of whether they experience persistent symptoms.	10%	Eliminated
Only symptomatic patients should be seen in specialist clinics.	81%	Amended
Digital tools should be utilised to screen for persistent symptoms.	71%	Amended
Symptom scales are currently the most appropriate method available for measuring symptom burdens in patients.	71%	Amended
Symptom scales are currently the most appropriate method available to screen for persistent symptoms in patients.	71%	None
A bespoke symptom scale, such as the Rivermead or Post-Concussion Symptom Scale, should be used for screening symptoms.	80%	Amended
All patients should complete a symptom scale at the first point of contact with a clinician in the acute phase.	71%	None
Patients screening negative for PPCS on the survey at any point should be discharged from specialist service.	67%	Eliminated
Patients should be under the care of their regional mild TBI clinic until symptom resolution, and only discharged once fully recovered and returned to work, where applicable.	52%	Eliminated
The extended Glasgow Outcome Scale (eGOS) should be used to measure functional outcomes after mild TBI in routine clinical practice.	50%	Amended

For peer review only

The SF-36 or EQ-5D should be used to measure quality of life after mild TBI in routine clinical practice.	35%	Eliminated
A bespoke patient-reported outcome measure (PROM) should be developed for mild TBI and used in routine clinical practice	70%	None
There should be dedicated mild TBI clinics, or where not possible, TBI clinics with dedicated time to mild TBI patients.	100%	None
Specialist (mild) TBI clinics should comprise of a multidisciplinary team.	90%	None
All patients should be directed to a single bespoke online information portal on mild TBI to help with expectations and recommendations.	90%	None
Patients should be advised to rest (cognitive and physical rest) for 24-48 hours, after which they should gradually increase activity.	75%	None
Patients should be advised to rest (cognitive and physical rest) for as long as they are experiencing symptoms	25%	Eliminated
There is not sufficient evidence to support the use of advanced imaging techniques in clinical practice.	80%	None
There is not sufficient evidence to support screening for symptom subtypes. 10. Investigations and treatments should be symptom-based.	80%	Amended
All patients with persistent symptoms should complete a cognitive assessment reviewed by a neuropsychologist	-*	Amended
All clinics should have access to standardised protocols for assessing pituitary dysfunction.	90%	None

For peer review only

Adjusting expectation and education should be the primary initial approach when managing mild TBI, regardless of symptoms.	85%	None
Co-morbidities should be factored into clinical decision-making on management.	100%	None
Socioeconomic status and social support should be considered during clinical decision-making on management.	90%	None
Where patients are involved in sports, they should be advised to return to work or studies without symptom exacerbation before returning to unsupervised physical exercise.	70%	None
Low-intensity physical activity should be recommended as long as it does not exacerbate symptoms.	100%	None
Treatments should be assessed on a case-by-case basis.	-**	Amended

*The format of the response options for the statement was amended following voting and degree of agreement is therefore unavailable. **9 statements about symptom specific treatments were combined. The format of the response options for the statement was amended following voting and degree of agreement is therefore unavailable.

Supplementary material 2: Consensus Statement Voting Round 2

**Statement included in voting round 2**	**Agreement**	**Outcome**
There should be a direct patient pathway from ED to specialist outpatient services available to all patients to ensure patients with persistent symptoms will be identified early.	81%	None
There is not sufficient evidence for injury-related events (i.e. presence of loss of consciousness, post-traumatic amnesia, vomiting) to predict prolonged symptoms.	80%	None

For peer review only

There is not sufficient evidence to support the clinical application of objective biomarkers (i.e. fluid, imaging) for identifying and screening for persistent symptoms after a mild TBI.	90%	None
There is not enough evidence to use prognostic tools to predict the risk of developing persistent symptoms.	75%	None
There should be a system for patients to be triaged to a specialist in (mild) TBI clinics based on persistent symptoms.	100%	None
Mild TBI patients with persistent symptoms should be seen in specialist clinics.	90%	None
Digital tools should be utilised to screen for persistent symptoms.	89%	None
Symptom scales are currently the most appropriate method available for measuring symptom burdens in patients.	74%	None
Symptom scales are currently the most appropriate method available to screen for persistent symptoms in patients.	74%	None
A bespoke symptom scale should be used for screening symptoms.	86%	None
All patients should complete a symptom scale at the first point of contact with a clinician in the acute phase.	70%	None
A bespoke patient-reported outcome measure (PROM) should be developed for mild TBI and used in routine clinical practice.	95%	None
There should be dedicated mild TBI clinics, or where not possible, TBI clinics with dedicated time to mild TBI patients.	95%	None
Specialist (mild) TBI clinics should comprise of a multidisciplinary team.	95%	None

For peer review only

All patients should be directed to a single bespoke online information portal on mild TBI to help with expectations and recommendations.	81%	None
Patients should be advised to rest (cognitive and physical rest) for 24-48 hours, after which they should gradually increase activity.	75%	None
There is not sufficient evidence to support the use of advanced imaging techniques in clinical practice.	81%	None
There is not sufficient evidence to support screening for clinical subtypes based on symptom clusters.	67%	Eliminated
All patients with persistent symptoms should complete a cognitive assessment reviewed by a neuropsychologist.	42%	Eliminated
All clinics should have access to standardised protocols for assessing pituitary dysfunction.	76%	None
Adjusting expectation and education should be the primary initial approach when managing mild TBI, regardless of symptoms.	90%	None
Co-morbidities should be factored into clinical decision-making on management.	100%	None
Socioeconomic status and social support should be considered during clinical decision-making on management.	86%	None
Where patients are involved in sports, they should be advised to return to work or studies without symptom exacerbation before returning to unsupervised physical exercise.	62%	Eliminated
Low-intensity physical activity should be recommended as long as it does not exacerbate symptoms.	95%	None

For peer review only

Treatment should be symptom-based and assessed on a case-by-case basis as there is currently not sufficient evidence to support a specific treatment approach for all mild TBI patients.	95%	None

For peer review only

Rationale for the choice of the Delphi technique	Report on page(s) in main document
1. *Justification.* The choice of the Delphi technique as a method of systematically collating expert consultation and building consensus needs to be well justified. When selecting the method to answer a particular research question, it is important to keep in mind its constructivist nature	1-2
Planning and design	
2. *Planning and process.* The Delphi technique is a flexible method and can be adjusted to the respective research aims and purposes. Any modifications should be justified by a rationale and be applied systematically and rigorously	2-4
3. *Definition of consensus.* Unless not reasonable due to the explorative nature of the study, an a priori criterion for consensus should be defined. This includes a clear and transparent guide for action on (a) how to proceed with certain items or topics in the next survey round, (b) the required threshold to terminate the Delphi process and (c) procedures to be followed when consensus is (not) reached after one or more iterations	3-4
Study conduct	
4. *Informational input.* All material provided to the expert panel at the outset of the project and throughout the Delphi process should be carefully reviewed and piloted in advance in order to examine the effect on experts' judgements and to prevent bias	3
5. *Prevention of bias.* Researchers need to take measures to avoid directly or indirectly influencing the experts' judgements. If one or more members of the research team have a conflict of interest, entrusting an independent researcher with the main coordination of the Delphi study is advisable	2
6. *Interpretation and processing of results.* Consensus does not necessarily imply the 'correct' answer or judgement; (non)consensus and stable disagreement provide informative insights and highlight differences in perspectives concerning the topic in question	3-4
Reporting	
7. *Purpose and rationale.* The purpose of the study should be clearly defined and demonstrate the appropriateness of the use of the Delphi technique as a method to achieve the research aim. A rationale for the choice of the Delphi technique as the most suitable method needs to be provided	1-2
8. *Expert panel.* Criteria for the selection of experts and transparent information on recruitment of the expert panel, socio-demographic details including information on expertise regarding the topic in question, (non)response and response rates over the ongoing iterations should be reported	2
9. *Description of the methods.* The methods employed need to be comprehensible; this includes information on preparatory steps (How was available evidence on the topic in question synthesised?), piloting of material and survey instruments, design of the survey instrument(s), the number and design of survey rounds, methods of data analysis,	2-4

processing and synthesis of experts' responses to inform the subsequent survey round and methodological decisions taken by the research team throughout the process	
10. *Procedure.* Flow chart to illustrate the stages of the Delphi process, including a preparatory phase, the actual 'Delphi rounds', interim steps of data processing and analysis, and concluding steps	2
11. *Definition and attainment of consensus.* It needs to be comprehensible to the reader how consensus was achieved throughout the process, including strategies to deal with non-consensus	3-4
12. *Results.* Reporting of results for each round separately is highly advisable in order to make the evolving of consensus over the rounds transparent. This includes figures showing the average group response, changes between rounds, as well as any modifications of the survey instrument such as deletion, addition or modification of survey items based on previous rounds	4-8
13. *Discussion of limitations.* Reporting should include a critical reflection of potential limitations and their impact of the resulting guidance	12-13
14. *Adequacy of conclusions.* The conclusions should adequately reflect the outcomes of the Delphi study with a view to the scope and applicability of the resulting practice guidance	13
15. *Publication and dissemination.* The resulting guidance should be clearly identifiable from the publication, including recommendations for transfer into practice and implementation. If the publication does not allow for a detailed presentation of either the resulting practice guidance or the methodological features of the applied Delphi technique, or both, reference to a more detailed presentation elsewhere should be made (e.g. availability of the full guideline from the authors or online; publication of a separate paper reporting on methodological details and particularities of the process (e.g. persistent disagreement and controversy on certain issues)). A dissemination plan should include endorsement of the guidance by professional associations and health care authorities to facilitate implementation	8-12

only

BMJ Open

Specialist Healthcare Services for Concussion/Mild Traumatic Brain Injury in England: A Consensus Statement using Modified Delphi Methodology

Journal:	*BMJ Open*
Manuscript ID	bmjopen-2023-077022.R1
Article Type:	Original research
Date Submitted by the Author:	18-Oct-2023
Complete List of Authors:	Karvandi, Elika; University of Cambridge, Neurosurgery Department of Neurosurgery<br>Helmy, Adel; University of Cambridge, Department of Neurosurgery<br>Kolias, Angelos; University of Cambridge, Department of Neurosurgery<br>belli, antonio; University of Birmingham, Inflammation and Ageing<br>Ganau, Mario; University of Oxford Nuffield Department of Clinical Neurosciences<br>Gomes, Clint; Royal Liverpool University Hospital; UK Sports Institute<br>Grey, Michael; Loughborough University, School of Sport, Exercise and Health Sciences<br>Griffiths, Michael; University of Liverpool, Department of Clinical Infection, Microbiology and Immunology, Institute of Infection, Veterinary and Ecological Sciences; Alder-Hey Children's NHS Trust, Department of Paediatric Neurology<br>Griffiths, Timothy; Newcastle University, Department of Cognitive Neurology; University College London, Institute of Neurology<br>Griffiths, Philippa; Northumberland Tyne and Wear NHS Foundation Trust, Sunderland & South Tyneside Community Acquired Brain Injury Service<br>Holliman, Damian; Royal Victoria Infirmary, Department of Neurosurgery<br>Jenkins, Peter; Southampton General Hospital, Wessex Neuroscience Centre; Imperial College London<br>Jones, Ben; Leeds Beckett University - Headingley Campus, Carnegie Applied Rugby Research (CARR) centre; Rugby Football League Ltd, England Performance Unit<br>Lawrence, Tim; University of Oxford, Nuffield Department of Clinical Neurosciences<br>McLoughlin, Terence; Royal Liverpool University Hospital<br>McMahon, Catherine; The Walton Centre NHS Foundation Trust<br>Messahel, Shrouk; Alder Hey Children's Hospital NHS Foundation Trust<br>Newton, Joanne; Newcastle Upon Tyne Hospitals NHS Foundation Trust<br>Noad, Rupert; University Hospitals Plymouth NHS Trust<br>Raymont, Vanessa; University of Oxford, Department of Psychiatry<br>Sharma, Kanchan; North Bristol NHS Trust, Department of Neurology<br>Sylvester, Richard; National Hospital for Neurology and Neurosurgery; University College London, Institute of Exercise and Health<br>Tadmor, Daniel; Leeds Beckett University, Carnegie School of Sport; Leeds Rhinos Rugby League Club, Medical<br>Whitfield, Peter; University Hospitals Plymouth NHS Trust

	Wilson, Mark; Imperial College Healthcare NHS Trust, Department of Neurosurgery; Imperial College London<br>Woodberry, Emma; University of Cambridge, Department of Neuropsychology<br>Parker, Michael; Love of the Game<br>Hutchinson, Peter; University of Cambridge, Neurosurgery

**Primary Subject Heading**:	Health services research
Secondary Subject Heading:	Public health, Neurology
Keywords:	Neurological injury < NEUROLOGY, Patient-Centered Care, PUBLIC HEALTH

I, the Submitting Author has the right to grant and does grant on behalf of all authors of the Work (as defined in the below author licence), an exclusive licence and/or a non-exclusive licence for contributions from authors who are: i) UK Crown employees; ii) where BMJ has agreed a CC-BY licence shall apply, and/or iii) in accordance with the terms applicable for US Federal Government officers or employees acting as part of their official duties; on a worldwide, perpetual, irrevocable, royalty-free basis to BMJ Publishing Group Ltd ("BMJ") its licensees and where the relevant Journal is co-owned by BMJ to the co-owners of the Journal, to publish the Work in this journal and any other BMJ products and to exploit all rights, as set out in our licence.

The Submitting Author accepts and understands that any supply made under these terms is made by BMJ to the Submitting Author unless you are acting as an employee on behalf of your employer or a postgraduate student of an affiliated institution which is paying any applicable article publishing charge ("APC") for Open Access articles. Where the Submitting Author wishes to make the Work available on an Open Access basis (and intends to pay the relevant APC), the terms of reuse of such Open Access shall be governed by a Creative Commons licence – details of these licences and which Creative Commons licence will apply to this Work are set out in our licence referred to above.

Other than as permitted in any relevant BMJ Author's Self Archiving Policies, I confirm this Work has not been accepted for publication elsewhere, is not being considered for publication elsewhere and does not duplicate material already published. I confirm all authors consent to publication of this Work and authorise the granting of this licence.

For peer review only

Specialist Healthcare Services for Concussion/Mild Traumatic Brain Injury in England: A Consensus Statement using Modified Delphi Methodology

ABSTRACT

Objective: To establish a consensus on the structure and process of healthcare services for concussion patients in England to facilitate better healthcare quality and patient outcome.

Design: This consensus study followed the modified Delphi methodology with five phases; participant identification, item development, two rounds of voting, and a meeting to finalise the consensus statements. The pre-defined threshold for agreement was set at ≥70%.

Setting: Specialist outpatient services.

Participants: Members of the UK Head Injury Network were invited to participate. The network consists of clinical specialists in head injury practising in emergency medicine, neurology, neuropsychology, neurosurgery, paediatric medicine, rehabilitation medicine and sports and exercise medicine in England.

Primary outcome measure: A consensus statement on the structure and process of specialist outpatient care for concussion patients in England.

Results: 56 items were voted on in the first round. 30 items were removed following the first voting round and 3 items were removed following the second voting round. Items were modified where appropriate. A final 18 statements reached consensus covering three main topics in specialist healthcare services for concussion; care pathway to structured follow-up, prognosis and measures of recovery, and provision of outpatient clinics.

Conclusions: This work presents statements on how the healthcare services for concussion patients in England could be redesigned to meet their health needs. Future work will seek to implement these into the clinical pathway.

For peer review only - http://bmjopen.bmj.com/site/about/guidelines.xhtml
For peer review only

Strengths and limitations of this study

- This consensus statement outlines structural and process-based factors needed to move towards standardised best-practice care for concussions in England to improve healthcare quality and patient outcomes
- To our knowledge, no studies have reported on the healthcare system for concussion in England
- We engaged with various stakeholders involved in concussion care to ensure different perspectives are represented
- It cannot be excluded that some disagreement on statements was in part due to differences in definitions and terminology use despite probable broad agreement on the statement in principle.
- The statements reached agreement in this panel, but we cannot exclude the possibility or to what degree the results are influenced by the experience and expertise of these individuals both for included and excluded statements.

For peer review only

INTRODUCTION

In England and Wales alone, more than a million people suffer a head injury every year (1). These injuries occur as a result of direct or indirect impacts to the head and are commonly encountered in sports, both at professional and grassroots levels. Injuries to the head, particularly in the context of sport, are often labelled "concussions". While there is a recently published definitions available (2), there is no widespread consensus for what constitutes a concussion, and it is often used to describe both the symptoms suffered following a head injury as well as the pathophysiological mechanisms causing these symptoms (3,4). Despite some controversies in the field, concussion is widely recognised by the public and those playing sport, and for this reason, we have used the term for the purpose of this paper. A concussion is diagnosed on clinical grounds with evidence of immediate and transient neurological dysfunction that may or may not present with a wide range of signs and symptoms, such as loss of consciousness, post-traumatic amnesia, headache and vomiting (5). However, most concussions do not present with obvious neurological signs, such as loss of consciousness, making recognition and diagnosis challenging (6–9).

The post-concussion sequelae are assumed to recover spontaneously within a few days to a couple of weeks. Yet a proportion of individuals experience persistent symptoms for months to years after injury that requires timely and appropriate medical assessment and treatment (10). Despite this known fact, guidance on clinical practice in the outpatient setting after the initial medial review is limited and ambiguous leaving the concussion population vulnerable to variations and sub-optimal care (11). This results in delayed intervention, resource waste, care inequalities and poorer quality of care (12–14). There is no standardised, structured follow-up for those who experience persistent symptoms and current clinical services largely fail to meet the healthcare needs of these patients (15).

Nonetheless, the past two decades have seen an exponential growth in concussion research which has improved our understanding of the injury and strategies to enhance recovery and quality of care (16). Additionally, there is increasingly more awareness around the potential lasting effects of concussions, especially in sports, where there are growing concerns and calls for action (17). The government and sports' national governing bodies are attempting to improve concussion management in all levels of sports by standardising protocols, allocating more funds for concussion research and incorporating new playing rules to reduce concussion risk and improve management (18). Consequently, the demand for care is evolving and to

For peer review only - http://bmjopen.bmj.com/site/about/guidelines.xhtml

meet patient needs changes to clinical services are required to provide evidence-based best-practice care whilst being cost-effective, delivering better healthcare value and quality and improving health outcome for concussion patients (7,19). This necessitates fundamental changes to the healthcare system in concussion care to facilitate the delivery of best-practice outpatient care and improve quality of care (20). This consensus statement addresses the healthcare structure and processes in outpatient concussion care required to facilitate the delivery of best-practice care in specialist outpatient clinics to meet healthcare needs and improve patient outcome in the concussion population.

METHODS

The guidance on Conducting and REporting DElphi Studies was followed for reporting this consensus study (21).

The work was undertaken as part of wider work by the UK Head Injury Network. There was no direct involvement with patients or the public. The study did not meet the criteria for requiring ethical approval.

Study design

A modified Delphi approach was used for this study. We followed a five-stage process; (1) panel identification, (2) literature search for initial statement development, (3-4) two voting rounds followed by item removal and modifications and (5) consensus meeting and live voting to finalise the statements. The meeting was chaired by a working group member with previous experience in the Delphi consensus method.

Panel Selection

The UK Head Injury Network is a panel of clinical and academic experts with extensive experience in head injury care in England, and setup to improve access to and quality of concussion outpatient services – for sports-related and non-sports-related concussions – and provide a network to foster research collaborations. The network consists of a multidisciplinary panel from academia and clinical medicine with specialists in emergency medicine, neurology, neuropsychology, neurosurgery, neuroscience, paediatric medicine, rehabilitation medicine and sports and exercise medicine. Members are invited to join the UK

For peer review only - http://bmjopen.bmj.com/site/about/guidelines.xhtml

For peer review only

Head Injury Network if they meet the following criteria; (1) healthcare professionals with extensive first-hand clinical experience in concussions care in any care setting or specialty; and/or (2) academic expertise in concussions. The group work with Love of the Game, a national sports concussion charity, to achieve these goals.

All members of the UK Head Injury Network at the time of the consensus process were invited to vote and attend the final consensus meeting. The participating members were geographically diverse including representatives from a range of healthcare settings across England. In addition to the various specialties involved, this ensures a more representative and informed decision-making for the different stages and settings of care across England.

For peer review only

Evidence Review

The need for a consensus statement had been discussed in previous meetings and highlighted in work completed by the group (unpublished observations). As the field of concussion has several contentious issues, we took a wide approach to topics covered in the initial statement development.

As the literature on implementation science in concussion is scarce, the literature search provided the evidence for clinical tasks that could be implemented. The panel used this literature combined with their first-hand local experience in various clinical settings to provide guidance on the structures and processes required to feasibly deliver these clinical tasks in England. Thus, statement development was guided by a literature search, topics of discussion within the network and results from previous work. MEDLINE and Embase were searched for relevant literature in October 2022, primarily published national and international clinical guidelines and consensus statements (see supplementary material 1 for search strategy). The search was limited to literature published in English. Systematic reviews and randomised controlled trials were used to guide expert opinion to determine whether there was sufficient evidence for implementation into nation-wide clinical practice in England.

A subgroup of network members was responsible for this stage of the process (EK, PJH, AH). The initial statements were circulated to all network members for comments on the scope, structure and wording prior to starting the official rounds of voting.

For peer review only - http://bmjopen.bmj.com/site/about/guidelines.xhtml

Consensus Process

Two rounds of online surveys were administered between October and December 2022 followed by a virtual meeting in February 2023. The surveys were distributed for voting using the Qualtrics XM platform (Qualtrics, Provo, UT).

All voting was anonymous to ensure uninhibited voting. Participants were asked to vote on each statement and had the binary options agree-disagree. The cut-off threshold for agreement was set at ≥70% for the two rounds of voting (22–24). A statement was only included if agreement was above the pre-defined threshold for consensus. Free text comments were incorporated to allow members to suggest modifications or refinements of the statements. Statements that did not meet the threshold were excluded after each round of voting and statements were modified for further considerations if suggestions were made by the participants.

All participants were invited to a virtual meeting (Zoom Video Communications, San Jose, USA). The meeting was schedules for 3 hours to provide sufficient time to discuss each statement in detail. The purpose of the meeting was to finalise the statements. Statements reaching agreement and disagreement were discussed where alternatives were considered for the latter kind. The threshold for agreement was raised to ≥85% for this meeting to ensure a robust degree of consensus. The final consensus statements were discussed and, where necessary, modifications were made to ensure clarity and accuracy before a final anonymous vote took place during the meeting.

Patient and Public Involvement

No patient involved.

RESULTS

Evidence Review

Whilst several papers were identified on therapeutic interventions, educational interventions, and biomarkers for diagnosis and prognosis in concussion, as expected the literature on healthcare processes and structures in concussion is scarce. Thus, the final statements were

For peer review only - http://bmjopen.bmj.com/site/about/guidelines.xhtml

primarily guided by previous discussions, interpretation of how research evidence on best-practice could be formulated into the structure and process of the healthcare system, and research work by the network and patient focus group sessions (unpublished observation).

Key papers identified during the literature review included clinical practical guidelines on concussion management (25–28) and systematic reviews on therapeutic interventions (29–34), prognosis (35–44), recovery (45,46) and biomarkers (47,48). The clinical practice guidelines included a synthesis of practical guidelines primarily focused on four published papers; Center for Disease Control and Prevention Guideline on the Diagnosis and Management of Mild Traumatic Brain Injury Among Children (2019) (49), Consensus Statement on Concussion in Sport – the 5th International Conference on Concussion in Sport (2016), Ontario Neurotrauma Foundation Guideline for Concussion/Mild Traumatic Brain Injury & Persistent Symptoms (3rd edition; 2018), and Department of Veterans Affairs/Department of Defense Clinical Practice Guideline for the Management of Concussion-Mild Traumatic Brain Injury (2016) (26).

Whilst it was agreed that covering a wide range of topics was appropriate during the initial statement development, the statements in this consensus report were narrowed down to specifically focus on the delivery of healthcare provisions through processes and structures in concussion care. This was deemed to be the most appropriate and relevant due to (a) some disagreement on clinical practice and management issues in concussion among the panel, (b) determining that these statements were beyond the purpose of this group and some of this guidance is featured in the most recent international *Consensus Statement on Concussion in Sport (6th edition)* (50), and (c) recognition that the need for the healthcare service to be improved at a system level prior to changes in day-to-day clinical practice. These points became clear after the first round of voting and the majority of these statement were removed after this stage. The decision to remove the remaining statements on day-to-day practice was made during the final meeting.

Consensus Process

Table 1: Demographics for participating panel members

**Characteristics**	**n (%)**

For peer review only - http://bmjopen.bmj.com/site/about/guidelines.xhtml

Sex, female	6 (21%)
**Specialty**	
Emergency medicine	2 (7%)
Neurology	3 (11%)
Neuropsychiatry	1 (4%)
Neuropsychology	3 (11%)
Neurosurgery	12 (43%)
Paediatric medicine	1 (4%)
Rehabilitation medicine	1 (4%)
Neuroscience	2 (7%)
Sports and exercise medicine	2 (7%)
Other	1 (4%)
**Region**	
East Midlands	1 (4%)
East of England	5 (18%)
Greater London	3 (11%)
North East England	4 (14%)
North West England	5 (18%)
South East England	4 (14%)
South West England	3 (11%)
West Midlands	1 (4%)
Yorkshire and the Humber	2 (7%)

Both rounds of voting had 21 votes (75% of network members at the time; see table 1 for demographic information on participating members). Following the initial statement

For peer review only - http://bmjopen.bmj.com/site/about/guidelines.xhtml

development, the first round of voting had 56 items (figure 1, supplementary material 2.1). 9 statements were eliminated after the first round of voting as they were below the threshold of agreement and 6 statements were amended based on comments from voting members. Of the 56 statements included in the first round of voting, 23 statements were included that did not follow the standard consensus methodology (supplementary material 2.2). A different process was used for these statements and contained single-select or multi-select multiple choice options rather than the binary agree-disagree format used for the consensus statements. These statements are an important part of understanding the structure and processes for service delivery. Based on the responses from the first round of voting, two of these statements were amended and added as consensus statements with the binary agree-disagree response options in the following round of voting. As a result, the second round of voting included 26 statements that were put to a vote (supplementary material 3). Three items did not reach the threshold of agreement following the second round of voting and were removed, leaving 23 statements to be discussed during the meeting. No statements were amended at this stage.

The virtual consensus meeting resulted in four statements being eliminated, two pairs of statements were combined and one statement was added. Of the eliminated statements, three were deemed to be beyond the scope of this consensus statement. The statement added during the meeting was suggested by one member and voted on twice as the level of agreement was just below the threshold following the first vote. The statement was modified and included following the second vote as the level of agreement was above the threshold.

Figure 1: Consensus process

This resulted in a final 18 statements (table 2), all above the raised threshold of 85% agreement. 14 statements achieved unanimous agreement. The final statements were organised into three themes; (1) care pathway to structured follow-up, (2) prognosis and measures of recovery, and (3) provision of outpatient clinics.

Table 2: Final consensus statements

	Agreement %
**1 Care Pathway to Structured Follow-up**	

For peer review only - http://bmjopen.bmj.com/site/about/guidelines.xhtml

Concussion patients with persistent symptoms should be able to access specialist clinics.	100%
There should be dedicated concussion clinics, or where not possible, clinics with dedicated time for concussion patients.	100%
Specialist head injury clinics should comprise a multidisciplinary team that should include, as a minimum, a clinician, neuropsychologist and vestibular physiotherapist.	100%
There should be a direct patient pathway for patients with persistent symptoms to specialist outpatient services from the ED, GP and other sources.	100%
**2 Prognosis and Measures of Recovery**	
There should be a system for patients to be triaged to a specialist in head injury clinics based on persistent symptoms.	100%
There may be a role for digital tools (e.g., platforms, apps) to be used for screening for persistent symptoms following concussion.	100%
Symptom scales can aid the assessment of symptom burden.	100%
Screening for persistent symptoms can be aided by symptom scales.	100%
A new patient-reported tool for the assessment of symptoms and outcome should be developed and validated.	90%
Prolonged symptoms may develop even in the absence of specific acute signs, such as loss of consciousness, post-traumatic amnesia and vomiting.	100%

For peer review only - http://bmjopen.bmj.com/site/about/guidelines.xhtml

There are no prognostic tools currently available with sufficient clinical accuracy to predict the risk of developing persistent symptoms.	100%
There is currently insufficient evidence to support the clinical application of objective biomarkers (i.e., fluid, imaging) for identifying and screening for persistent symptoms after a concussion.	100%
Symptomatic patients with abnormal cranial/brain imaging should be considered for follow-up in a specialist clinic.	89%
There is not yet sufficient evidence to support the routine use of advanced imaging techniques (e.g., DTI, fMRI, MEG) in clinical practice.	90%
**Provision of Outpatient Clinics**	
All patients should be directed to online information on concussion to help with expectations and recommendations.	100%
All clinics should have access to standardised protocols for assessing pituitary dysfunction.	100%
Co-morbidities, socioeconomic status and social support should be factored into clinical decision-making on management.	90%
The underlying cause(s) of persistent symptoms should be established and treated on a case-by-case basis.	100%

DTI, Diffusion Tensor Imaging; ED, Emergency Department; fMRI, functional Magnetic Resonance Imaging; GP, General Practice; MEG, Magnetoencephalography

DISCUSSION

Healthcare services for concussion patients need to be transformed to advance quality of care and patient outcome. Current healthcare services fail to meet the needs of a significant number of concussion patients which results in suboptimal recovery and failure of the

For peer review only - http://bmjopen.bmj.com/site/about/guidelines.xhtml

healthcare system to fulfil its purpose of improving quality of life through enhancing health. There is an urgent need to improve the delivery of and access to concussion care in order to reduce healthcare inequalities and errors in management. Guidelines exist, but healthcare services need to restructure so to facilitate the delivery of these guidelines.

The recommendations in this consensus statement primarily focus on the structure and process of specialist healthcare services in concussion care with the purpose of improving healthcare quality and patient outcome (20). These are the components in healthcare services that facilitate the implementation and delivery of best-practice care to achieve optimal health for the target patient population (51). Ultimately, patient outcome is determined by the structure and process of care which describe the care context and the sum of actions in the delivery of care, respectively (52). This consensus statement prioritises a patient-centric approach with a focus on timely, integrated, effective, efficient and evidence-based care and urge standardisation of these practices nationally.

Given the recent developments in the field, translating basic and clinical evidence into the healthcare system requires work on feasibility, implementation and service redesign (53,54). Without a systematic approach, the promise and desired goal of improving patient outcomes can be challenging to fulfill. Simultaneously, there are still gaps in evidence. Consensus statements serve as an important tool in bridging areas where evidence is under-developed (55). This consensus statement provides guidance outlines how to redesign the structures and processes in care delivery for concussion patients in England. This is through expert opinion based on the current level of evidence and first-hand local experience in the various clinical settings which concussion patients encounter and academic expertise. The guidance needs to be updated as the evidence changes, but this ensures patient outcome to improve without delay.

The non-consensus statements included in the first voting round will help guide system redesign in future work through providing further detailed guidance on fundamental factors in concussion healthcare delivery. This includes the structure of clinical teams, timelines for care tasks and other processes in the clinical pathway from injury to specialist outpatient services.

For peer review only - http://bmjopen.bmj.com/site/about/guidelines.xhtml

Whilst the consensus statements are aimed at all concussions, sports-related and non-sports-related concussions, the implementation of these services into the National Health Services in England requires reallocation and reprioritisation of resources, as well as a restructuring of the system. Applying these changes for the subgroup of concussions occurring in sports – at both professional and grassroot levels – could be more practical and achievable at this stage, especially accounting for the high number of all-cause concussions. For example, direct patient pathways from local sports clubs could feasibly be implemented with quick-access clinics given the manageable number of sports-related concussions. A short-term goal of the UK Head Injury Network is the development of regional sports concussion clinics across the country to alleviate the burden of concussions in all levels of sports. These clinics aim to provide evidence-based care. However, it is intended that these services will ultimately be expanded and offered to all-cause concussion patients. Thus, while the implementation of these statements will primarily change clinical services for sports-related concussions in the near future, the statements are directed at national health services offered to all patients in the longer term.

Clinical Implications

Care Pathway to structured follow-up

Patients with persistent symptoms require further medical attention following the initial medical review– which is typically performed in emergency departments, general practice surgeries or by sport physicians – to recover to their pre-injury level of health and function. Yet, in a CENTER-TBI study, 90% of 71 neurotrauma centres involved in the Europe-based study do not routinely schedule follow-up appointments for head injury patients discharged from the emergency department (56).

In the UK, there is no care pathway to ensure access to structured follow-up which is likely a fundamental underlying cause for the unmet needs in concussion care. The absence of such a service may be attributed to the heterogeneity of concussions and the vast differences in patient needs. However, a flexible and multidisciplinary approach is needed to personalise care and meet patient needs across the heterogenous recovery trajectories in the concussion population. Evidence suggests that providing multidisciplinary interventions can improve return to work and quality of life in this patient population (57). Appropriate resources – time, equipment and staffing – need to be allocated to these services to ensure there is

For peer review only - http://bmjopen.bmj.com/site/about/guidelines.xhtml

capacity to meet the diversity and scale of need. As a minimum, we suggest the inclusion of, though not limited to, a clinician (i.e. specialist nurse, neurologist, neurosurgeon, sport and exercise physician), neuropsychologist and vestibular physiotherapist. This is due to the incidence of neurological, cognitive, psychological, cervicogenic and vestibular symptoms in post-concussive patients (58–60). Moreover, due to the knowledge and skillset of physiotherapists, it may be beneficial to develop a new subspecialty in 'concussion physiotherapy' (28,61).

Providing timely care is fundamental to meet patients' health needs. Delaying intervention can prolong recovery further and result in poorer outcome. One study found patients who sought care more than 30 days after a concussion had worse outcome at three months compared with patients who sought care earlier (62). Concussion services should be redesigned with care pathways that offer access to structured follow-up for all concussion patients as necessary to ensure these patients receive timely and appropriate care. These statements echo the recommendations in the recent Lancet Commission on traumatic brain injury (63).

Prognosis and Measures of Recovery

Objective measures of disease and prognostic factors provide a means for directing care (64). One of the greatest challenges in concussion management is the lack of objective markers and the absence of reliable prognostic factors for recovery. Thus, early identification of patients at risk of prolonged recovery is not possible and therefore assessing the need for follow-up for individuals at an early stage is challenging. Consequently, no predictive tool can be used reliably in the clinical setting to assess the risk of prolonged recovery and the need for follow-up in the outpatient setting (40). Even patients without marked acute signs and symptoms are at risk of prolonged recovery and should not be dismissed inadvertently. Of note, the panel recommends that patients with abnormal scans during the acute phase should always be offered follow-up as there is significant observable pathology during the acute phase to warrant further medical assessment (65). There is evidence to suggest that some gross structural abnormalities found on acute CT imaging may be associated with worse outcomes (66). However, we suggest that anything beyond standard structural imaging modalities – to rule out other pathologies – should not be used in the clinical setting. Whilst advanced imaging methods are showing promise in research, they are yet to demonstrate

For peer review only - http://bmjopen.bmj.com/site/about/guidelines.xhtml

reliable clinical utility and we do not recommend using these tools in clinical practice (38,47,48).

In the absence of validated objective measures, ongoing self-reporting is currently the most reliable primary indicator of health status and method for identification of patients with persistent symptoms (67). Of note, non-disclosure of concussion symptoms is a concern that needs to be addressed and is particularly prevalent in professional and community sports (68,69). To filter out people who have recovered and identify individuals who need further medical review, we recommend a screening system. This would need to be cost-effective and scalable due to the size of the concussion population. We suggest digital tools as a platform for the screening system as they offer a remote, convenient, efficient and scalable solution (70). Once set up, costs remain comparatively low whilst also overcoming traditional barriers related to geographical, financial and resource limitations (71,72). Such a system could also be efficiently and effectively used to triage patients to the most appropriate clinician in the multidisciplinary clinic based on patient-reported symptoms and disabilities.

Standardising evaluation of recovery in the absence of objective markers is challenging. However, symptom scales may aid assessment of symptom burden and provide a means for screening patients, particularly as history taking and patient-reported symptoms are currently the only methods for measuring recovery (67). To achieve this long-term, we recommend developing and validating a patient-reported outcome measure for the concussion population to help assess recovery and determine care needs. This may include assessment of symptom burden, functional status, return to work or activity and quality of life. However, the specifics need to be determined in an appropriate study. While this study focuses on clinically-actionable statements, we believe this is a research priority that is important to include as it is required to achieve many of the clinical statements recommended. A validated patient-reported outcome measure will facilitate the clinical pathway for concussion patients to specialist outpatient services and will help move the field forward.

In the era of patient-centric and value-based healthcare, patient-reported outcome measures are increasingly important to capture the patient’s perspective of their health. Even when objective measures are identified in the future, these tools should be utilised to capture a holistic patient-centric assessment of health status and recovery.

For peer review only - http://bmjopen.bmj.com/site/about/guidelines.xhtml

Provision of Outpatient Clinics

Providing early information and awareness is essential to managing expectations and can enhance recovery. A TRACK-TBI study found that only 42% of patients reported receiving head injury information at discharge from the emergency department (73). Providing proper discharge information can be challenging in the emergency department due to the busy environment in these clinical settings and the cognitive state of the patients initially after injury which may prevent them from comprehending and retaining information (74). Thus, we suggest directing patients to a single online platform for evidence-based information. The online modality allows anyone to access the information at any time, provides the same information to all regardless of geographical region and is easier to keep up to date with the evidence, which is particularly important in a fast-evolving field.

For patients with persistent symptoms, determining the most appropriate therapy can be challenging, especially in the absence of sufficiently strong evidence on therapeutic efficacy and timing of interventions. Given the vast heterogeneity in cases, no single treatment plan can be utilised for all concussion patients and clinicians must evaluate the most appropriate intervention(s) on a case-by-case basis. Whilst further research is required in this domain some therapeutic interventions have shown promise. Recommendations for therapeutic approaches are beyond the scope of this consensus statement and we recommend referring to other literature on this topic (29–34). Biopsychosocial factors should be considered when assessing clinical and social care needs as these have been shown consistently to influence patient recovery (41).

Lastly, we suggest that concussion clinics should have access to a standardised protocol for assessing pituitary function. Pituitary dysfunction can result in a range of different symptoms, many of which overlap with concussion symptoms, including fatigue, anxiety and mood disturbances. The literature on the incidence of pituitary dysfunction after concussions and screening protocols is still an area of active research. We suggest referring to a guidance paper by the British Neurotrauma Group on screening and management of pituitary dysfunction after traumatic brain injury (75).

Recent Developments in Sports Concussion Guidelines

For peer review only - http://bmjopen.bmj.com/site/about/guidelines.xhtml

The 6th edition of the consensus statement on concussion in sport were published this year (50). This statement also advises that individuals with persistent symptoms after a concussion (suggested as symptom persisting for longer than 4 weeks) should undergo a multimodal evaluation, including the use of standardised, validated symptom scales for assessment and to guide referrals. The statement recommends referral to specialist clinicians ideally part of a multidisciplinary network of practitioners, where possible, for targeted treatment for persistent symptoms including *cervicogenic symptoms, migraine and headache, cognitive and psychological difficulties, balance disturbances, vestibular signs and oculomotor manifestations*. Regarding the multidisciplinary network, the statement proposes the inclusion of *sports medicine physicians, athletic trainers/therapists, physiotherapists, occupational therapists, sports chiropractors, neurologists, neurosurgeons, neuropsychologists, ophthalmologists, optometrists, physiatrists, psychologists and psychiatrists.* Furthermore, biopsychosocial and co-morbidities should also be considered in the context of persistent symptoms after concussions. Additionally, similar guidance has been provided regarding the use of advanced neuroimaging and other objective biomarkers in clinical practice.

Limitations

The literature on concussion, though rapidly evolving, is contentious and there are still numerous areas of disagreement amongst the panel. As discussed above, a wide approach was taken for the initial statement development which was later narrowed to focus on the healthcare structure and processes, in part. It cannot be excluded that some disagreement on statements was in part due to differences in definitions and terminology use despite probable broad agreement on the statement in principle. However, establishing agreement on terminology is a different consensus process on its own and was beyond the scope of this study.

We engaged with different stakeholders including academics, clinicians from different specialties and charities, and drew from previous patient-focus group sessions (unpublished observations). To ensure more robust statements, we chose a high threshold for consensus. Whilst these statements reached agreement in this panel, we cannot exclude the possibility or to what degree the results are influenced by the experience and expertise of these individuals both for the included and excluded statements. The number of participants was in line with recommendations for Delphi studies (76), however, more representatives from each specialty

For peer review only - http://bmjopen.bmj.com/site/about/guidelines.xhtml

involved could have benefitted the consensus process (77). However, due to the high level of agreement and consistency with the literature, we believe the statements can be integrated reliably in the national healthcare system.

General practitioners are typically the first point of contact for patients with persistent problems seeking medical attention after a concussion and are a key player in the patient pathway. While the perspective of general practitioners is crucial for the wider concussion healthcare services, this study focused on the structures and processes in specialist outpatient services. However, the involvement of general practitioners is crucial for implementation and redesigning the concussion pathway.

CONCLUSION

This consensus statement outlines structural and process-based factors needed to move towards standardised best-practice care in concussion. This includes a final 18 statements on the care pathway for structured follow-up, prognosis and measures of recovery and provision of outpatient clinics. The statements are aimed at both the sports-related and all-cause concussion populations, however, they should be implemented for sports-related concussions as the first step before expanding to all concussion patients given the greater feasibility of this approach. Further research and stakeholder engagement is needed to determine best-practice clinical concussion management in outpatient clinics and the recommendations in this statement provide the structures and processes whereby best-practice patient-centred care can be delivered in a timely, integrated, efficient and effective manner.

Acknowledgements

We would like to thank Love of the Game for their support with this work. Peter Hutchinson would also like to thank the NIHR Senior Investigator Award, Cambridge Biomedical Research Centre, Medtech Brain Injury Co-operative, Global Health Research Group on Acquired Brain and Spine Injury and Royal College of Surgeons of England. AH is supported by the Cambridge Biomedical Research Campus, the Royal College of Surgeons of England, and is a Theme Lead in the NIHR Brain Injury MedTech Collaborative.

Contributorship statement

All authors have made substantial contributions to the manuscript in the following ways: EK, AH, AK, MP and PJAH led the delivery and planning of this consensus statement. AB, CG,

For peer review only - http://bmjopen.bmj.com/site/about/guidelines.xhtml

MGre, MGri, TG, PG, AH, DH, PJAH, PJ, BJ, AK, EK, TL, TM, CH, SM, JN, RN, VR, KS, RS, DT, PW, MW, EW were responsible for the conception and design of the study. AK moderated the consensus meeting. AB, CG, MGre, MGri, TG, PG, AH, DH, PJAH, PJ, BJ, AK, EK, TL, TM, CH, SM, JN, RN, VR, KS, RS, DT, PW, MW, EW participated in the acquisition, analysis and interpretation of data. EK drafted the manuscript and CG, MGa, TG, AH, PJAH, PJ, BJ, AK, EK, CH, RN, DT, PW, and EW revised it thoroughly. All authors gave final approval of the submitted manuscript version.

Competing interests

AB serves as an expert advisor on concussion for England's Rugby Football Union, Football Association and Premier League. He also consults and has stocks in Marker Diagnostics LTD and licenses in Salivary Biomarker of Concussion. AH served on the NICE Guideline Committee Trauma Guidelines for 2023. BJ consults for Premiership Rugby, London, and Rugby Football League, Manchester. DT consults for the Leeds Rhinos Rugby League Club and Hull City Tigers Football Club. MG serves on the advisory board for the Players Football Association. RN has undertaken work as a medicolegal expert in sports concussions.
RS has served as an independent expert in legal cases in which head injury has occurred, some of these were mild head injuries. He also has received funding from the Rugby Football Association and Football Association for the Advanced Brainhealth project. TG has advised on legal cases where head injury has occurred, some of which were mild head injuries.

Funding

No funding was received for this work.

Data availability statement

Data are available upon reasonable request.

Ethical statement

The study was exempt from requiring ethical approval in accordance with the University of Cambridge Policy on the Ethics of Research Involving Human Participants and Personal Data.

REFERENCES

For peer review only

1. National Institute for Health and Care Excellence. Head injury: assessment and early management [online]. [London]: NICE; 2023 [cited 2023 October 5]. (NICE guideline [NG232]). Available from https://www.nice.org.uk/guidance/ng232
2. Silverberg ND, Iverson GL, Cogan A, Dams-O-Connor K, Delmonico R, Graf MJP, et al. The American Congress of Rehabilitation Medicine Diagnostic Criteria for Mild Traumatic Brain Injury. Arch Phys Med Rehabil. 2023 Aug;104(8):1343–55.
3. Sharp DJ, Jenkins PO. Concussion is confusing us all. Pract Neurol [Internet]. 2015;15(3):172–86. Available from: https://www.scopus.com/inward/record.uri?eid=2-s2.0-84929330344&doi=10.1136%2fpractneurol-2015-001087&partnerID=40&md5=c2ae641c33158dcbe06dc0b311be793f
4. Ganau L, Ligarotti GKI, Prisco L, Ganau M. Letter: A Scoping Review of Registered Clinical Studies on Mild Traumatic Brain Injury and Concussion (2000-2019). Neurosurgery. 2021 Sep;89(3):E178–9.
5. Levin HS, Diaz-Arrastia RR. Diagnosis, prognosis, and clinical management of mild traumatic brain injury. Lancet Neurol. 2015 May;14(5):506–17.
6. Marshall SW, Guskiewicz KM, Shankar V, McCrea M, Cantu RC. Epidemiology of sports-related concussion in seven US high school and collegiate sports. Inj Epidemiol. 2015;2(1).
7. Langer L, Levy C, Bayley M. Increasing Incidence of Concussion: True Epidemic or Better Recognition? Journal of Head Trauma Rehabilitation. 2020 Jan;35(1):E60–6.
8. McCrea M, Guskiewicz KM, Marshall SW, Barr W, Randolph C, Cantu RC, et al. Acute Effects and Recovery Time Following Concussion in Collegiate Football Players. JAMA. 2003 Nov 19;290(19):2556.
9. McCrory P, Meeuwisse WH, Echemendia RJ, Iverson GL, Dvořák J, Kutcher JS. What is the lowest threshold to make a diagnosis of concussion? Br J Sports Med. 2013 Apr 11;47(5):268–71.
10. Cancelliere C, Verville L, Stubbs JL, Yu H, Hincapié CA, Cassidy JD, et al. Post-Concussion Symptoms and Disability in Adults With Mild Traumatic Brain Injury: A Systematic Review and Meta-Analysis. Vol. 40, Journal of Neurotrauma. 2023.
11. Stern RA, Seichepine D, Tschoe C, Fritts NG, Alosco ML, Berkowitz O, et al. Concussion Care Practices and Utilization of Evidence-Based Guidelines in the Evaluation and Management of Concussion: A Survey of New England Emergency Departments. Vol. 34, Journal of Neurotrauma. 2017.

For peer review only - http://bmjopen.bmj.com/site/about/guidelines.xhtml

12. Ross J, Santhirapala R, MacEwen C, Coulter A. Helping patients choose wisely. Vol. 361, BMJ (Clinical research ed.). 2018.
13. Maughan D, Ansell J. Protecting resources, promoting value: a doctors guide to cutting waste in clinical care. Vol. 24, Academy of Medical Royal Colleges. 2014.
14. Williams E, Buck D, Babalola G. What are health inequalities? [online]. [London]: The King's Fund; 2020 [updated 2022 June; cited 2023 Oct 5]. Available from https://www.kingsfund.org.uk/publications/what-are-health-inequalities
15. Dasic D, Morgan L, Panezai A, Syrmos N, Ligarotti GKI, Zaed I, et al. A scoping review on the challenges, improvement programs, and relevant output metrics for neurotrauma services in major trauma centers. Surg Neurol Int. 2022 Apr 29;13:171.
16. National Institute for Health and Excellence. Practical steps to improving the quality of care and services using NICE guidance [Internet]. 2018 [cited 2023 Oct 4]. Available from: https://www.nice.org.uk/Media/Default/About/what-we-do/Into-practice/Practical-steps-improving-quality-of-care-and-services-using-NICE-guidance.pdf
17. Wiebe DJ, Comstock RD, Nance ML. Concussion research: a public health priority. Injury Prevention. 2011 Feb 1;17(1):69–70.
18. Department for Culture M and SUG. UK Concussion Guidelines for Non-Elite (Grassroots) Sport [Internet]. 2023 [cited 2023 Sep 25]. Available from: http://sramedia.s3.amazonaws.com/media/documents/9ced1e1a-5d3b-4871-9209-bff4b2575b46.pdf
19. Veliz P, McCabe SE, Eckner JT, Schulenberg JE. Trends in the Prevalence of Concussion Reported by US Adolescents, 2016-2020. JAMA. 2021 May 4;325(17):1789.
20. Hannawa AF, Wu AW, Kolyada A, Potemkina A, Donaldson LJ. The aspects of healthcare quality that are important to health professionals and patients: A qualitative study. Patient Educ Couns. 2022;105(6).
21. Jünger S, Payne SA, Brine J, Radbruch L, Brearley SG. Guidance on Conducting and REporting DElphi Studies (CREDES) in palliative care: Recommendations based on a methodological systematic review. Vol. 31, Palliative Medicine. 2017.
22. Diamond IR, Grant RC, Feldman BM, Pencharz PB, Ling SC, Moore AM, et al. Defining consensus: A systematic review recommends methodologic criteria for reporting of Delphi studies. J Clin Epidemiol. 2014 Apr;67(4):401–9.

For peer review only - http://bmjopen.bmj.com/site/about/guidelines.xhtml

23. Slade SC, Dionne CE, Underwood M, Buchbinder R. Standardised method for reporting exercise programmes: protocol for a modified Delphi study. BMJ Open. 2014 Dec 30;4(12):e006682.

24. Vogel C, Zwolinsky S, Griffiths C, Hobbs M, Henderson E, Wilkins E. A Delphi study to build consensus on the definition and use of big data in obesity research. Int J Obes. 2019 Dec 17;43(12):2573–86.

25. Giza CC, Kutcher JS, Ashwal S, Barth J, Getchius TSD, Gioia GA, et al. Summary of evidence-based guideline update: Evaluation and management of concussion in sports: Report of the Guideline Development Subcommittee of the American Academy of Neurology. Neurology. 2013 Jun 11;80(24):2250–7.

26. Silverberg ND, Iaccarino MA, Panenka WJ, Iverson GL, McCulloch KL, Dams-O'Connor K, et al. Management of Concussion and Mild Traumatic Brain Injury: A Synthesis of Practice Guidelines. Arch Phys Med Rehabil. 2020 Feb;101(2):382–93.

27. Marshall S, Bayley M, McCullagh S, Velikonja D, Berrigan L, Ouchterlony D, et al. Updated clinical practice guidelines for concussion/mild traumatic brain injury and persistent symptoms. Brain Inj. 2015 May 12;29(6):688–700.

28. Quatman-Yates CC, Hunter-Giordano A, Shimamura KK, Landel R, Alsalaheen BA, Hanke TA, et al. Physical Therapy Evaluation and Treatment After Concussion/Mild Traumatic Brain Injury. Journal of Orthopaedic & Sports Physical Therapy. 2020 Apr;50(4):CPG1–73.

29. Boussard CN de, Holm LW, Cancelliere C, Godbolt AK, Boyle E, Stålnacke BM, et al. Nonsurgical Interventions After Mild Traumatic Brain Injury: A Systematic Review. Results of the International Collaboration on Mild Traumatic Brain Injury Prognosis. Arch Phys Med Rehabil. 2014 Mar;95(3):S257–64.

30. Schneider KJ, Leddy JJ, Guskiewicz KM, Seifert T, McCrea M, Silverberg ND, et al. Rest and treatment/rehabilitation following sport-related concussion: a systematic review. Br J Sports Med. 2017 Jun;51(12):930–4.

31. Murray DA, Meldrum D, Lennon O. Can vestibular rehabilitation exercises help patients with concussion? A systematic review of efficacy, prescription and progression patterns. Br J Sports Med. 2017 Mar;51(5):442–51.

32. Lal A, Kolakowsky-Hayner SA, Ghajar J, Balamane M. The Effect of Physical Exercise After a Concussion: A Systematic Review and Meta-analysis. Am J Sports Med. 2018 Mar 1;46(3):743–52.

For peer review only - http://bmjopen.bmj.com/site/about/guidelines.xhtml

33. Reid SA, Farbenblum J, McLeod S. Do physical interventions improve outcomes following concussion: a systematic review and meta-analysis? Br J Sports Med. 2022 Mar;56(5):292–8.

34. Heslot C, Azouvi P, Perdrieau V, Granger A, Lefèvre-Dognin C, Cogné M. A Systematic Review of Treatments of Post-Concussion Symptoms. J Clin Med. 2022 Oct 21;11(20):6224.

35. Rausa VC, Anderson V, Babl FE, Takagi M. Predicting Concussion Recovery in Children and Adolescents in the Emergency Department. Curr Neurol Neurosci Rep. 2018 Nov 19;18(11):78.

36. Mercier E, Tardif PA, Cameron PA, Émond M, Moore L, Mitra B, et al. Prognostic value of neuron-specific enolase (NSE) for prediction of post-concussion symptoms following a mild traumatic brain injury: a systematic review. Brain Inj. 2018 Jan 2;32(1):29–40.

37. Zetterberg H, Morris HR, Hardy J, Blennow K. Update on fluid biomarkers for concussion. Concussion. 2016 Dec;1(3):CNC12.

38. McCrea M, Meier T, Huber D, Ptito A, Bigler E, Debert CT, et al. Role of advanced neuroimaging, fluid biomarkers and genetic testing in the assessment of sport-related concussion: a systematic review. Br J Sports Med. 2017 Jun;51(12):919–29.

39. O'Connell B, Kelly ÁM, Mockler D, Orešič M, Denvir K, Farrell G, et al. Use of Blood Biomarkers in the Assessment of Sports-Related Concussion—A Systematic Review in the Context of Their Biological Significance. Clinical Journal of Sport Medicine. 2018 Nov;28(6):561–71.

40. Iverson GL, Gardner AJ, Terry DP, Ponsford JL, Sills AK, Broshek DK, et al. Predictors of clinical recovery from concussion: a systematic review. Br J Sports Med. 2017 Jun 31;51(12):941–8.

41. van Ierssel J, Pennock KF, Sampson M, Zemek R, Caron JG. Which psychosocial factors are associated with return to sport following concussion? A systematic review. J Sport Health Sci. 2022 Jul;11(4):438–49.

42. Karantali E, Kazis D, McKenna J, Chatzikonstantinou S, Petridis F, Mavroudis I. Neurofilament light chain in patients with a concussion or head impacts: a systematic review and meta-analysis. European Journal of Trauma and Emergency Surgery. 2022 Jun 18;48(3):1555–67.

For peer review only - http://bmjopen.bmj.com/site/about/guidelines.xhtml

43. Silverberg ND, Gardner AJ, Brubacher JR, Panenka WJ, Li JJ, Iverson GL. Systematic Review of Multivariable Prognostic Models for Mild Traumatic Brain Injury. J Neurotrauma. 2015 Apr 15;32(8):517–26.

44. Cancelliere C, Hincapié CA, Keightley M, Godbolt AK, Côté P, Kristman VL, et al. Systematic Review of Prognosis and Return to Play After Sport Concussion: Results of the International Collaboration on Mild Traumatic Brain Injury Prognosis. Arch Phys Med Rehabil. 2014 Mar;95(3):S210–29.

45. Kamins J, Bigler E, Covassin T, Henry L, Kemp S, Leddy JJ, et al. What is the physiological time to recovery after concussion? A systematic review. Br J Sports Med. 2017 Jun;51(12):935–40.

46. Manley G, Gardner AJ, Schneider KJ, Guskiewicz KM, Bailes J, Cantu RC, et al. A systematic review of potential long-term effects of sport-related concussion. Br J Sports Med. 2017 Jun;51(12):969–77.

47. Lindsey HM, Hodges CB, Greer KM, Wilde EA, Merkley TL. Diffusion-Weighted Imaging in Mild Traumatic Brain Injury: A Systematic Review of the Literature. Neuropsychol Rev. 2023 Mar 15;33(1):42–121.

48. Khong E, Odenwald N, Hashim E, Cusimano MD. Diffusion Tensor Imaging Findings in Post-Concussion Syndrome Patients after Mild Traumatic Brain Injury: A Systematic Review. Front Neurol. 2016 Sep 19;7.

49. Lumba-Brown A, Yeates KO, Sarmiento K, Breiding MJ, Haegerich TM, Gioia GA, et al. Centers for Disease Control and Prevention Guideline on the Diagnosis and Management of Mild Traumatic Brain Injury Among Children. JAMA Pediatr. 2018 Nov 5;172(11):e182853.

50. Patricios JS, Schneider KJ, Dvorak J, Ahmed OH, Blauwet C, Cantu RC, et al. Consensus statement on concussion in sport: the 6th International Conference on Concussion in Sport-Amsterdam, October 2022. Br J Sports Med. 2023;57(11).

51. Rademakers J, Delnoij D, De Boer D. Structure, process or outcome: Which contributes most to patients' overall assessment of healthcare quality? BMJ Qual Saf. 2011;20(4).

52. NHS England and NHS Improvement. A model for measuring quality care [online]. [London]: NHS England; 2021. [cited 2023 Oct 5]. Available from: https://www.england.nhs.uk/wp-content/uploads/2022/02/qsir-measuring-quality-care.pdf

For peer review only - http://bmjopen.bmj.com/site/about/guidelines.xhtml

53. Grol R, Grimshaw J. From best evidence to best practice: Effective implementation of change in patients' care. Vol. 362, Lancet. 2003.

54. Bauer MS, Damschroder L, Hagedorn H, Smith J, Kilbourne AM. An introduction to implementation science for the non-specialist. BMC Psychol. 2015;3(1).

55. La Brooy C, Pratt B, Kelaher M. What is the role of consensus statements in a risk society? J Risk Res. 2020;23(5).

56. Foks KA, Cnossen MC, Dippel DWJ, Maas AIR, Menon D, van der Naalt J, et al. Management of Mild Traumatic Brain Injury at the Emergency Department and Hospital Admission in Europe: A Survey of 71 Neurotrauma Centers Participating in the CENTER-TBI Study. J Neurotrauma. 2017 Sep;34(17):2529–35.

57. Jensen KL, Wachner LG, Storm van den Brekel J, Hardis H, Smaakjær P, Hansen M V., et al. Multidisciplinary interventions increase weekly working hours and quality of life in persons with post-concussion syndrome. Neurol Res. 2023 Oct 3;45(10):926–35.

58. Hiploylee C, Dufort PA, Davis HS, Wennberg RA, Tartaglia MC, Mikulis D, et al. Longitudinal study of postconcussion syndrome: Not everyone recovers. J Neurotrauma [Internet]. 2017;34(8):1511–23. Available from: http://www.liebertonline.com/neu

59. Polinder S, Cnossen MC, Real RGL, Covic A, Gorbunova A, Voormolen DC, et al. A Multidimensional Approach to Post-concussion Symptoms in Mild Traumatic Brain Injury. Front Neurol [Internet]. 2018;9. Available from: https://www.scopus.com/inward/record.uri?eid=2-s2.0-85063738495&doi=10.3389%2ffneur.2018.01113&partnerID=40&md5=7da2daff1fa8712a1dffea114975fb89

60. Faulkner JW, Theadom A, Snell DL, Williams MN. Network analysis applied to post-concussion symptoms in two mild traumatic brain injury samples. Front Neurol. 2023 Jul 20;14.

61. Brown L, Camarinos J. The Role of Physical Therapy in Concussion Rehabilitation. Semin Pediatr Neurol [Internet]. 2019;30:68–78. Available from: https://www.scopus.com/inward/record.uri?eid=2-s2.0-85067548442&doi=10.1016%2fj.spen.2019.03.011&partnerID=40&md5=f17daf40e7be68cbd35160ee02771504

For peer review only

For peer review only - http://bmjopen.bmj.com/site/about/guidelines.xhtml

62. Bunt SC, LoBue C, Hynan LS, Didehbani N, Stokes M, Miller SM, et al. Early vs. delayed evaluation and persisting concussion symptoms during recovery in adults. Clin Neuropsychol. 2022 Sep 9;1–18.

63. Maas AIR, Menon DK, Manley GT, Abrams M, Åkerlund C, Andelic N, et al. Traumatic brain injury: progress and challenges in prevention, clinical care, and research. Lancet Neurol. 2022 Nov;21(11):1004–60.

64. Déry J, De Guise É, Bussières ÈL, Lamontagne ME. Prognostic factors for persistent symptoms in adults with mild traumatic brain injury: protocol for an overview of systematic reviews. Syst Rev. 2021;10(1).

65. Voormolen DC, Haagsma JA, Polinder S, Maas AIR, Steyerberg EW, Vuleković P, et al. Post-concussion symptoms in complicated vs. Uncomplicated mild traumatic brain injury patients at three and six months post-injury: Results from the CENTER-TBI study. J Clin Med. 2019;8(11).

66. Yuh EL, Jain S, Sun X, Pisică D, Harris MH, Taylor SR, et al. Pathological Computed Tomography Features Associated with Adverse Outcomes after Mild Traumatic Brain Injury: A TRACK-TBI Study with External Validation in CENTER-TBI. JAMA Neurol. 2021;78(9).

67. Yeates KO, Räisänen AM, Premji Z, Debert CT, Frémont P, Hinds S, et al. What tests and measures accurately diagnose persisting post-concussive symptoms in children, adolescents and adults following sport-related concussion? A systematic review. Vol. 57, British Journal of Sports Medicine. 2023.

68. Longworth T, McDonald A, Cunningham C, Khan H, Fitzpatrick J. Do rugby league players under-report concussion symptoms? A cross-sectional study of elite teams based in Australia. BMJ Open Sport Exerc Med. 2021 Jan 19;7(1):e000860.

69. Tadmor D, Till K, Phillips G, Brown J, Fairbank L, Hendricks S, et al. I won't let you down; why 20% of Men's and Women's Super League players underreported suspected concussions. J Sci Med Sport. 2023 Sep;

70. NIHR Evidence. What is digital health technology and what can it do for me? [online]. [London]. NIHR Evidence; 2022 [cited 2023 Oct 5]. Available from: https://evidence.nihr.ac.uk/collection/what-is-digital-health-technology/

71. WHO Team: Digital Health and Innovation GRCS and RH and RH and IGRCS and RH and R. Recommendations on digital interventions for health system strengthening [Online]. WHO; 2019 [cited 2023 Oct 5]. Available from:

For peer review only - http://bmjopen.bmj.com/site/about/guidelines.xhtml

https://iris.who.int/bitstream/handle/10665/311941/9789241550505-eng.pdf?sequence=31

72. Powell D, Stuart S, Godfrey A. Sports related concussion: an emerging era in digital sports technology. NPJ Digit Med [Internet]. 2021;4(1) (no pagination). Available from: https://www.nature.com/npjdigitalmed/

73. Seabury SA, Gaudette É, Goldman DP, Markowitz AJ, Brooks J, McCrea MA, et al. Assessment of Follow-up Care After Emergency Department Presentation for Mild Traumatic Brain Injury and Concussion. JAMA Netw Open. 2018 May 25;1(1):e180210.

74. Bergman K, Louis S. Discharge Instructions for Concussion: Are We Meeting the Patient Needs? Journal of Trauma Nursing. 2016 Nov;23(6):327–33.

75. Tan CL, Alavi SA, Baldeweg SE, Belli A, Carson A, Feeney C, et al. The screening and management of pituitary dysfunction following traumatic brain injury in adults: British Neurotrauma Group guidance. J Neurol Neurosurg Psychiatry. 2017 Nov;88(11):971–81.

76. Birko S, Dove ES, Özdemir V, Dalal K. Evaluation of nine consensus indices in delphi foresight research and their dependency on delphi survey characteristics: A simulation study and debate on delphi design and interpretation. PLoS One. 2015;10(8).

77. de Villiers MR, de Villiers PJT, Kent AP. The Delphi technique in health sciences education research. Med Teach. 2005;27(7).

For peer review only

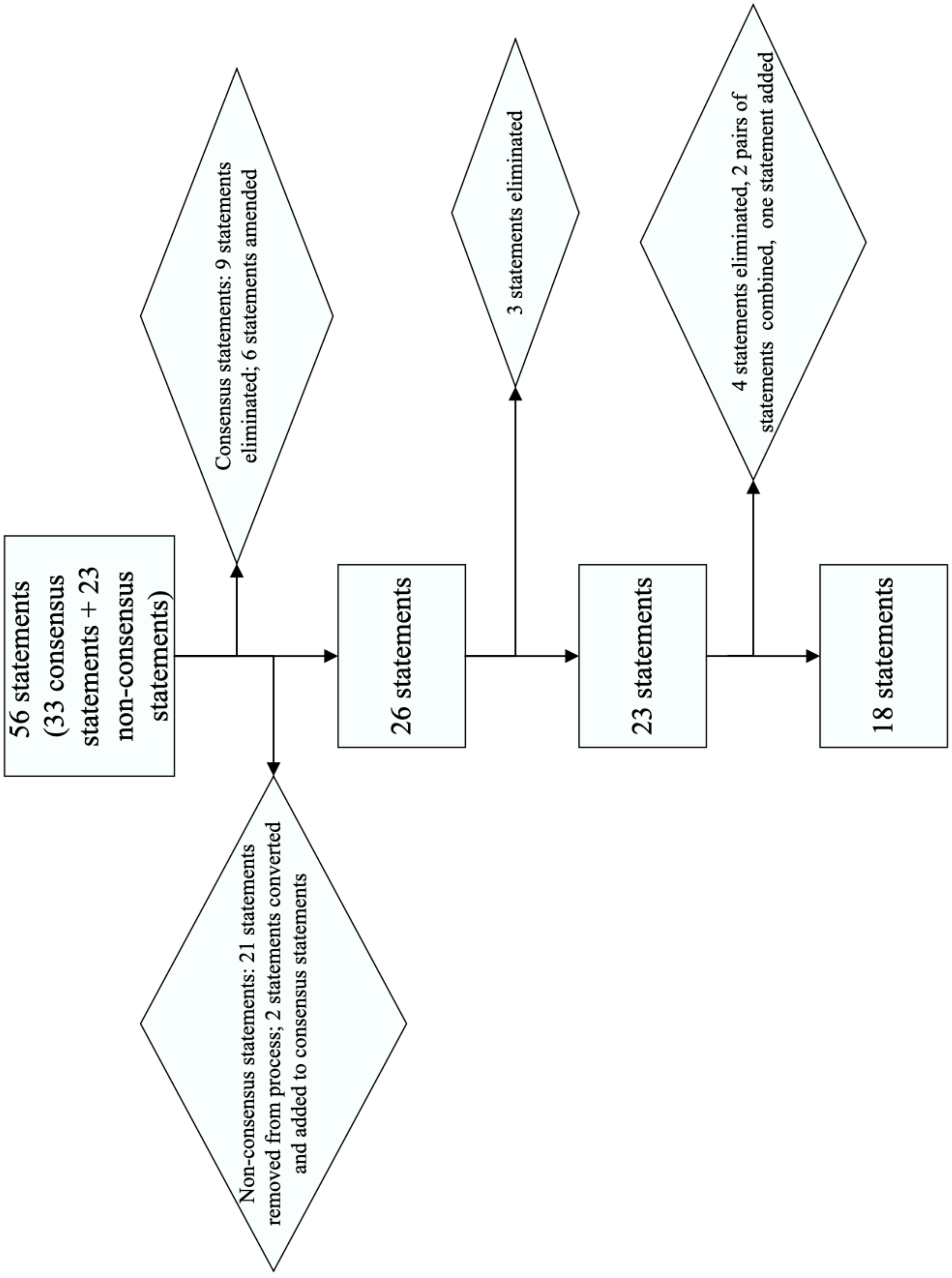
56 statements (33 consensus statements + 23 non-consensus statements)
Consensus statements: 9 statements eliminated; 6 statements amended
Non-consensus statements: 21 statements removed from process; 2 statements converted and added to consensus statements
26 statements
3 statements eliminated
23 statements
4 statements eliminated, 2 pairs of statements combined, one statement added
18 statements

Supplementary material 1: Literature search strategy

The search was conducted in MEDLINE and Embase. Below is the search used for Embase and the equivalent search was done for MEDLINE.

Embase search strategy

1 brain concussion/

2 postconcussion syndrome/

3 "mild traumatic brain injur*".mp. [mp=title, abstract, heading word, drug trade name, original title, device manufacturer, drug manufacturer, device trade name, keyword heading word, floating subheading word, candidate term word]

4 "concussion*".mp. [mp=title, abstract, heading word, drug trade name, original title, device manufacturer, drug manufacturer, device trade name, keyword heading word, floating subheading word, candidate term word]

5 "concussive*".mp. [mp=title, abstract, heading word, drug trade name, original title, device manufacturer, drug manufacturer, device trade name, keyword heading word, floating subheading word, candidate term word]

6 "mild head injur*".mp. [mp=title, abstract, heading word, drug trade name, original title, device manufacturer, drug manufacturer, device trade name, keyword heading word, floating subheading word, candidate term word]

7 "mild brain injur*".mp. [mp=title, abstract, heading word, drug trade name, original title, device manufacturer, drug manufacturer, device trade name, keyword heading word, floating subheading word, candidate term word]

8 "mtbi*".mp. [mp=title, abstract, heading word, drug trade name, original title, device manufacturer, drug manufacturer, device trade name, keyword heading word, floating subheading word, candidate term word]

9 "mild TBI*".mp. [mp=title, abstract, heading word, drug trade name, original title, device manufacturer, drug

For peer review only - http://bmjopen.bmj.com/site/about/guidelines.xhtml

manufacturer, device trade name, keyword heading word, floating subheading word, candidate term word]

10 "commotio cerebri".mp. [mp=title, abstract, heading word, drug trade name, original title, device manufacturer,
drug manufacturer, device trade name, keyword heading word, floating subheading word, candidate term word]

11 "post-concussion symptom*".mp. [mp=title, abstract, heading word, drug trade name, original title, device
manufacturer, drug manufacturer, device trade name, keyword heading word, floating subheading word, candidate term word]

12 "post-concussion syndrome*".mp. [mp=title, abstract, heading word, drug trade name, original title, device
manufacturer, drug manufacturer, device trade name, keyword heading word, floating subheading word, candidate term word]

13 1 or 2 or 3 or 4 or 5 or 6 or 7 or 8 or 9 or 10 or 11 or 12

14 practice guideline/

15 clinical practice/

16 "guideline*".mp. [mp=title, abstract, heading word, drug trade name, original title, device manufacturer, drug
manufacturer, device trade name, keyword heading word, floating subheading word, candidate term word]

17 "clinical practic*".mp. [mp=title, abstract, heading word, drug trade name, original title, device manufacturer,
drug manufacturer, device trade name, keyword heading word, floating subheading word, candidate term word]

18 "recommend*".mp. [mp=title, abstract, heading word, drug trade name, original title, device manufacturer, drug
manufacturer, device trade name, keyword heading word, floating subheading word, candidate term word]

19 "best practic*".mp. [mp=title, abstract, heading word, drug trade name, original title, device manufacturer, drug
manufacturer, device trade name, keyword heading word, floating subheading word, candidate term word]

For peer review only - http://bmjopen.bmj.com/site/about/guidelines.xhtml

20 "clinical decision*".mp. [mp=title, abstract, heading word, drug trade name, original title, device manufacturer,
drug manufacturer, device trade name, keyword heading word, floating subheading word, candidate term word]
21 "clinical support*".mp. [mp=title, abstract, heading word, drug trade name, original title, device manufacturer,
drug manufacturer, device trade name, keyword heading word, floating subheading word, candidate term word]
22 "consensus".mp. [mp=title, abstract, heading word, drug trade name, original title, device manufacturer, drug
manufacturer, device trade name, keyword heading word, floating subheading word, candidate term word]
23 "position statement*".mp. [mp=title, abstract, heading word, drug trade name, original title, device
manufacturer, drug manufacturer, device trade name, keyword heading word, floating subheading word, candidate term word]
24 healthcare service.mp. or health service/
25 "*care system*".mp. [mp=title, abstract, heading word, drug trade name, original title, device manufacturer, drug
manufacturer, device trade name, keyword heading word, floating subheading word, candidate term word]
26 "service*".mp. [mp=title, abstract, heading word, drug trade name, original title, device manufacturer, drug
manufacturer, device trade name, keyword heading word, floating subheading word, candidate term word]
27 14 or 15 or 16 or 17 or 18 or 19 or 20 or 21 or 22 or 23 or 24 or 25 or 26 (3935187)
28 13 and 27

Supplementary material 2: Statements voting round 1

Supplementary material 2.1 Consensus Statement Voting Round 1

Statement	Agreement	Outcome
There should be a direct patient pathway from ED to specialist outpatient services available to	71%	None

all patients to ensure patients with persistent symptoms will be identified early.		
There is not sufficient evidence for injury-related events (i.e. presence of loss of consciousness, post-traumatic amnesia, vomiting) to predict prolonged symptoms.	71%	None
There is not sufficient evidence to support the clinical application of objective biomarkers (i.e. fluid, imaging) for identifying and screening for persistent symptoms after a mild TBI.	81%	None
There is not enough evidence to use prognostic tools to predict the risk of developing persistent symptoms.	81%	None
All mild TBI patients should be screened for persistent symptoms	67%	Eliminated
All mild TBI patients should receive follow-up in specialist clinics	19%	Eliminated
There should be a system for patients to be triaged to a specialist in (mild) TBI clinics based on persistent symptoms.	95%	Amended
All patients with history of 2 or more head injuries should be seen in specialist clinics regardless of whether they experience persistent symptoms.	29%	Eliminated
All patients with history of neurological conditions (i.e. migraines, epilepsy) should be seen in specialist clinics regardless of whether they experience persistent symptoms.	14%	Eliminated
All patients with history of psychological problems (i.e. depression, anxiety, PTSD) should be seen in specialist clinics regardless of whether they experience persistent symptoms.	10%	Eliminated

For peer review only - http://bmjopen.bmj.com/site/about/guidelines.xhtml

Only symptomatic patients should be seen in specialist clinics.	81%	Amended
Digital tools should be utilised to screen for persistent symptoms.	71%	Amended
Symptom scales are currently the most appropriate method available for measuring symptom burdens in patients.	71%	Amended
Symptom scales are currently the most appropriate method available to screen for persistent symptoms in patients.	71%	None
A bespoke symptom scale, such as the Rivermead or Post-Concussion Symptom Scale, should be used for screening symptoms.	80%	Amended
All patients should complete a symptom scale at the first point of contact with a clinician in the acute phase.	71%	None
Patients screening negative for PPCS on the survey at any point should be discharged from specialist service.	67%	Eliminated
Patients should be under the care of their regional mild TBI clinic until symptom resolution, and only discharged once fully recovered and returned to work, where applicable.	52%	Eliminated
The extended Glasgow Outcome Scale (eGOS) should be used to measure functional outcomes after mild TBI in routine clinical practice.	50%	Amended
The SF-36 or EQ-5D should be used to measure quality of life after mild TBI in routine clinical practice.	35%	Eliminated
A bespoke patient-reported outcome measure (PROM) should be developed for mild TBI and used in routine clinical practice	70%	None

For peer review only - http://bmjopen.bmj.com/site/about/guidelines.xhtml

There should be dedicated mild TBI clinics, or where not possible, TBI clinics with dedicated time to mild TBI patients.	100%	None
Specialist (mild) TBI clinics should comprise of a multidisciplinary team.	90%	None
All patients should be directed to a single bespoke online information portal on mild TBI to help with expectations and recommendations.	90%	None
Patients should be advised to rest (cognitive and physical rest) for 24-48 hours, after which they should gradually increase activity.	75%	None
Patients should be advised to rest (cognitive and physical rest) for as long as they are experiencing symptoms	25%	Eliminated
There is not sufficient evidence to support the use of advanced imaging techniques in clinical practice.	80%	None
There is not sufficient evidence to support screening for symptom subtypes. 10. Investigations and treatments should be symptom-based.	80%	Amended
All patients with persistent symptoms should complete a cognitive assessment reviewed by a neuropsychologist	-*	Amended
All clinics should have access to standardised protocols for assessing pituitary dysfunction.	90%	None
Adjusting expectation and education should be the primary initial approach when managing mild TBI, regardless of symptoms.	85%	None
Co-morbidities should be factored into clinical decision-making on management.	100%	None

For peer review only - http://bmjopen.bmj.com/site/about/guidelines.xhtml

Socioeconomic status and social support should be considered during clinical decision-making on management.	90%	None
Where patients are involved in sports, they should be advised to return to work or studies without symptom exacerbation before returning to unsupervised physical exercise.	70%	None
Low-intensity physical activity should be recommended as long as it does not exacerbate symptoms.	100%	None
Treatments should be assessed on a case-by-case basis.	-**	Amended

*The format of the response options for the statement was amended following voting and degree of agreement is therefore unavailable. **9 statements about symptom specific treatments were combined. The format of the response options for the statement was amended following voting and degree of agreement is therefore unavailable.

ED, Emergency Department; EQ-5D, EurQol-5 Dimensions; PPCS, Persistent Post-Concussion Symptoms; PTSD, Post Traumatic Stress Disorder; SF-36, Short-Form 36; TBI, Traumatic Brain Injury

Supplementary material 2.2 Additional statements

**Statements**	**Response options**
All patients should be screened for symptoms	2 weeks after injury<br>4 weeks after injury<br>6 weeks after injury<br>8 weeks after injury<br>10 weeks after injury<br>12 weeks after injury<br>All the above<br>Never<br>Other (open text)

For peer review only - http://bmjopen.bmj.com/site/about/guidelines.xhtml

Patients should be treated in primary care settings and only referred to specialist services if they do not improve within	2 weeks of injury<br>4 weeks of injury<br>6 weeks of injury<br>8 weeks of injury<br>10 weeks of injury<br>12 weeks of injury<br>Never<br>Other (open text)
Patients with persistent symptoms should first be reviewed in specialist clinic within	2 weeks of injury<br>4 weeks of injury<br>6 weeks of injury<br>8 weeks of injury<br>10 weeks of injury<br>12 weeks of injury<br>Never<br>Other (open text)
Patients should be followed-up in specialist clinic (in-person or telephone follow-up, depending on need)	Every 2 weeks until full recovery<br>Every 4 weeks until full recovery<br>Every 6 weeks until full recovery<br>Every 8 weeks until full recovery<br>Every 10 weeks until full recovery<br>Every 12 weeks until full recovery<br>None of the above, interval between reviews should increase with time as patients improve<br>None of the above, patients should only be reviewed once in clinic<br>Case-by-case assessment<br>Other (open text)
Criteria for clinic follow-up should be if they have	1 or more mild-severe symptoms<br>2 or more mild-severe symptoms<br>3 or more mild-severe symptoms

For peer review only - http://bmjopen.bmj.com/site/about/guidelines.xhtml

	1 or more moderate-severe symptoms<br>2 or more moderate-severe symptoms<br>3 or more moderate-severe symptoms.<br>None of the above (no set criteria) – symptom scales should always be interpreted by an experienced physician on a case-by-case basis<br>Other (open text)
The initial screening of patients should be done by	Automated system<br>Specialist nurse<br>Occupational therapist<br>Physical therapist<br>Neuropsychologist<br>Neurologist<br>Neurosurgeon<br>Neurorehabilitation specialist<br>Emergency medicine physician<br>Sport and exercise medicine physician<br>Other (open text)
The first review in specialist clinic should be done by	Automated system<br>Specialist nurse<br>Occupational therapist<br>Physical therapist<br>Neuropsychologist<br>Neurologist<br>Neurosurgeon<br>Neurorehabilitation specialist<br>Emergency medicine physician

For peer review only - http://bmjopen.bmj.com/site/about/guidelines.xhtml

	Sport and exercise medicine physician<br>Other (open text)
The first review in specialist clinic should be	Face-to-face<br>Telephone follow-up<br>Always depend on need based on the triage<br>None of the above<br>Other (open text)
New or worsening symptoms developed more than 1 month after injury should be investigated for non-head injury related causes under	The mild TBI specialist outpatient service.<br>Primary care services.<br>Other (open text)
A bespoke patient-reported outcome measure (PROM) should be developed for mild TBI and used in routine clinical practice	To measure functional outcomes<br>To measure quality of life<br>To measure symptom burden<br>All the above<br>None of the above<br>Other (open text)
At a minimum, the multidisciplinary team should consist of	Neurosurgeons<br>Neurologists<br>Neuropsychologist<br>Vestibular specialist<br>Physiotherapists<br>Specialist nurse<br>Occupational therapist<br>Neurorehabilitation specialist<br>Sport and exercise medicine physician<br>All the above<br>None of the above<br>Other (open text)

For peer review only - http://bmjopen.bmj.com/site/about/guidelines.xhtml

In addition to the core MDT, there should be a direct access to	Ophthalmologists<br>Sleep clinics<br>Headache/migraine specialist clinic<br>Internet-based Cognitive Behavioural Therapy<br>Community-level services<br>All the above<br>None of the above<br>Other (open text)
Patients with the following symptom(s) should have an MRI to exclude gross structural abnormalities.	Low mood<br>Anxiety<br>Memory problems<br>Concentration problems<br>Taking longer to think<br>Restlessness<br>Irritable<br>Feeling frustrated<br>Fatigue<br>Sleep disturbance<br>Nausea<br>Dizziness (without an obvious underlying vestibular pathology)<br>Headache<br>Sensitivity to noise<br>Sensitivity to light<br>Visual symptoms (without an obvious underlying visual pathology)<br>None of the above<br>All the above<br>Case-by-case assessment

For peer review only - http://bmjopen.bmj.com/site/about/guidelines.xhtml
For peer review only

All patients with persistent symptoms should complete a cognitive assessment reviewed by a neuropsychologist*	In-person in clinic<br>Online in clinic<br>Online at home<br>Any of the above<br>None of the above<br>Other (open text)
Patients with the following symptom(s) should be referred to neuropsychology or psychiatry for support or cognitive rehabilitation**	Low mood<br>Anxiety<br>Memory problems<br>Concentration problems<br>Taking longer to think<br>Restlessness<br>Irritable<br>Feeling frustrated<br>Fatigue<br>Sleep disturbance<br>Nausea<br>Dizziness (without an obvious underlying vestibular pathology)<br>Headache<br>Sensitivity to noise<br>Sensitivity to light<br>Visual symptoms (without an obvious underlying visual pathology)<br>None of the above<br>All the above<br>Case-by-case assessment
Patients with the following symptom(s) should be referred for physical therapy for physical rehabilitation**	Low mood<br>Anxiety<br>Memory problems<br>Concentration problems<br>Taking longer to think

For peer review only - http://bmjopen.bmj.com/site/about/guidelines.xhtml

	Restlessness<br>Irritable<br>Feeling frustrated<br>Fatigue<br>Sleep disturbance<br>Nausea<br>Dizziness (without an obvious underlying vestibular pathology)<br>Headache<br>Sensitivity to noise<br>Sensitivity to light<br>Visual symptoms (without an obvious underlying visual pathology)<br>None of the above<br>All the above<br>Case-by-case assessment
Patients with the following symptom(s) should be referred for cervical rehabilitation**	Low mood<br>Anxiety<br>Memory problems<br>Concentration problems<br>Taking longer to think<br>Restlessness<br>Irritable<br>Feeling frustrated<br>Fatigue<br>Sleep disturbance<br>Nausea<br>Dizziness (without an obvious underlying vestibular pathology)<br>Headache<br>Sensitivity to noise<br>Sensitivity to light

For peer review only - http://bmjopen.bmj.com/site/about/guidelines.xhtml

	Visual symptoms (without an obvious underlying visual pathology)<br>None of the above<br>All the above<br>Case-by-case assessment
Patients with the following symptom(s) should be referred for subsymptom threshold aerobic exercise**	Low mood<br>Anxiety<br>Memory problems<br>Concentration problems<br>Taking longer to think<br>Restlessness<br>Irritable<br>Feeling frustrated<br>Fatigue<br>Sleep disturbance<br>Nausea<br>Dizziness (without an obvious underlying vestibular pathology)<br>Headache<br>Sensitivity to noise<br>Sensitivity to light<br>Visual symptoms (without an obvious underlying visual pathology)<br>None of the above<br>All the above<br>Case-by-case assessment
Patients with the following symptom(s) should be referred for vestibular rehabilitation**	Low mood<br>Anxiety<br>Memory problems<br>Concentration problems<br>Taking longer to think

For peer review only - http://bmjopen.bmj.com/site/about/guidelines.xhtml
For peer review only

	Restlessness<br>Irritable<br>Feeling frustrated<br>Fatigue<br>Sleep disturbance<br>Nausea<br>Dizziness (without an obvious underlying vestibular pathology)<br>Headache<br>Sensitivity to noise<br>Sensitivity to light<br>Visual symptoms (without an obvious underlying visual pathology)<br>None of the above<br>All the above<br>Case-by-case assessment
Patients with the following symptom(s) should be referred for cervical rehabilitation**	Low mood<br>Anxiety<br>Memory problems<br>Concentration problems<br>Taking longer to think<br>Restlessness<br>Irritable<br>Feeling frustrated<br>Fatigue<br>Sleep disturbance<br>Nausea<br>Dizziness (without an obvious underlying vestibular pathology)<br>Headache<br>Sensitivity to noise<br>Sensitivity to light

For peer review only

	Visual symptoms (without an obvious underlying visual pathology)<br>None of the above<br>All the above<br>Case-by-case assessment
Patients with the following symptom(s) should be referred for hyperbaric oxygen therapy**	Low mood<br>Anxiety<br>Memory problems<br>Concentration problems<br>Taking longer to think<br>Restlessness<br>Irritable<br>Feeling frustrated<br>Fatigue<br>Sleep disturbance<br>Nausea<br>Dizziness (without an obvious underlying vestibular pathology)<br>Headache<br>Sensitivity to noise<br>Sensitivity to light<br>Visual symptoms (without an obvious underlying visual pathology)<br>None of the above<br>All the above<br>Case-by-case assessment
Patients with the following symptom(s) should be referred for transcranial magnetic stimulation.**	Low mood<br>Anxiety<br>Memory problems<br>Concentration problems<br>Taking longer to think

For peer review only

	Restlessness<br>Irritable<br>Feeling frustrated<br>Fatigue<br>Sleep disturbance<br>Nausea<br>Dizziness (without an obvious underlying vestibular pathology)<br>Headache<br>Sensitivity to noise<br>Sensitivity to light<br>Visual symptoms (without an obvious underlying visual pathology)<br>None of the above<br>All the above<br>Case-by-case assessment
There is not sufficient evidence for any pharmacological interventions to be routinely prescribed for persistent symptoms after mild TBI for the following symptom(s).**	Low mood<br>Anxiety<br>Memory problems<br>Concentration problems<br>Taking longer to think<br>Restlessness<br>Irritable<br>Feeling frustrated<br>Fatigue<br>Sleep disturbance<br>Nausea<br>Dizziness (without an obvious underlying vestibular pathology)<br>Headache<br>Sensitivity to noise<br>Sensitivity to light

For peer review only - http://bmjopen.bmj.com/site/about/guidelines.xhtml

	Visual symptoms (without an obvious underlying visual pathology)<br>None of the above<br>All the above<br>Case-by-case assessment

*Statement was amended and added as consensus statements in the second round of voting.

**Statements were added to get an overview of the panel's opinion regarding specific therapies for symptom profiles. The results resulted in the final statement denoted in supplementary material 2.1.

MDT, multidisciplinary team; MRI, Magnetic Resonance Imaging; TBI, Traumatic Brain Injury

Supplementary material 3: Consensus Statement Voting Round 2

Statement included in voting round 2	Agreement	Outcome
There should be a direct patient pathway from ED to specialist outpatient services available to all patients to ensure patients with persistent symptoms will be identified early.	81%	None
There is not sufficient evidence for injury-related events (i.e. presence of loss of consciousness, post-traumatic amnesia, vomiting) to predict prolonged symptoms.	80%	None
There is not sufficient evidence to support the clinical application of objective biomarkers (i.e. fluid, imaging) for identifying and screening for persistent symptoms after a mild TBI.	90%	None
There is not enough evidence to use prognostic tools to predict the risk of developing persistent symptoms.	75%	None
There should be a system for patients to be triaged to a specialist in (mild) TBI clinics based on persistent symptoms.	100%	None

For peer review only - http://bmjopen.bmj.com/site/about/guidelines.xhtml

Mild TBI patients with persistent symptoms should be seen in specialist clinics.	90%	None
Digital tools should be utilised to screen for persistent symptoms.	89%	None
Symptom scales are currently the most appropriate method available for measuring symptom burdens in patients.	74%	None
Symptom scales are currently the most appropriate method available to screen for persistent symptoms in patients.	74%	None
A bespoke symptom scale should be used for screening symptoms.	86%	None
All patients should complete a symptom scale at the first point of contact with a clinician in the acute phase.	70%	None
A bespoke patient-reported outcome measure (PROM) should be developed for mild TBI and used in routine clinical practice.	95%	None
There should be dedicated mild TBI clinics, or where not possible, TBI clinics with dedicated time to mild TBI patients.	95%	None
Specialist (mild) TBI clinics should comprise of a multidisciplinary team.	95%	None
All patients should be directed to a single bespoke online information portal on mild TBI to help with expectations and recommendations.	81%	None
Patients should be advised to rest (cognitive and physical rest) for 24-48 hours, after which they should gradually increase activity.	75%	None
There is not sufficient evidence to support the use of advanced imaging techniques in clinical practice.	81%	None

For peer review only - http://bmjopen.bmj.com/site/about/guidelines.xhtml

There is not sufficient evidence to support screening for clinical subtypes based on symptom clusters.	67%	Eliminated
All patients with persistent symptoms should complete a cognitive assessment reviewed by a neuropsychologist.	42%	Eliminated
All clinics should have access to standardised protocols for assessing pituitary dysfunction.	76%	None
Adjusting expectation and education should be the primary initial approach when managing mild TBI, regardless of symptoms.	90%	None
Co-morbidities should be factored into clinical decision-making on management.	100%	None
Socioeconomic status and social support should be considered during clinical decision-making on management.	86%	None
Where patients are involved in sports, they should be advised to return to work or studies without symptom exacerbation before returning to unsupervised physical exercise.	62%	Eliminated
Low-intensity physical activity should be recommended as long as it does not exacerbate symptoms.	95%	None
Treatment should be symptom-based and assessed on a case-by-case basis as there is currently not sufficient evidence to support a specific treatment approach for all mild TBI patients.	95%	None

ED, Emergency Department; TBI, Traumatic Brain Injury

For peer review only - http://bmjopen.bmj.com/site/about/guidelines.xhtml

Rationale for the choice of the Delphi technique	Report on page(s) in main document
1. *Justification.* The choice of the Delphi technique as a method of systematically collating expert consultation and building consensus needs to be well justified. When selecting the method to answer a particular research question, it is important to keep in mind its constructivist nature	1-2
Planning and design	
2. *Planning and process.* The Delphi technique is a flexible method and can be adjusted to the respective research aims and purposes. Any modifications should be justified by a rationale and be applied systematically and rigorously	2-4
3. *Definition of consensus.* Unless not reasonable due to the explorative nature of the study, an a priori criterion for consensus should be defined. This includes a clear and transparent guide for action on (a) how to proceed with certain items or topics in the next survey round, (b) the required threshold to terminate the Delphi process and (c) procedures to be followed when consensus is (not) reached after one or more iterations	3-4
Study conduct	
4. *Informational input.* All material provided to the expert panel at the outset of the project and throughout the Delphi process should be carefully reviewed and piloted in advance in order to examine the effect on experts' judgements and to prevent bias	3
5. *Prevention of bias.* Researchers need to take measures to avoid directly or indirectly influencing the experts' judgements. If one or more members of the research team have a conflict of interest, entrusting an independent researcher with the main coordination of the Delphi study is advisable	2
6. *Interpretation and processing of results.* Consensus does not necessarily imply the 'correct' answer or judgement; (non)consensus and stable disagreement provide informative insights and highlight differences in perspectives concerning the topic in question	3-4
Reporting	
7. *Purpose and rationale.* The purpose of the study should be clearly defined and demonstrate the appropriateness of the use of the Delphi technique as a method to achieve the research aim. A rationale for the choice of the Delphi technique as the most suitable method needs to be provided	1-2
8. *Expert panel.* Criteria for the selection of experts and transparent information on recruitment of the expert panel, socio-demographic details including information on expertise regarding the topic in question, (non)response and response rates over the ongoing iterations should be reported	2
9. *Description of the methods.* The methods employed need to be comprehensible; this includes information on preparatory steps (How was available evidence on the topic in question synthesised?), piloting of material and survey instruments, design of the survey instrument(s), the number and design of survey rounds, methods of data analysis,	2-4

processing and synthesis of experts' responses to inform the subsequent survey round and methodological decisions taken by the research team throughout the process	
10. *Procedure.* Flow chart to illustrate the stages of the Delphi process, including a preparatory phase, the actual 'Delphi rounds', interim steps of data processing and analysis, and concluding steps	2
11. *Definition and attainment of consensus.* It needs to be comprehensible to the reader how consensus was achieved throughout the process, including strategies to deal with non-consensus	3-4
12. *Results.* Reporting of results for each round separately is highly advisable in order to make the evolving of consensus over the rounds transparent. This includes figures showing the average group response, changes between rounds, as well as any modifications of the survey instrument such as deletion, addition or modification of survey items based on previous rounds	4-8
13. *Discussion of limitations.* Reporting should include a critical reflection of potential limitations and their impact of the resulting guidance	12-13
14. *Adequacy of conclusions.* The conclusions should adequately reflect the outcomes of the Delphi study with a view to the scope and applicability of the resulting practice guidance	13
15. *Publication and dissemination.* The resulting guidance should be clearly identifiable from the publication, including recommendations for transfer into practice and implementation. If the publication does not allow for a detailed presentation of either the resulting practice guidance or the methodological features of the applied Delphi technique, or both, reference to a more detailed presentation elsewhere should be made (e.g. availability of the full guideline from the authors or online; publication of a separate paper reporting on methodological details and particularities of the process (e.g. persistent disagreement and controversy on certain issues)). A dissemination plan should include endorsement of the guidance by professional associations and health care authorities to facilitate implementation	8-12

only

BMJ Open

Specialist Healthcare Services for Concussion/Mild Traumatic Brain Injury in England: A Consensus Statement using Modified Delphi Methodology

Journal:	*BMJ Open*
Manuscript ID	bmjopen-2023-077022.R2
Article Type:	Original research
Date Submitted by the Author:	10-Nov-2023

Complete List of Authors:	Karvandi, Elika; University of Cambridge, Neurosurgery Department of Neurosurgery<br>Helmy, Adel; University of Cambridge, Department of Neurosurgery<br>Kolias, Angelos; University of Cambridge, Department of Neurosurgery<br>belli, antonio; University of Birmingham, Inflammation and Ageing<br>Ganau, Mario; University of Oxford Nuffield Department of Clinical Neurosciences<br>Gomes, Clint; Royal Liverpool University Hospital; UK Sports Institute<br>Grey, Michael; Loughborough University, School of Sport, Exercise and Health Sciences<br>Griffiths, Michael; University of Liverpool, Department of Clinical Infection, Microbiology and Immunology, Institute of Infection, Veterinary and Ecological Sciences; Alder-Hey Children's NHS Trust, Department of Paediatric Neurology<br>Griffiths, Timothy; Newcastle University, Department of Cognitive Neurology; University College London, Institute of Neurology<br>Griffiths, Philippa; Northumberland Tyne and Wear NHS Foundation Trust, Sunderland & South Tyneside Community Acquired Brain Injury Service<br>Holliman, Damian; Royal Victoria Infirmary, Department of Neurosurgery<br>Jenkins, Peter; Southampton General Hospital, Wessex Neuroscience Centre; Imperial College London<br>Jones, Ben; Leeds Beckett University - Headingley Campus, Carnegie Applied Rugby Research (CARR) centre; Rugby Football League Ltd, England Performance Unit<br>Lawrence, Tim; University of Oxford, Nuffield Department of Clinical Neurosciences<br>McLoughlin, Terence; Royal Liverpool University Hospital<br>McMahon, Catherine; The Walton Centre NHS Foundation Trust<br>Messahel, Shrouk; Alder Hey Children's Hospital NHS Foundation Trust<br>Newton, Joanne; Newcastle Upon Tyne Hospitals NHS Foundation Trust<br>Noad, Rupert; University Hospitals Plymouth NHS Trust<br>Raymont, Vanessa; University of Oxford, Department of Psychiatry<br>Sharma, Kanchan; North Bristol NHS Trust, Department of Neurology<br>Sylvester, Richard; National Hospital for Neurology and Neurosurgery; University College London, Institute of Exercise and Health<br>Tadmor, Daniel; Leeds Beckett University, Carnegie School of Sport; Leeds Rhinos Rugby League Club, Medical<br>Whitfield, Peter; University Hospitals Plymouth NHS Trust

	Wilson, Mark; Imperial College Healthcare NHS Trust, Department of Neurosurgery; Imperial College London<br>Woodberry, Emma; University of Cambridge, Department of Neuropsychology<br>Parker, Michael; Love of the Game<br>Hutchinson, Peter; University of Cambridge, Neurosurgery
**Primary Subject Heading**:	Health services research
Secondary Subject Heading:	Public health, Neurology
Keywords:	Neurological injury < NEUROLOGY, Patient-Centered Care, PUBLIC HEALTH

SCHOLARONE™ Manuscripts

I, the Submitting Author has the right to grant and does grant on behalf of all authors of the Work (as defined in the below author licence), an exclusive licence and/or a non-exclusive licence for contributions from authors who are: i) UK Crown employees; ii) where BMJ has agreed a CC-BY licence shall apply, and/or iii) in accordance with the terms applicable for US Federal Government officers or employees acting as part of their official duties; on a worldwide, perpetual, irrevocable, royalty-free basis to BMJ Publishing Group Ltd ("BMJ") its licensees and where the relevant Journal is co-owned by BMJ to the co-owners of the Journal, to publish the Work in this journal and any other BMJ products and to exploit all rights, as set out in our licence.

The Submitting Author accepts and understands that any supply made under these terms is made by BMJ to the Submitting Author unless you are acting as an employee on behalf of your employer or a postgraduate student of an affiliated institution which is paying any applicable article publishing charge ("APC") for Open Access articles. Where the Submitting Author wishes to make the Work available on an Open Access basis (and intends to pay the relevant APC), the terms of reuse of such Open Access shall be governed by a Creative Commons licence – details of these licences and which Creative Commons licence will apply to this Work are set out in our licence referred to above.

Other than as permitted in any relevant BMJ Author's Self Archiving Policies, I confirm this Work has not been accepted for publication elsewhere, is not being considered for publication elsewhere and does not duplicate material already published. I confirm all authors consent to publication of this Work and authorise the granting of this licence.

For peer review only

Specialist Healthcare Services for Concussion/Mild Traumatic Brain Injury in England: A Consensus Statement using Modified Delphi Methodology

ABSTRACT

Objective: To establish a consensus on the structure and process of healthcare services for concussion patients in England to facilitate better healthcare quality and patient outcome.

Design: This consensus study followed the modified Delphi methodology with five phases; participant identification, item development, two rounds of voting, and a meeting to finalise the consensus statements. The pre-defined threshold for agreement was set at ≥70%.

Setting: Specialist outpatient services.

Participants: Members of the UK Head Injury Network were invited to participate. The network consists of clinical specialists in head injury practising in emergency medicine, neurology, neuropsychology, neurosurgery, paediatric medicine, rehabilitation medicine and sports and exercise medicine in England.

Primary outcome measure: A consensus statement on the structure and process of specialist outpatient care for concussion patients in England.

Results: 56 items were voted on in the first round. 30 items were removed following the first voting round and 3 items were removed following the second voting round. Items were modified where appropriate. A final 18 statements reached consensus covering three main topics in specialist healthcare services for concussion; care pathway to structured follow-up, prognosis and measures of recovery, and provision of outpatient clinics.

Conclusions: This work presents statements on how the healthcare services for concussion patients in England could be redesigned to meet their health needs. Future work will seek to implement these into the clinical pathway.

For peer review only

For peer review only - http://bmjopen.bmj.com/site/about/guidelines.xhtml

Strengths and limitations of this study

- This consensus statement outlines structural and process-based factors needed to move towards standardised best-practice care for concussions in England to improve healthcare quality and patient outcomes
- To our knowledge, no studies have reported on the healthcare system for concussion in England
- We engaged with various stakeholders involved in concussion care to ensure different perspectives are represented
- It cannot be excluded that some disagreement on statements was in part due to differences in definitions and terminology use despite probable broad agreement on the statement in principle.
- The statements reached agreement in this panel, but we cannot exclude the possibility or to what degree the results are influenced by the experience and expertise of these individuals both for included and excluded statements.

For peer review only

INTRODUCTION

In England and Wales alone, more than a million people suffer a head injury every year (1). These injuries occur as a result of direct or indirect impacts to the head and are commonly encountered in sports, both at professional and grassroots levels. Injuries to the head, particularly in the context of sport, are often labelled "concussions". While there is a recently published definition available (2), there is no widespread consensus for what constitutes a concussion, and it is often used to describe both the symptoms suffered following a head injury as well as the pathophysiological mechanisms causing these symptoms (3,4). Despite some controversies in the field, concussion is widely recognised by the public and those playing sport, and for this reason, we have used the term for the purpose of this paper. A concussion is diagnosed on clinical grounds with evidence of immediate and transient neurological dysfunction that may or may not present with a wide range of signs and symptoms, such as loss of consciousness, post-traumatic amnesia, headache and vomiting (5). However, most concussions do not present with obvious neurological signs, such as loss of consciousness, making recognition and diagnosis challenging (6–9).

The post-concussion sequelae are assumed to recover spontaneously within a few days to a couple of weeks. Yet a proportion of individuals experience persistent symptoms for months to years after injury that requires timely and appropriate medical assessment and treatment (10). Despite this known fact, guidance on clinical practice in the outpatient setting after the initial medial review is limited and ambiguous leaving the concussion population vulnerable to variations and sub-optimal care (11). This results in delayed intervention, resource waste, care inequalities and poorer quality of care (12–14). There is no standardised, structured follow-up for those who experience persistent symptoms and current clinical services largely fail to meet the healthcare needs of these patients (15).

Nonetheless, the past two decades have seen an exponential growth in concussion research which has improved our understanding of the injury and strategies to enhance recovery and quality of care (16). Additionally, there is increasingly more awareness around the potential lasting effects of concussions, especially in sports, where there are growing concerns and calls for action (17). The government and sports' national governing bodies are attempting to improve concussion management in all levels of sports by standardising protocols, allocating more funds for concussion research and incorporating new playing rules to reduce concussion risk and improve management (18). Consequently, the demand for care is evolving and to

For peer review only - http://bmjopen.bmj.com/site/about/guidelines.xhtml

meet patient needs changes to clinical services are required to provide evidence-based best-practice care whilst being cost-effective, delivering better healthcare value and quality and improving health outcome for concussion patients (7,19). This necessitates fundamental changes to the healthcare system in concussion care to facilitate the delivery of best-practice outpatient care and improve quality of care (20). This consensus statement addresses the healthcare structure and processes in outpatient concussion care required to facilitate the delivery of best-practice care in specialist outpatient clinics to meet healthcare needs and improve patient outcome in the concussion population.

METHODS

The guidance on Conducting and REporting DElphi Studies was followed for reporting this consensus study (21).

The work was undertaken as part of wider work by the UK Head Injury Network. There was no direct involvement with patients or the public. The study did not meet the criteria for requiring ethical approval.

Study design

A modified Delphi approach was used for this study. We followed a five-stage process; (1) panel identification, (2) literature search for initial statement development, (3-4) two voting rounds followed by item removal and modifications and (5) consensus meeting and live voting to finalise the statements. The meeting was chaired by a working group member with previous experience in the Delphi consensus method.

Panel Selection

The UK Head Injury Network is a panel of clinical and academic experts with extensive experience in head injury care in England, and setup to improve access to and quality of concussion outpatient services – for sports-related and non-sports-related concussions – and provide a network to foster research collaborations. The network consists of a multidisciplinary panel from academia and clinical medicine with specialists in emergency medicine, neurology, neuropsychology, neurosurgery, neuroscience, paediatric medicine, rehabilitation medicine and sports and exercise medicine. Members are invited to join the UK

For peer review only - http://bmjopen.bmj.com/site/about/guidelines.xhtml

Head Injury Network if they meet the following criteria based on peer-identification (all members in the network were identified or recommended by existing members); (1) healthcare professionals with extensive first-hand clinical experience in concussions care in any care setting or specialty; and/or (2) academic expertise in concussions. The group work with Love of the Game, a national sports concussion charity, to achieve these goals.

All members of the UK Head Injury Network at the time of the consensus process were invited to vote and attend the final consensus meeting. The participating members were geographically diverse including representatives from a range of healthcare settings across England. In addition to the various specialties involved, this ensures a more representative and informed decision-making for the different stages and settings of care across England.

Evidence Review

The need for a consensus statement had been discussed in previous meetings and highlighted in work completed by the group (unpublished observations). As the field of concussion has several contentious issues, we took a wide approach to topics covered in the initial statement development.

As the literature on implementation science in concussion is scarce, the literature search provided the evidence for clinical tasks that could be implemented. The panel used this literature combined with their first-hand local experience in various clinical settings to provide guidance on the structures and processes required to feasibly deliver these clinical tasks in England. Thus, statement development was guided by a literature search, topics of discussion within the network and results from previous work. MEDLINE and Embase were searched for relevant literature in October 2022, primarily published national and international clinical guidelines and consensus statements (see supplementary material 1 for search strategy). The search was limited to literature published in English.

A subgroup of network members was responsible for this stage of the process (EK, PJH, AH). The initial statements were circulated to all network members for comments on the scope, structure and wording prior to starting the official rounds of voting.

For peer review only - http://bmjopen.bmj.com/site/about/guidelines.xhtml

Consensus Process

Two rounds of online surveys were administered between October and December 2022 followed by a virtual meeting in February 2023. The surveys were distributed for voting using the Qualtrics XM platform (Qualtrics, Provo, UT).

All voting was anonymous to ensure uninhibited voting. Participants were asked to vote on each statement and had the binary options agree-disagree. The cut-off threshold for agreement was set at ≥70% for the two rounds of voting (22–24). A statement was only included if agreement was above the pre-defined threshold for consensus. Free text comments were incorporated to allow members to suggest modifications or refinements of the statements. Statements that did not meet the threshold were excluded after each round of voting and statements were modified for further considerations if suggestions were made by the participants.

All participants were invited to a virtual meeting (Zoom Video Communications, San Jose, USA). The meeting was scheduled for 3 hours to provide sufficient time to discuss each statement in detail. The purpose of the meeting was to finalise the statements. Statements reaching agreement and disagreement were discussed where alternatives were considered for the latter kind. The threshold for agreement was raised to ≥85% for this meeting to ensure a robust degree of consensus. The final consensus statements were discussed and, where necessary, modifications were made to ensure clarity and accuracy before a final anonymous vote took place during the meeting.

Patient and Public Involvement

No patient involvement.

RESULTS

Evidence Review

Whilst several papers were identified on therapeutic interventions, educational interventions, and biomarkers for diagnosis and prognosis in concussion, as expected the literature on healthcare processes and structures in concussion is scarce. Thus, the final statements were primarily guided by previous discussions, interpretation of how research evidence on best-

For peer review only - http://bmjopen.bmj.com/site/about/guidelines.xhtml

practice could be formulated into the structure and process of the healthcare system, and research work by the network and patient focus group sessions (unpublished observation).

Key papers identified during the literature review included clinical practical guidelines on concussion management (25–28) and systematic reviews on therapeutic interventions (29–34), prognosis (35–44), recovery (45,46) and biomarkers (47,48). The clinical practice guidelines included a synthesis of practical guidelines primarily focused on four published papers; Center for Disease Control and Prevention Guideline on the Diagnosis and Management of Mild Traumatic Brain Injury Among Children (2019) (49), Consensus Statement on Concussion in Sport – the 5th International Conference on Concussion in Sport (2016), Ontario Neurotrauma Foundation Guideline for Concussion/Mild Traumatic Brain Injury & Persistent Symptoms (3rd edition; 2018), and Department of Veterans Affairs/Department of Defense Clinical Practice Guideline for the Management of Concussion-Mild Traumatic Brain Injury (2016) (26).

Whilst it was agreed that covering a wide range of topics was appropriate during the initial statement development, the statements in this consensus report were narrowed down to specifically focus on the delivery of healthcare provisions through processes and structures in concussion care. This was deemed to be the most appropriate and relevant due to (a) some disagreement on clinical practice and management issues in concussion among the panel, (b) determining that these statements were beyond the purpose of this group and some of this guidance is featured in the most recent international *Consensus Statement on Concussion in Sport (6th edition)* (50), and (c) recognition that the need for the healthcare service to be improved at a system level prior to changes in day-to-day clinical practice. These points became clear after the first round of voting and the majority of these statement were removed after this stage. The decision to remove the remaining statements on day-to-day practice was made during the final meeting.

Consensus Process

Table 1: Demographics for participating panel members

Characteristics	n (%)
Sex, female	6 (21%)

For peer review only - http://bmjopen.bmj.com/site/about/guidelines.xhtml

**Specialty**	
Emergency medicine	2 (7%)
Neurology	3 (11%)
Neuropsychiatry	1 (4%)
Neuropsychology	3 (11%)
Neurosurgery	12 (43%)
Paediatric medicine	1 (4%)
Rehabilitation medicine	1 (4%)
Neuroscience	2 (7%)
Sports and exercise medicine	2 (7%)
Other	1 (4%)
**Region**	
East Midlands	1 (4%)
East of England	5 (18%)
Greater London	3 (11%)
North East England	4 (14%)
North West England	5 (18%)
South East England	4 (14%)
South West England	3 (11%)
West Midlands	1 (4%)
Yorkshire and the Humber	2 (7%)

Both rounds of voting had 21 votes (75% of network members at the time; see table 1 for demographic information on participating members). Following the initial statement development, the first round of voting had 56 items (figure 1, supplementary material 2.1). 9

For peer review only

statements were eliminated after the first round of voting as they were below the threshold of agreement and 6 statements were amended based on comments from voting members. Of the 56 statements included in the first round of voting, 23 statements were included that did not follow the standard consensus methodology (supplementary material 2.2). A different process was used for these statements and contained single-select or multi-select multiple choice options rather than the binary agree-disagree format used for the consensus statements. These statements are an important part of understanding the structure and processes for service delivery. Based on the responses from the first round of voting, two of these statements were amended and added as consensus statements with the binary agree-disagree response options in the following round of voting. As a result, the second round of voting included 26 statements that were put to a vote (supplementary material 3). Three items did not reach the threshold of agreement following the second round of voting and were removed, leaving 23 statements to be discussed during the meeting. No statements were amended at this stage.

The virtual consensus meeting resulted in four statements being eliminated, two pairs of statements were combined and one statement was added. Of the eliminated statements, three were deemed to be beyond the scope of this consensus statement. The statement added during the meeting was suggested by one member and voted on twice as the level of agreement was just below the threshold following the first vote. The statement was modified and included following the second vote as the level of agreement was above the threshold.

Figure 1: Consensus process

This resulted in a final 18 statements (table 2), all above the raised threshold of 85% agreement. 14 statements achieved unanimous agreement. The final statements were organised into three themes; (1) care pathway to structured follow-up, (2) prognosis and measures of recovery, and (3) provision of outpatient clinics.

Table 2: Final consensus statements

	Agreement %
**1 Care Pathway to Structured Follow-up**	
Concussion patients with persistent symptoms should be able to access specialist clinics.	100%

For peer review only

There should be dedicated concussion clinics, or where not possible, clinics with dedicated time for concussion patients.	100%
Specialist head injury clinics should comprise a multidisciplinary team that should include, as a minimum, a clinician, neuropsychologist and vestibular physiotherapist.	100%
There should be a direct patient pathway for patients with persistent symptoms to specialist outpatient services from the ED, GP and other sources.	100%
**2 Prognosis and Measures of Recovery**	
There should be a system for patients to be triaged to a specialist in head injury clinics based on persistent symptoms.	100%
There may be a role for digital tools (e.g., platforms, apps) to be used for screening for persistent symptoms following concussion.	100%
Symptom scales can aid the assessment of symptom burden.	100%
Screening for persistent symptoms can be aided by symptom scales.	100%
A new patient-reported tool for the assessment of symptoms and outcome should be developed and validated.	90%
Prolonged symptoms may develop even in the absence of specific acute signs, such as loss of consciousness, post-traumatic amnesia and vomiting.	100%
There are no prognostic tools currently available with sufficient clinical accuracy to predict the risk of developing persistent symptoms.	100%

For peer review only - http://bmjopen.bmj.com/site/about/guidelines.xhtml

There is currently insufficient evidence to support the clinical application of objective biomarkers (i.e., fluid, imaging) for identifying and screening for persistent symptoms after a concussion.	100%
Symptomatic patients with abnormal cranial/brain imaging should be considered for follow-up in a specialist clinic.	89%
There is not yet sufficient evidence to support the routine use of advanced imaging techniques (e.g., DTI, fMRI, MEG) in clinical practice.	90%
**Provision of Outpatient Clinics**	
All patients should be directed to online information on concussion to help with expectations and recommendations.	100%
All clinics should have access to standardised protocols for assessing pituitary dysfunction.	100%
Co-morbidities, socioeconomic status and social support should be factored into clinical decision-making on management.	90%
The underlying cause(s) of persistent symptoms should be established and treated on a case-by-case basis.	100%

DTI, Diffusion Tensor Imaging; ED, Emergency Department; fMRI, functional Magnetic Resonance Imaging; GP, General Practice; MEG, Magnetoencephalography

DISCUSSION

Healthcare services for concussion patients need to be transformed to advance quality of care and patient outcome. Current healthcare services fail to meet the needs of a significant number of concussion patients which results in suboptimal recovery and failure of the healthcare system to fulfil its purpose of improving quality of life through enhancing health. There is an urgent need to improve the delivery of and access to concussion care in order to

For peer review only

For peer review only - http://bmjopen.bmj.com/site/about/guidelines.xhtml

reduce healthcare inequalities and errors in management. Guidelines exist, but healthcare services need to restructure so to facilitate the delivery of these guidelines.

The recommendations in this consensus statement primarily focus on the structure and process of specialist healthcare services in concussion care with the purpose of improving healthcare quality and patient outcome (20). These are the components in healthcare services that facilitate the implementation and delivery of best-practice care to achieve optimal health for the target patient population (51). Ultimately, patient outcome is determined by the structure and process of care which describe the care context and the sum of actions in the delivery of care, respectively (52). This consensus statement prioritises a patient-centric approach with a focus on timely, integrated, effective, efficient and evidence-based care and urge standardisation of these practices nationally.

Given the recent developments in the field, translating basic and clinical evidence into the healthcare system requires work on feasibility, implementation and service redesign (53,54). Without a systematic approach, the promise and desired goal of improving patient outcomes can be challenging to fulfill. Simultaneously, there are still gaps in evidence. Consensus statements serve as an important tool in bridging areas where evidence is under-developed (55). This consensus statement provides guidance outlines how to redesign the structures and processes in care delivery for concussion patients in England. This is through expert opinion based on the current level of evidence and first-hand local experience in the various clinical settings which concussion patients encounter and academic expertise. The guidance needs to be updated as the evidence changes, but this ensures patient outcome to improve without delay.

The statements included in the first voting round that did not follow the consensus format will help guide system redesign in future work through providing further detailed guidance on fundamental factors in concussion healthcare delivery. This includes the structure of clinical teams, timelines for care tasks and other processes in the clinical pathway from injury to specialist outpatient services.

Whilst the consensus statements are aimed at all concussions, sports-related and non-sports-related concussions, the implementation of these services into the National Health Services in England requires reallocation and reprioritisation of resources, as well as a restructuring of

For peer review only - http://bmjopen.bmj.com/site/about/guidelines.xhtml

the system. Applying these changes for the subgroup of concussions occurring in sports – at both professional and grassroot levels – could be more practical and achievable at this stage, especially accounting for the high number of all-cause concussions. For example, direct patient pathways from local sports clubs could feasibly be implemented with quick-access clinics given the manageable number of sports-related concussions. A short-term goal of the UK Head Injury Network is the development of regional sports concussion clinics across the country to alleviate the burden of concussions in all levels of sports. These clinics aim to provide evidence-based care. However, it is intended that these services will ultimately be expanded and offered to all-cause concussion patients. Thus, while the implementation of these statements will primarily change clinical services for sports-related concussions in the near future, the statements are directed at national health services offered to all patients in the longer term.

Clinical Implications

Care Pathway to structured follow-up

Patients with persistent symptoms require further medical attention following the initial medical review– which is typically performed in emergency departments, general practice surgeries or by sport physicians – to recover to their pre-injury level of health and function. Yet, in a CENTER-TBI study, 90% of 71 neurotrauma centres involved in the Europe-based study do not routinely schedule follow-up appointments for head injury patients discharged from the emergency department (56).

In the UK, there is no care pathway to ensure access to structured follow-up which is likely a fundamental underlying cause for the unmet needs in concussion care. The absence of such a service may be attributed to the heterogeneity of concussions and the vast differences in patient needs. However, a flexible and multidisciplinary approach is needed to personalise care and meet patient needs across the heterogenous recovery trajectories in the concussion population. Evidence suggests that providing multidisciplinary interventions can improve return to work and quality of life in this patient population (57). Appropriate resources – time, equipment and staffing – need to be allocated to these services to ensure there is capacity to meet the diversity and scale of need. As a minimum, we suggest the inclusion of, though not limited to, a clinician (i.e. specialist nurse, neurologist, neurosurgeon, sport and exercise physician), neuropsychologist and vestibular physiotherapist. This is due to the

For peer review only - http://bmjopen.bmj.com/site/about/guidelines.xhtml

incidence of neurological, cognitive, psychological, cervicogenic and vestibular symptoms in post-concussive patients (58–60). Moreover, due to the knowledge and skillset of physiotherapists, it may be beneficial to develop a new subspecialty in 'concussion physiotherapy' (28,61).

Providing timely care is fundamental to meet patients' health needs. Delaying intervention can prolong recovery further and result in poorer outcome. One study found patients who sought care more than 30 days after a concussion had worse outcome at three months compared with patients who sought care earlier (62). Concussion services should be redesigned with care pathways that offer access to structured follow-up for all concussion patients as necessary to ensure these patients receive timely and appropriate care. These statements echo the recommendations in the recent Lancet Commission on traumatic brain injury (63).

Prognosis and Measures of Recovery

Objective measures of disease and prognostic factors provide a means for directing care (64). One of the greatest challenges in concussion management is the lack of objective markers and the absence of reliable prognostic factors for recovery. Thus, early identification of patients at risk of prolonged recovery is not possible and therefore assessing the need for follow-up for individuals at an early stage is challenging. Consequently, no predictive tool can be used reliably in the clinical setting to assess the risk of prolonged recovery and the need for follow-up in the outpatient setting (40). Even patients without marked acute signs and symptoms are at risk of prolonged recovery and should not be dismissed inadvertently. Of note, the panel recommends that patients with abnormal scans during the acute phase should always be offered follow-up as there is significant observable pathology during the acute phase to warrant further medical assessment (65). There is evidence to suggest that some gross structural abnormalities found on acute CT imaging may be associated with worse outcomes (66). However, we suggest that anything beyond standard structural imaging modalities – to rule out other pathologies – should not be used in the clinical setting. Whilst advanced imaging methods are showing promise in research, they are yet to demonstrate reliable clinical utility and we do not recommend using these tools in clinical practice (38,47,48).

For peer review only - http://bmjopen.bmj.com/site/about/guidelines.xhtml

In the absence of validated objective measures, ongoing self-reporting is currently the most reliable primary indicator of health status and method for identification of patients with persistent symptoms (67). Of note, non-disclosure of concussion symptoms is a concern that needs to be addressed and is particularly prevalent in professional and community sports (68,69). To filter out people who have recovered and identify individuals who need further medical review, we recommend a screening system. This would need to be cost-effective and scalable due to the size of the concussion population. We suggest digital tools as a platform for the screening system as they offer a remote, convenient, efficient and scalable solution (70). Once set up, costs remain comparatively low whilst also overcoming traditional barriers related to geographical, financial and resource limitations (71,72). Such a system could also be efficiently and effectively used to triage patients to the most appropriate clinician in the multidisciplinary clinic based on patient-reported symptoms and disabilities.

Standardising evaluation of recovery in the absence of objective markers is challenging. However, symptom scales may aid assessment of symptom burden and provide a means for screening patients, particularly as history taking and patient-reported symptoms are currently the only methods for measuring recovery (67). To achieve this long-term, we recommend developing and validating a patient-reported outcome measure for the concussion population to help assess recovery and determine care needs. This may include assessment of symptom burden, functional status, return to work or activity and quality of life. However, the specifics need to be determined in an appropriate study. While this study focuses on clinically-actionable statements, we believe this is a research priority that is important to include as it is required to achieve many of the clinical statements recommended. A validated patient-reported outcome measure will facilitate the clinical pathway for concussion patients to specialist outpatient services and will help move the field forward.

In the era of patient-centric and value-based healthcare, patient-reported outcome measures are increasingly important to capture the patient's perspective of their health. Even when objective measures are identified in the future, these tools should be utilised to capture a holistic patient-centric assessment of health status and recovery.

Provision of Outpatient Clinics

For peer review only - http://bmjopen.bmj.com/site/about/guidelines.xhtml

Providing early information and awareness is essential to managing expectations and can enhance recovery. A TRACK-TBI study found that only 42% of patients reported receiving head injury information at discharge from the emergency department (73). Providing proper discharge information can be challenging in the emergency department due to the busy environment in these clinical settings and the cognitive state of the patients initially after injury which may prevent them from comprehending and retaining information (74). Thus, we suggest directing patients to a single online platform for evidence-based information. The online modality allows anyone to access the information at any time, provides the same information to all regardless of geographical region and is easier to keep up to date with the evidence, which is particularly important in a fast-evolving field.

For patients with persistent symptoms, determining the most appropriate therapy can be challenging, especially in the absence of sufficiently strong evidence on therapeutic efficacy and timing of interventions. Given the vast heterogeneity in cases, no single treatment plan can be utilised for all concussion patients and clinicians must evaluate the most appropriate intervention(s) on a case-by-case basis. Whilst further research is required in this domain some therapeutic interventions have shown promise. Recommendations for therapeutic approaches are beyond the scope of this consensus statement and we recommend referring to other literature on this topic (29–34). Biopsychosocial factors should be considered when assessing clinical and social care needs as these have been shown consistently to influence patient recovery (41).

Lastly, we suggest that concussion clinics should have access to a standardised protocol for assessing pituitary function. Pituitary dysfunction can result in a range of different symptoms, many of which overlap with concussion symptoms, including fatigue, anxiety and mood disturbances. The literature on the incidence of pituitary dysfunction after concussions and screening protocols is still an area of active research. We suggest referring to a guidance paper by the British Neurotrauma Group on screening and management of pituitary dysfunction after traumatic brain injury (75).

Recent Developments in Sports Concussion Guidelines

The 6th edition of the consensus statement on concussion in sport were published this year (50). This statement also advises that individuals with persistent symptoms after a concussion (suggested as symptom persisting for longer than 4 weeks) should undergo a multimodal

For peer review only - http://bmjopen.bmj.com/site/about/guidelines.xhtml
For peer review only

evaluation, including the use of standardised, validated symptom scales for assessment and to guide referrals. The statement recommends referral to specialist clinicians ideally part of a multidisciplinary network of practitioners, where possible, for targeted treatment for persistent symptoms including *cervicogenic symptoms, migraine and headache, cognitive and psychological difficulties, balance disturbances, vestibular signs and oculomotor manifestations*. Regarding the multidisciplinary network, the statement proposes the inclusion of *sports medicine physicians, athletic trainers/therapists, physiotherapists, occupational therapists, sports chiropractors, neurologists, neurosurgeons, neuropsychologists, ophthalmologists, optometrists, physiatrists, psychologists and psychiatrists.* Furthermore, biopsychosocial and co-morbidities should also be considered in the context of persistent symptoms after concussions. Additionally, similar guidance has been provided regarding the use of advanced neuroimaging and other objective biomarkers in clinical practice.

Limitations

The literature on concussion, though rapidly evolving, is contentious and there are still numerous areas of disagreement amongst the panel. As discussed above, a wide approach was taken for the initial statement development which was later narrowed to focus on the healthcare structure and processes, in part. It cannot be excluded that some disagreement on statements was in part due to differences in definitions and terminology use despite probable broad agreement on the statement in principle. However, establishing agreement on terminology is a different consensus process on its own and was beyond the scope of this study.

We engaged with different stakeholders including academics, clinicians from different specialties and charities, and drew from previous patient-focus group sessions (unpublished observations). To ensure more robust statements, we chose a high threshold for consensus. Whilst these statements reached agreement in this panel, we cannot exclude the possibility or to what degree the results are influenced by the experience and expertise of these individuals both for the included and excluded statements. The number of participants was in line with recommendations for Delphi studies (76), however, more representatives from each specialty involved could have benefitted the consensus process (77). The study was also limited in female representation. However, due to the high level of agreement and consistency with the

For peer review only - http://bmjopen.bmj.com/site/about/guidelines.xhtml
For peer review only

literature, we believe the statements can be integrated reliably in the national healthcare system.

General practitioners are typically the first point of contact for patients with persistent problems seeking medical attention after a concussion and are a key player in the patient pathway. While the perspective of general practitioners is crucial for the wider concussion healthcare services, this study focused on the structures and processes in specialist outpatient services. However, the involvement of general practitioners is crucial for implementation and redesigning the concussion pathway.

CONCLUSION

This consensus statement outlines structural and process-based factors needed to move towards standardised best-practice care in concussion. This includes a final 18 statements on the care pathway for structured follow-up, prognosis and measures of recovery and provision of outpatient clinics. The statements are aimed at both the sports-related and all-cause concussion populations, however, they should be implemented for sports-related concussions as the first step before expanding to all concussion patients given the greater feasibility of this approach. Further research and stakeholder engagement is needed to determine best-practice clinical concussion management in outpatient clinics and the recommendations in this statement provide the structures and processes whereby best-practice patient-centred care can be delivered in a timely, integrated, efficient and effective manner.

Acknowledgements

We would like to thank Love of the Game for their support with this work. Peter Hutchinson would also like to thank the NIHR Senior Investigator Award, Cambridge Biomedical Research Centre, Medtech Brain Injury Co-operative, Global Health Research Group on Acquired Brain and Spine Injury and Royal College of Surgeons of England. AH is supported by the Cambridge Biomedical Research Campus, the Royal College of Surgeons of England, and is a Theme Lead in the NIHR Brain Injury MedTech Collaborative.

Contributorship statement

All authors have made substantial contributions to the manuscript in the following ways: EK, AH, AK, MP and PJAH led the delivery and planning of this consensus statement. AB, CG, MGre, MGri, TG, PG, AH, DH, PJAH, PJ, BJ, AK, EK, TL, TM, CH, SM, JN, RN, VR, KS,

For peer review only - http://bmjopen.bmj.com/site/about/guidelines.xhtml
For peer review only

RS, DT, PW, MW, EW were responsible for the conception and design of the study. AK moderated the consensus meeting. AB, CG, MGre, MGri, TG, PG, AH, DH, PJAH, PJ, BJ, AK, EK, TL, TM, CH, SM, JN, RN, VR, KS, RS, DT, PW, MW, EW participated in the acquisition, analysis and interpretation of data. EK drafted the manuscript and CG, MGa, TG, AH, PJAH, PJ, BJ, AK, EK, CH, RN, DT, PW, and EW revised it thoroughly. All authors gave final approval of the submitted manuscript version.

Competing interests

AB serves as an expert advisor on concussion for England's Rugby Football Union, Football Association and Premier League. He also consults and has stocks in Marker Diagnostics LTD and licenses in Salivary Biomarker of Concussion. AH served on the NICE Guideline Committee Trauma Guidelines for 2023. BJ consults for Premiership Rugby, London, and Rugby Football League, Manchester. DT consults for the Leeds Rhinos Rugby League Club and Hull City Tigers Football Club. MG serves on the advisory board for the Players Football Association. RN has undertaken work as a medicolegal expert in sports concussions.
RS has served as an independent expert in legal cases in which head injury has occurred, some of these were mild head injuries. He also has received funding from the Rugby Football Association and Football Association for the Advanced Brainhealth project. TG has advised on legal cases where head injury has occurred, some of which were mild head injuries.

Funding

No funding was received for this work.

Data availability statement

Data are available upon reasonable request.

Ethical statement

The study was exempt from requiring ethical approval in accordance with the University of Cambridge Policy on the Ethics of Research Involving Human Participants and Personal Data.

REFERENCES

For peer review only

1. National Institute for Health and Care Excellence. Head injury: assessment and early management [online]. [London]: NICE; 2023 [cited 2023 October 5]. (NICE guideline [NG232]). Available from https://www.nice.org.uk/guidance/ng232
2. Silverberg ND, Iverson GL, Cogan A, Dams-O-Connor K, Delmonico R, Graf MJP, et al. The American Congress of Rehabilitation Medicine Diagnostic Criteria for Mild Traumatic Brain Injury. Arch Phys Med Rehabil. 2023 Aug;104(8):1343–55.
3. Sharp DJ, Jenkins PO. Concussion is confusing us all. Pract Neurol [Internet]. 2015;15(3):172–86. Available from: https://www.scopus.com/inward/record.uri?eid=2-s2.0-84929330344&doi=10.1136%2fpractneurol-2015-001087&partnerID=40&md5=c2ae641c33158dcbe06dc0b311be793f
4. Ganau L, Ligarotti GKI, Prisco L, Ganau M. Letter: A Scoping Review of Registered Clinical Studies on Mild Traumatic Brain Injury and Concussion (2000-2019). Neurosurgery. 2021 Sep;89(3):E178–9.
5. Levin HS, Diaz-Arrastia RR. Diagnosis, prognosis, and clinical management of mild traumatic brain injury. Lancet Neurol. 2015 May;14(5):506–17.
6. Marshall SW, Guskiewicz KM, Shankar V, McCrea M, Cantu RC. Epidemiology of sports-related concussion in seven US high school and collegiate sports. Inj Epidemiol. 2015;2(1).
7. Langer L, Levy C, Bayley M. Increasing Incidence of Concussion: True Epidemic or Better Recognition? Journal of Head Trauma Rehabilitation. 2020 Jan;35(1):E60–6.
8. McCrea M, Guskiewicz KM, Marshall SW, Barr W, Randolph C, Cantu RC, et al. Acute Effects and Recovery Time Following Concussion in Collegiate Football Players. JAMA. 2003 Nov 19;290(19):2556.
9. McCrory P, Meeuwisse WH, Echemendia RJ, Iverson GL, Dvořák J, Kutcher JS. What is the lowest threshold to make a diagnosis of concussion? Br J Sports Med. 2013 Apr 11;47(5):268–71.
10. Cancelliere C, Verville L, Stubbs JL, Yu H, Hincapié CA, Cassidy JD, et al. Post-Concussion Symptoms and Disability in Adults With Mild Traumatic Brain Injury: A Systematic Review and Meta-Analysis. Vol. 40, Journal of Neurotrauma. 2023.
11. Stern RA, Seichepine D, Tschoe C, Fritts NG, Alosco ML, Berkowitz O, et al. Concussion Care Practices and Utilization of Evidence-Based Guidelines in the Evaluation and Management of Concussion: A Survey of New England Emergency Departments. Vol. 34, Journal of Neurotrauma. 2017.

For peer review only - http://bmjopen.bmj.com/site/about/guidelines.xhtml

12. Ross J, Santhirapala R, MacEwen C, Coulter A. Helping patients choose wisely. Vol. 361, BMJ (Clinical research ed.). 2018.
13. Maughan D, Ansell J. Protecting resources, promoting value: a doctors guide to cutting waste in clinical care. Vol. 24, Academy of Medical Royal Colleges. 2014.
14. Williams E, Buck D, Babalola G. What are health inequalities? [online]. [London]: The King's Fund; 2020 [updated 2022 June; cited 2023 Oct 5]. Available from https://www.kingsfund.org.uk/publications/what-are-health-inequalities
15. Dasic D, Morgan L, Panezai A, Syrmos N, Ligarotti GKI, Zaed I, et al. A scoping review on the challenges, improvement programs, and relevant output metrics for neurotrauma services in major trauma centers. Surg Neurol Int. 2022 Apr 29;13:171.
16. National Institute for Health and Excellence. Practical steps to improving the quality of care and services using NICE guidance [Internet]. 2018 [cited 2023 Oct 4]. Available from: https://www.nice.org.uk/Media/Default/About/what-we-do/Into-practice/Practical-steps-improving-quality-of-care-and-services-using-NICE-guidance.pdf
17. Wiebe DJ, Comstock RD, Nance ML. Concussion research: a public health priority. Injury Prevention. 2011 Feb 1;17(1):69–70.
18. Department for Culture M and SUG. UK Concussion Guidelines for Non-Elite (Grassroots) Sport [Internet]. 2023 [cited 2023 Sep 25]. Available from: http://sramedia.s3.amazonaws.com/media/documents/9ced1e1a-5d3b-4871-9209-bff4b2575b46.pdf
19. Veliz P, McCabe SE, Eckner JT, Schulenberg JE. Trends in the Prevalence of Concussion Reported by US Adolescents, 2016-2020. JAMA. 2021 May 4;325(17):1789.
20. Hannawa AF, Wu AW, Kolyada A, Potemkina A, Donaldson LJ. The aspects of healthcare quality that are important to health professionals and patients: A qualitative study. Patient Educ Couns. 2022;105(6).
21. Jünger S, Payne SA, Brine J, Radbruch L, Brearley SG. Guidance on Conducting and REporting DElphi Studies (CREDES) in palliative care: Recommendations based on a methodological systematic review. Vol. 31, Palliative Medicine. 2017.
22. Diamond IR, Grant RC, Feldman BM, Pencharz PB, Ling SC, Moore AM, et al. Defining consensus: A systematic review recommends methodologic criteria for reporting of Delphi studies. J Clin Epidemiol. 2014 Apr;67(4):401–9.

For peer review only - http://bmjopen.bmj.com/site/about/guidelines.xhtml

23. Slade SC, Dionne CE, Underwood M, Buchbinder R. Standardised method for reporting exercise programmes: protocol for a modified Delphi study. BMJ Open. 2014 Dec 30;4(12):e006682.
24. Vogel C, Zwolinsky S, Griffiths C, Hobbs M, Henderson E, Wilkins E. A Delphi study to build consensus on the definition and use of big data in obesity research. Int J Obes. 2019 Dec 17;43(12):2573–86.
25. Giza CC, Kutcher JS, Ashwal S, Barth J, Getchius TSD, Gioia GA, et al. Summary of evidence-based guideline update: Evaluation and management of concussion in sports: Report of the Guideline Development Subcommittee of the American Academy of Neurology. Neurology. 2013 Jun 11;80(24):2250–7.
26. Silverberg ND, Iaccarino MA, Panenka WJ, Iverson GL, McCulloch KL, Dams-O'Connor K, et al. Management of Concussion and Mild Traumatic Brain Injury: A Synthesis of Practice Guidelines. Arch Phys Med Rehabil. 2020 Feb;101(2):382–93.
27. Marshall S, Bayley M, McCullagh S, Velikonja D, Berrigan L, Ouchterlony D, et al. Updated clinical practice guidelines for concussion/mild traumatic brain injury and persistent symptoms. Brain Inj. 2015 May 12;29(6):688–700.
28. Quatman-Yates CC, Hunter-Giordano A, Shimamura KK, Landel R, Alsalaheen BA, Hanke TA, et al. Physical Therapy Evaluation and Treatment After Concussion/Mild Traumatic Brain Injury. Journal of Orthopaedic & Sports Physical Therapy. 2020 Apr;50(4):CPG1–73.
29. Boussard CN de, Holm LW, Cancelliere C, Godbolt AK, Boyle E, Stålnacke BM, et al. Nonsurgical Interventions After Mild Traumatic Brain Injury: A Systematic Review. Results of the International Collaboration on Mild Traumatic Brain Injury Prognosis. Arch Phys Med Rehabil. 2014 Mar;95(3):S257–64.
30. Schneider KJ, Leddy JJ, Guskiewicz KM, Seifert T, McCrea M, Silverberg ND, et al. Rest and treatment/rehabilitation following sport-related concussion: a systematic review. Br J Sports Med. 2017 Jun;51(12):930–4.
31. Murray DA, Meldrum D, Lennon O. Can vestibular rehabilitation exercises help patients with concussion? A systematic review of efficacy, prescription and progression patterns. Br J Sports Med. 2017 Mar;51(5):442–51.
32. Lal A, Kolakowsky-Hayner SA, Ghajar J, Balamane M. The Effect of Physical Exercise After a Concussion: A Systematic Review and Meta-analysis. Am J Sports Med. 2018 Mar 1;46(3):743–52.

For peer review only - http://bmjopen.bmj.com/site/about/guidelines.xhtml

33. Reid SA, Farbenblum J, McLeod S. Do physical interventions improve outcomes following concussion: a systematic review and meta-analysis? Br J Sports Med. 2022 Mar;56(5):292–8.

34. Heslot C, Azouvi P, Perdrieau V, Granger A, Lefèvre-Dognin C, Cogné M. A Systematic Review of Treatments of Post-Concussion Symptoms. J Clin Med. 2022 Oct 21;11(20):6224.

35. Rausa VC, Anderson V, Babl FE, Takagi M. Predicting Concussion Recovery in Children and Adolescents in the Emergency Department. Curr Neurol Neurosci Rep. 2018 Nov 19;18(11):78.

36. Mercier E, Tardif PA, Cameron PA, Émond M, Moore L, Mitra B, et al. Prognostic value of neuron-specific enolase (NSE) for prediction of post-concussion symptoms following a mild traumatic brain injury: a systematic review. Brain Inj. 2018 Jan 2;32(1):29–40.

37. Zetterberg H, Morris HR, Hardy J, Blennow K. Update on fluid biomarkers for concussion. Concussion. 2016 Dec;1(3):CNC12.

38. McCrea M, Meier T, Huber D, Ptito A, Bigler E, Debert CT, et al. Role of advanced neuroimaging, fluid biomarkers and genetic testing in the assessment of sport-related concussion: a systematic review. Br J Sports Med. 2017 Jun;51(12):919–29.

39. O'Connell B, Kelly ÁM, Mockler D, Orešič M, Denvir K, Farrell G, et al. Use of Blood Biomarkers in the Assessment of Sports-Related Concussion—A Systematic Review in the Context of Their Biological Significance. Clinical Journal of Sport Medicine. 2018 Nov;28(6):561–71.

40. Iverson GL, Gardner AJ, Terry DP, Ponsford JL, Sills AK, Broshek DK, et al. Predictors of clinical recovery from concussion: a systematic review. Br J Sports Med. 2017 Jun 31;51(12):941–8.

41. van Ierssel J, Pennock KF, Sampson M, Zemek R, Caron JG. Which psychosocial factors are associated with return to sport following concussion? A systematic review. J Sport Health Sci. 2022 Jul;11(4):438–49.

42. Karantali E, Kazis D, McKenna J, Chatzikonstantinou S, Petridis F, Mavroudis I. Neurofilament light chain in patients with a concussion or head impacts: a systematic review and meta-analysis. European Journal of Trauma and Emergency Surgery. 2022 Jun 18;48(3):1555–67.

For peer review only

43. Silverberg ND, Gardner AJ, Brubacher JR, Panenka WJ, Li JJ, Iverson GL. Systematic Review of Multivariable Prognostic Models for Mild Traumatic Brain Injury. J Neurotrauma. 2015 Apr 15;32(8):517–26.

44. Cancelliere C, Hincapié CA, Keightley M, Godbolt AK, Côté P, Kristman VL, et al. Systematic Review of Prognosis and Return to Play After Sport Concussion: Results of the International Collaboration on Mild Traumatic Brain Injury Prognosis. Arch Phys Med Rehabil. 2014 Mar;95(3):S210–29.

45. Kamins J, Bigler E, Covassin T, Henry L, Kemp S, Leddy JJ, et al. What is the physiological time to recovery after concussion? A systematic review. Br J Sports Med. 2017 Jun;51(12):935–40.

46. Manley G, Gardner AJ, Schneider KJ, Guskiewicz KM, Bailes J, Cantu RC, et al. A systematic review of potential long-term effects of sport-related concussion. Br J Sports Med. 2017 Jun;51(12):969–77.

47. Lindsey HM, Hodges CB, Greer KM, Wilde EA, Merkley TL. Diffusion-Weighted Imaging in Mild Traumatic Brain Injury: A Systematic Review of the Literature. Neuropsychol Rev. 2023 Mar 15;33(1):42–121.

48. Khong E, Odenwald N, Hashim E, Cusimano MD. Diffusion Tensor Imaging Findings in Post-Concussion Syndrome Patients after Mild Traumatic Brain Injury: A Systematic Review. Front Neurol. 2016 Sep 19;7.

49. Lumba-Brown A, Yeates KO, Sarmiento K, Breiding MJ, Haegerich TM, Gioia GA, et al. Centers for Disease Control and Prevention Guideline on the Diagnosis and Management of Mild Traumatic Brain Injury Among Children. JAMA Pediatr. 2018 Nov 5;172(11):e182853.

50. Patricios JS, Schneider KJ, Dvorak J, Ahmed OH, Blauwet C, Cantu RC, et al. Consensus statement on concussion in sport: the 6th International Conference on Concussion in Sport-Amsterdam, October 2022. Br J Sports Med. 2023;57(11).

51. Rademakers J, Delnoij D, De Boer D. Structure, process or outcome: Which contributes most to patients' overall assessment of healthcare quality? BMJ Qual Saf. 2011;20(4).

52. NHS England and NHS Improvement. A model for measuring quality care [online]. [London]: NHS England; 2021. [cited 2023 Oct 5]. Available from: https://www.england.nhs.uk/wp-content/uploads/2022/02/qsir-measuring-quality-care.pdf

For peer review only

For peer review only - http://bmjopen.bmj.com/site/about/guidelines.xhtml

53. Grol R, Grimshaw J. From best evidence to best practice: Effective implementation of change in patients' care. Vol. 362, Lancet. 2003.

54. Bauer MS, Damschroder L, Hagedorn H, Smith J, Kilbourne AM. An introduction to implementation science for the non-specialist. BMC Psychol. 2015;3(1).

55. La Brooy C, Pratt B, Kelaher M. What is the role of consensus statements in a risk society? J Risk Res. 2020;23(5).

56. Foks KA, Cnossen MC, Dippel DWJ, Maas AIR, Menon D, van der Naalt J, et al. Management of Mild Traumatic Brain Injury at the Emergency Department and Hospital Admission in Europe: A Survey of 71 Neurotrauma Centers Participating in the CENTER-TBI Study. J Neurotrauma. 2017 Sep;34(17):2529–35.

57. Jensen KL, Wachner LG, Storm van den Brekel J, Hardis H, Smaakjær P, Hansen M V., et al. Multidisciplinary interventions increase weekly working hours and quality of life in persons with post-concussion syndrome. Neurol Res. 2023 Oct 3;45(10):926–35.

58. Hiploylee C, Dufort PA, Davis HS, Wennberg RA, Tartaglia MC, Mikulis D, et al. Longitudinal study of postconcussion syndrome: Not everyone recovers. J Neurotrauma [Internet]. 2017;34(8):1511–23. Available from: http://www.liebertonline.com/neu

59. Polinder S, Cnossen MC, Real RGL, Covic A, Gorbunova A, Voormolen DC, et al. A Multidimensional Approach to Post-concussion Symptoms in Mild Traumatic Brain Injury. Front Neurol [Internet]. 2018;9. Available from: https://www.scopus.com/inward/record.uri?eid=2-s2.0-85063738495&doi=10.3389%2ffneur.2018.01113&partnerID=40&md5=7da2daff1fa8712a1dffea114975fb89

60. Faulkner JW, Theadom A, Snell DL, Williams MN. Network analysis applied to post-concussion symptoms in two mild traumatic brain injury samples. Front Neurol. 2023 Jul 20;14.

61. Brown L, Camarinos J. The Role of Physical Therapy in Concussion Rehabilitation. Semin Pediatr Neurol [Internet]. 2019;30:68–78. Available from: https://www.scopus.com/inward/record.uri?eid=2-s2.0-85067548442&doi=10.1016%2fj.spen.2019.03.011&partnerID=40&md5=f17daf40e7be68cbd35160ee02771504

For peer review only - http://bmjopen.bmj.com/site/about/guidelines.xhtml

62. Bunt SC, LoBue C, Hynan LS, Didehbani N, Stokes M, Miller SM, et al. Early vs. delayed evaluation and persisting concussion symptoms during recovery in adults. Clin Neuropsychol. 2022 Sep 9;1–18.

63. Maas AIR, Menon DK, Manley GT, Abrams M, Åkerlund C, Andelic N, et al. Traumatic brain injury: progress and challenges in prevention, clinical care, and research. Lancet Neurol. 2022 Nov;21(11):1004–60.

64. Déry J, De Guise É, Bussières ÈL, Lamontagne ME. Prognostic factors for persistent symptoms in adults with mild traumatic brain injury: protocol for an overview of systematic reviews. Syst Rev. 2021;10(1).

65. Voormolen DC, Haagsma JA, Polinder S, Maas AIR, Steyerberg EW, Vuleković P, et al. Post-concussion symptoms in complicated vs. Uncomplicated mild traumatic brain injury patients at three and six months post-injury: Results from the CENTER-TBI study. J Clin Med. 2019;8(11).

66. Yuh EL, Jain S, Sun X, Pisică D, Harris MH, Taylor SR, et al. Pathological Computed Tomography Features Associated with Adverse Outcomes after Mild Traumatic Brain Injury: A TRACK-TBI Study with External Validation in CENTER-TBI. JAMA Neurol. 2021;78(9).

67. Yeates KO, Räisänen AM, Premji Z, Debert CT, Frémont P, Hinds S, et al. What tests and measures accurately diagnose persisting post-concussive symptoms in children, adolescents and adults following sport-related concussion? A systematic review. Vol. 57, British Journal of Sports Medicine. 2023.

68. Longworth T, McDonald A, Cunningham C, Khan H, Fitzpatrick J. Do rugby league players under-report concussion symptoms? A cross-sectional study of elite teams based in Australia. BMJ Open Sport Exerc Med. 2021 Jan 19;7(1):e000860.

69. Tadmor D, Till K, Phillips G, Brown J, Fairbank L, Hendricks S, et al. I won't let you down; why 20% of Men's and Women's Super League players underreported suspected concussions. J Sci Med Sport. 2023 Sep;

70. NIHR Evidence. What is digital health technology and what can it do for me? [online]. [London]. NIHR Evidence; 2022 [cited 2023 Oct 5]. Available from: https://evidence.nihr.ac.uk/collection/what-is-digital-health-technology/

71. WHO Team: Digital Health and Innovation GRCS and RH and RH and IGRCS and RH and R. Recommendations on digital interventions for health system strengthening [Online]. WHO; 2019 [cited 2023 Oct 5]. Available from:

For peer review only - http://bmjopen.bmj.com/site/about/guidelines.xhtml

https://iris.who.int/bitstream/handle/10665/311941/9789241550505-eng.pdf?sequence=31

72. Powell D, Stuart S, Godfrey A. Sports related concussion: an emerging era in digital sports technology. NPJ Digit Med [Internet]. 2021;4(1) (no pagination). Available from: https://www.nature.com/npjdigitalmed/

73. Seabury SA, Gaudette É, Goldman DP, Markowitz AJ, Brooks J, McCrea MA, et al. Assessment of Follow-up Care After Emergency Department Presentation for Mild Traumatic Brain Injury and Concussion. JAMA Netw Open. 2018 May 25;1(1):e180210.

74. Bergman K, Louis S. Discharge Instructions for Concussion: Are We Meeting the Patient Needs? Journal of Trauma Nursing. 2016 Nov;23(6):327–33.

75. Tan CL, Alavi SA, Baldeweg SE, Belli A, Carson A, Feeney C, et al. The screening and management of pituitary dysfunction following traumatic brain injury in adults: British Neurotrauma Group guidance. J Neurol Neurosurg Psychiatry. 2017 Nov;88(11):971–81.

76. Birko S, Dove ES, Özdemir V, Dalal K. Evaluation of nine consensus indices in delphi foresight research and their dependency on delphi survey characteristics: A simulation study and debate on delphi design and interpretation. PLoS One. 2015;10(8).

77. de Villiers MR, de Villiers PJT, Kent AP. The Delphi technique in health sciences education research. Med Teach. 2005;27(7).

For peer review only

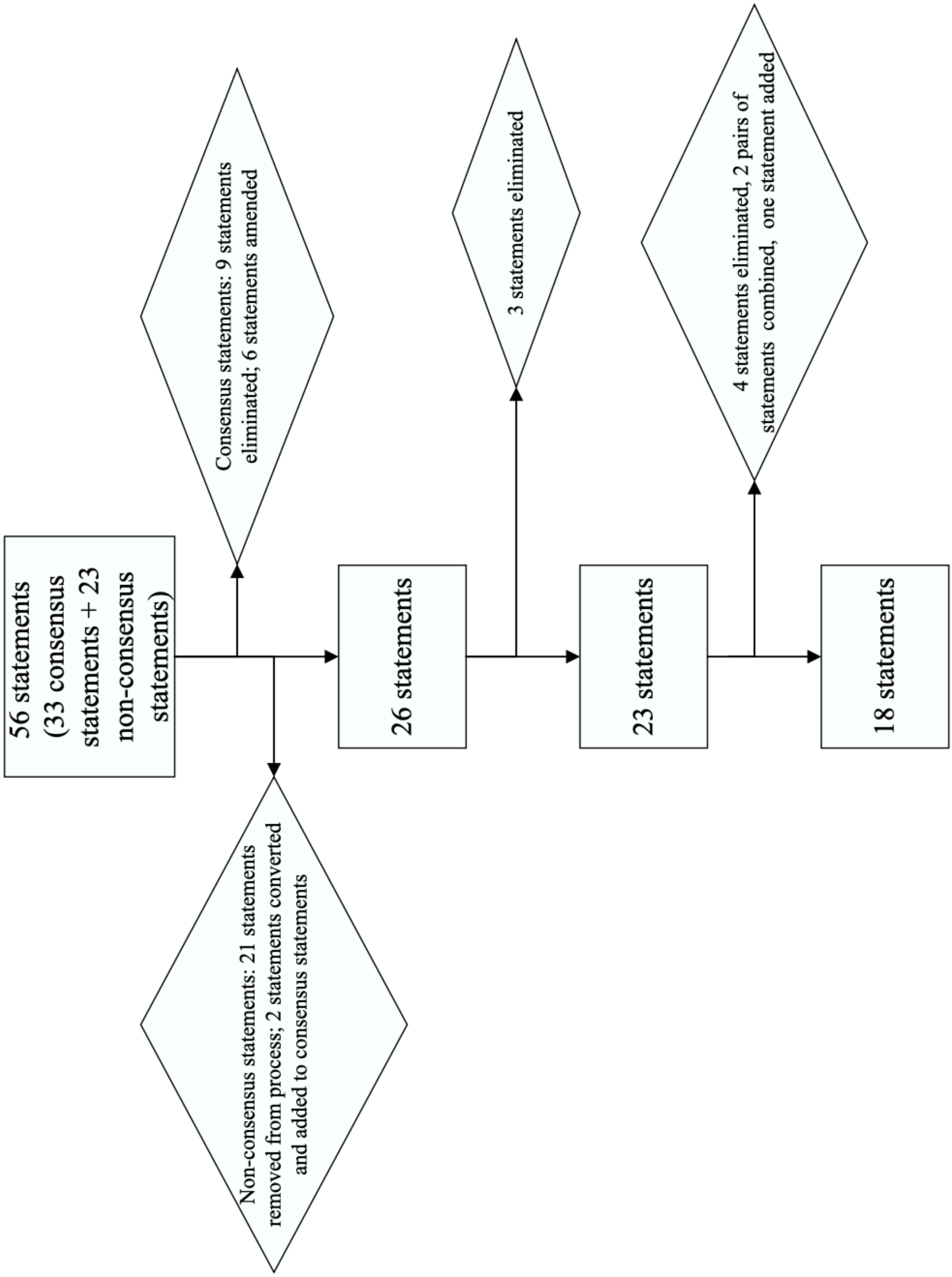
56 statements (33 consensus statements + 23 non-consensus statements)
Consensus statements: 9 statements eliminated; 6 statements amended
Non-consensus statements: 21 statements removed from process; 2 statements converted and added to consensus statements
26 statements
3 statements eliminated
23 statements
4 statements eliminated, 2 pairs of statements combined, one statement added
18 statements

Supplementary material 1: Literature search strategy

The search was conducted in MEDLINE and Embase. Below is the search used for Embase and the equivalent search was done for MEDLINE.

Embase search strategy

1 brain concussion/

2 postconcussion syndrome/

3 "mild traumatic brain injur*".mp. [mp=title, abstract, heading word, drug trade name, original title, device manufacturer, drug manufacturer, device trade name, keyword heading word, floating subheading word, candidate term word]

4 "concussion*".mp. [mp=title, abstract, heading word, drug trade name, original title, device manufacturer, drug manufacturer, device trade name, keyword heading word, floating subheading word, candidate term word]

5 "concussive*".mp. [mp=title, abstract, heading word, drug trade name, original title, device manufacturer, drug manufacturer, device trade name, keyword heading word, floating subheading word, candidate term word]

6 "mild head injur*".mp. [mp=title, abstract, heading word, drug trade name, original title, device manufacturer, drug manufacturer, device trade name, keyword heading word, floating subheading word, candidate term word]

7 "mild brain injur*".mp. [mp=title, abstract, heading word, drug trade name, original title, device manufacturer, drug manufacturer, device trade name, keyword heading word, floating subheading word, candidate term word]

8 "mtbi*".mp. [mp=title, abstract, heading word, drug trade name, original title, device manufacturer, drug manufacturer, device trade name, keyword heading word, floating subheading word, candidate term word]

9 "mild TBI*".mp. [mp=title, abstract, heading word, drug trade name, original title, device manufacturer, drug

For peer review only - http://bmjopen.bmj.com/site/about/guidelines.xhtml

manufacturer, device trade name, keyword heading word, floating subheading word, candidate term word]

10 "commotio cerebri".mp. [mp=title, abstract, heading word, drug trade name, original title, device manufacturer, drug manufacturer, device trade name, keyword heading word, floating subheading word, candidate term word]

11 "post-concussion symptom*".mp. [mp=title, abstract, heading word, drug trade name, original title, device manufacturer, drug manufacturer, device trade name, keyword heading word, floating subheading word, candidate term word]

12 "post-concussion syndrome*".mp. [mp=title, abstract, heading word, drug trade name, original title, device manufacturer, drug manufacturer, device trade name, keyword heading word, floating subheading word, candidate term word]

13 1 or 2 or 3 or 4 or 5 or 6 or 7 or 8 or 9 or 10 or 11 or 12

14 practice guideline/

15 clinical practice/

16 "guideline*".mp. [mp=title, abstract, heading word, drug trade name, original title, device manufacturer, drug manufacturer, device trade name, keyword heading word, floating subheading word, candidate term word]

17 "clinical practic*".mp. [mp=title, abstract, heading word, drug trade name, original title, device manufacturer, drug manufacturer, device trade name, keyword heading word, floating subheading word, candidate term word]

18 "recommend*".mp. [mp=title, abstract, heading word, drug trade name, original title, device manufacturer, drug manufacturer, device trade name, keyword heading word, floating subheading word, candidate term word]

19 "best practic*".mp. [mp=title, abstract, heading word, drug trade name, original title, device manufacturer, drug manufacturer, device trade name, keyword heading word, floating subheading word, candidate term word]

For peer review only - http://bmjopen.bmj.com/site/about/guidelines.xhtml

20 "clinical decision*".mp. [mp=title, abstract, heading word, drug trade name, original title, device manufacturer,
drug manufacturer, device trade name, keyword heading word, floating subheading word, candidate term word]
21 "clinical support*".mp. [mp=title, abstract, heading word, drug trade name, original title, device manufacturer,
drug manufacturer, device trade name, keyword heading word, floating subheading word, candidate term word]
22 "consensus".mp. [mp=title, abstract, heading word, drug trade name, original title, device manufacturer, drug
manufacturer, device trade name, keyword heading word, floating subheading word, candidate term word]
23 "position statement*".mp. [mp=title, abstract, heading word, drug trade name, original title, device
manufacturer, drug manufacturer, device trade name, keyword heading word, floating subheading word, candidate term word]
24 healthcare service.mp. or health service/
25 "*care system*".mp. [mp=title, abstract, heading word, drug trade name, original title, device manufacturer, drug
manufacturer, device trade name, keyword heading word, floating subheading word, candidate term word]
26 "service*".mp. [mp=title, abstract, heading word, drug trade name, original title, device manufacturer, drug
manufacturer, device trade name, keyword heading word, floating subheading word, candidate term word]
27 14 or 15 or 16 or 17 or 18 or 19 or 20 or 21 or 22 or 23 or 24 or 25 or 26 (3935187)
28 13 and 27

Supplementary material 2: Statements voting round 1

Supplementary material 2.1 Consensus Statement Voting Round 1

Statement	Agreement	Outcome
There should be a direct patient pathway from ED to specialist outpatient services available to	71%	None

For peer review only - http://bmjopen.bmj.com/site/about/guidelines.xhtml

all patients to ensure patients with persistent symptoms will be identified early.		
There is not sufficient evidence for injury-related events (i.e. presence of loss of consciousness, post-traumatic amnesia, vomiting) to predict prolonged symptoms.	71%	None
There is not sufficient evidence to support the clinical application of objective biomarkers (i.e. fluid, imaging) for identifying and screening for persistent symptoms after a mild TBI.	81%	None
There is not enough evidence to use prognostic tools to predict the risk of developing persistent symptoms.	81%	None
All mild TBI patients should be screened for persistent symptoms	67%	Eliminated
All mild TBI patients should receive follow-up in specialist clinics	19%	Eliminated
There should be a system for patients to be triaged to a specialist in (mild) TBI clinics based on persistent symptoms.	95%	Amended
All patients with history of 2 or more head injuries should be seen in specialist clinics regardless of whether they experience persistent symptoms.	29%	Eliminated
All patients with history of neurological conditions (i.e. migraines, epilepsy) should be seen in specialist clinics regardless of whether they experience persistent symptoms.	14%	Eliminated
All patients with history of psychological problems (i.e. depression, anxiety, PTSD) should be seen in specialist clinics regardless of whether they experience persistent symptoms.	10%	Eliminated

For peer review only - http://bmjopen.bmj.com/site/about/guidelines.xhtml

Only symptomatic patients should be seen in specialist clinics.	81%	Amended
Digital tools should be utilised to screen for persistent symptoms.	71%	Amended
Symptom scales are currently the most appropriate method available for measuring symptom burdens in patients.	71%	Amended
Symptom scales are currently the most appropriate method available to screen for persistent symptoms in patients.	71%	None
A bespoke symptom scale, such as the Rivermead or Post-Concussion Symptom Scale, should be used for screening symptoms.	80%	Amended
All patients should complete a symptom scale at the first point of contact with a clinician in the acute phase.	71%	None
Patients screening negative for PPCS on the survey at any point should be discharged from specialist service.	67%	Eliminated
Patients should be under the care of their regional mild TBI clinic until symptom resolution, and only discharged once fully recovered and returned to work, where applicable.	52%	Eliminated
The extended Glasgow Outcome Scale (eGOS) should be used to measure functional outcomes after mild TBI in routine clinical practice.	50%	Amended
The SF-36 or EQ-5D should be used to measure quality of life after mild TBI in routine clinical practice.	35%	Eliminated
A bespoke patient-reported outcome measure (PROM) should be developed for mild TBI and used in routine clinical practice	70%	None

For peer review only - http://bmjopen.bmj.com/site/about/guidelines.xhtml

There should be dedicated mild TBI clinics, or where not possible, TBI clinics with dedicated time to mild TBI patients.	100%	None
Specialist (mild) TBI clinics should comprise of a multidisciplinary team.	90%	None
All patients should be directed to a single bespoke online information portal on mild TBI to help with expectations and recommendations.	90%	None
Patients should be advised to rest (cognitive and physical rest) for 24-48 hours, after which they should gradually increase activity.	75%	None
Patients should be advised to rest (cognitive and physical rest) for as long as they are experiencing symptoms	25%	Eliminated
There is not sufficient evidence to support the use of advanced imaging techniques in clinical practice.	80%	None
There is not sufficient evidence to support screening for symptom subtypes. 10. Investigations and treatments should be symptom-based.	80%	Amended
All patients with persistent symptoms should complete a cognitive assessment reviewed by a neuropsychologist	-*	Amended
All clinics should have access to standardised protocols for assessing pituitary dysfunction.	90%	None
Adjusting expectation and education should be the primary initial approach when managing mild TBI, regardless of symptoms.	85%	None
Co-morbidities should be factored into clinical decision-making on management.	100%	None

For peer review only - http://bmjopen.bmj.com/site/about/guidelines.xhtml

Socioeconomic status and social support should be considered during clinical decision-making on management.	90%	None
Where patients are involved in sports, they should be advised to return to work or studies without symptom exacerbation before returning to unsupervised physical exercise.	70%	None
Low-intensity physical activity should be recommended as long as it does not exacerbate symptoms.	100%	None
Treatments should be assessed on a case-by-case basis.	-**	Amended

*The format of the response options for the statement was amended following voting and degree of agreement is therefore unavailable. **9 statements about symptom specific treatments were combined. The format of the response options for the statement was amended following voting and degree of agreement is therefore unavailable.

ED, Emergency Department; EQ-5D, EurQol-5 Dimensions; PPCS, Persistent Post-Concussion Symptoms; PTSD, Post Traumatic Stress Disorder; SF-36, Short-Form 36; TBI, Traumatic Brain Injury

Supplementary material 2.2 Additional statements

**Statements**	**Response options**
All patients should be screened for symptoms	2 weeks after injury<br>4 weeks after injury<br>6 weeks after injury<br>8 weeks after injury<br>10 weeks after injury<br>12 weeks after injury<br>All the above<br>Never<br>Other (open text)

For peer review only - http://bmjopen.bmj.com/site/about/guidelines.xhtml

Patients should be treated in primary care settings and only referred to specialist services if they do not improve within	2 weeks of injury<br>4 weeks of injury<br>6 weeks of injury<br>8 weeks of injury<br>10 weeks of injury<br>12 weeks of injury<br>Never<br>Other (open text)
Patients with persistent symptoms should first be reviewed in specialist clinic within	2 weeks of injury<br>4 weeks of injury<br>6 weeks of injury<br>8 weeks of injury<br>10 weeks of injury<br>12 weeks of injury<br>Never<br>Other (open text)
Patients should be followed-up in specialist clinic (in-person or telephone follow-up, depending on need)	Every 2 weeks until full recovery<br>Every 4 weeks until full recovery<br>Every 6 weeks until full recovery<br>Every 8 weeks until full recovery<br>Every 10 weeks until full recovery<br>Every 12 weeks until full recovery<br>None of the above, interval between reviews should increase with time as patients improve<br>None of the above, patients should only be reviewed once in clinic<br>Case-by-case assessment<br>Other (open text)
Criteria for clinic follow-up should be if they have	1 or more mild-severe symptoms<br>2 or more mild-severe symptoms<br>3 or more mild-severe symptoms

For peer review only

	1 or more moderate-severe symptoms<br>2 or more moderate-severe symptoms<br>3 or more moderate-severe symptoms.<br>None of the above (no set criteria) – symptom scales should always be interpreted by an experienced physician on a case-by-case basis<br>Other (open text)
The initial screening of patients should be done by	Automated system<br>Specialist nurse<br>Occupational therapist<br>Physical therapist<br>Neuropsychologist<br>Neurologist<br>Neurosurgeon<br>Neurorehabilitation specialist<br>Emergency medicine physician<br>Sport and exercise medicine physician<br>Other (open text)
The first review in specialist clinic should be done by	Automated system<br>Specialist nurse<br>Occupational therapist<br>Physical therapist<br>Neuropsychologist<br>Neurologist<br>Neurosurgeon<br>Neurorehabilitation specialist<br>Emergency medicine physician

For peer review only - http://bmjopen.bmj.com/site/about/guidelines.xhtml

	Sport and exercise medicine physician<br>Other (open text)
The first review in specialist clinic should be	Face-to-face<br>Telephone follow-up<br>Always depend on need based on the triage<br>None of the above<br>Other (open text)
New or worsening symptoms developed more than 1 month after injury should be investigated for non-head injury related causes under	The mild TBI specialist outpatient service.<br>Primary care services.<br>Other (open text)
A bespoke patient-reported outcome measure (PROM) should be developed for mild TBI and used in routine clinical practice	To measure functional outcomes<br>To measure quality of life<br>To measure symptom burden<br>All the above<br>None of the above<br>Other (open text)
At a minimum, the multidisciplinary team should consist of	Neurosurgeons<br>Neurologists<br>Neuropsychologist<br>Vestibular specialist<br>Physiotherapists<br>Specialist nurse<br>Occupational therapist<br>Neurorehabilitation specialist<br>Sport and exercise medicine physician<br>All the above<br>None of the above<br>Other (open text)

For peer review only

In addition to the core MDT, there should be a direct access to	Ophthalmologists<br>Sleep clinics<br>Headache/migraine specialist clinic<br>Internet-based Cognitive Behavioural Therapy<br>Community-level services<br>All the above<br>None of the above<br>Other (open text)
Patients with the following symptom(s) should have an MRI to exclude gross structural abnormalities.	Low mood<br>Anxiety<br>Memory problems<br>Concentration problems<br>Taking longer to think<br>Restlessness<br>Irritable<br>Feeling frustrated<br>Fatigue<br>Sleep disturbance<br>Nausea<br>Dizziness (without an obvious underlying vestibular pathology)<br>Headache<br>Sensitivity to noise<br>Sensitivity to light<br>Visual symptoms (without an obvious underlying visual pathology)<br>None of the above<br>All the above<br>Case-by-case assessment

For peer review only - http://bmjopen.bmj.com/site/about/guidelines.xhtml

All patients with persistent symptoms should complete a cognitive assessment reviewed by a neuropsychologist*	In-person in clinic<br>Online in clinic<br>Online at home<br>Any of the above<br>None of the above<br>Other (open text)
Patients with the following symptom(s) should be referred to neuropsychology or psychiatry for support or cognitive rehabilitation**	Low mood<br>Anxiety<br>Memory problems<br>Concentration problems<br>Taking longer to think<br>Restlessness<br>Irritable<br>Feeling frustrated<br>Fatigue<br>Sleep disturbance<br>Nausea<br>Dizziness (without an obvious underlying vestibular pathology)<br>Headache<br>Sensitivity to noise<br>Sensitivity to light<br>Visual symptoms (without an obvious underlying visual pathology)<br>None of the above<br>All the above<br>Case-by-case assessment
Patients with the following symptom(s) should be referred for physical therapy for physical rehabilitation**	Low mood<br>Anxiety<br>Memory problems<br>Concentration problems<br>Taking longer to think

For peer review only - http://bmjopen.bmj.com/site/about/guidelines.xhtml

	Restlessness<br>Irritable<br>Feeling frustrated<br>Fatigue<br>Sleep disturbance<br>Nausea<br>Dizziness (without an obvious underlying vestibular pathology)<br>Headache<br>Sensitivity to noise<br>Sensitivity to light<br>Visual symptoms (without an obvious underlying visual pathology)<br>None of the above<br>All the above<br>Case-by-case assessment
Patients with the following symptom(s) should be referred for cervical rehabilitation**	Low mood<br>Anxiety<br>Memory problems<br>Concentration problems<br>Taking longer to think<br>Restlessness<br>Irritable<br>Feeling frustrated<br>Fatigue<br>Sleep disturbance<br>Nausea<br>Dizziness (without an obvious underlying vestibular pathology)<br>Headache<br>Sensitivity to noise<br>Sensitivity to light

For peer review only

For peer review only - http://bmjopen.bmj.com/site/about/guidelines.xhtml

	Visual symptoms (without an obvious underlying visual pathology)<br>None of the above<br>All the above<br>Case-by-case assessment
Patients with the following symptom(s) should be referred for subsymptom threshold aerobic exercise**	Low mood<br>Anxiety<br>Memory problems<br>Concentration problems<br>Taking longer to think<br>Restlessness<br>Irritable<br>Feeling frustrated<br>Fatigue<br>Sleep disturbance<br>Nausea<br>Dizziness (without an obvious underlying vestibular pathology)<br>Headache<br>Sensitivity to noise<br>Sensitivity to light<br>Visual symptoms (without an obvious underlying visual pathology)<br>None of the above<br>All the above<br>Case-by-case assessment
Patients with the following symptom(s) should be referred for vestibular rehabilitation**	Low mood<br>Anxiety<br>Memory problems<br>Concentration problems<br>Taking longer to think

For peer review only - http://bmjopen.bmj.com/site/about/guidelines.xhtml
For peer review only

	Restlessness<br>Irritable<br>Feeling frustrated<br>Fatigue<br>Sleep disturbance<br>Nausea<br>Dizziness (without an obvious underlying vestibular pathology)<br>Headache<br>Sensitivity to noise<br>Sensitivity to light<br>Visual symptoms (without an obvious underlying visual pathology)<br>None of the above<br>All the above<br>Case-by-case assessment
Patients with the following symptom(s) should be referred for cervical rehabilitation**	Low mood<br>Anxiety<br>Memory problems<br>Concentration problems<br>Taking longer to think<br>Restlessness<br>Irritable<br>Feeling frustrated<br>Fatigue<br>Sleep disturbance<br>Nausea<br>Dizziness (without an obvious underlying vestibular pathology)<br>Headache<br>Sensitivity to noise<br>Sensitivity to light

For peer review only - http://bmjopen.bmj.com/site/about/guidelines.xhtml

For peer review only

	Visual symptoms (without an obvious underlying visual pathology)<br>None of the above<br>All the above<br>Case-by-case assessment
Patients with the following symptom(s) should be referred for hyperbaric oxygen therapy**	Low mood<br>Anxiety<br>Memory problems<br>Concentration problems<br>Taking longer to think<br>Restlessness<br>Irritable<br>Feeling frustrated<br>Fatigue<br>Sleep disturbance<br>Nausea<br>Dizziness (without an obvious underlying vestibular pathology)<br>Headache<br>Sensitivity to noise<br>Sensitivity to light<br>Visual symptoms (without an obvious underlying visual pathology)<br>None of the above<br>All the above<br>Case-by-case assessment
Patients with the following symptom(s) should be referred for transcranial magnetic stimulation.**	Low mood<br>Anxiety<br>Memory problems<br>Concentration problems<br>Taking longer to think

For peer review only - http://bmjopen.bmj.com/site/about/guidelines.xhtml

	Restlessness<br>Irritable<br>Feeling frustrated<br>Fatigue<br>Sleep disturbance<br>Nausea<br>Dizziness (without an obvious underlying vestibular pathology)<br>Headache<br>Sensitivity to noise<br>Sensitivity to light<br>Visual symptoms (without an obvious underlying visual pathology)<br>None of the above<br>All the above<br>Case-by-case assessment
There is not sufficient evidence for any pharmacological interventions to be routinely prescribed for persistent symptoms after mild TBI for the following symptom(s).**	Low mood<br>Anxiety<br>Memory problems<br>Concentration problems<br>Taking longer to think<br>Restlessness<br>Irritable<br>Feeling frustrated<br>Fatigue<br>Sleep disturbance<br>Nausea<br>Dizziness (without an obvious underlying vestibular pathology)<br>Headache<br>Sensitivity to noise<br>Sensitivity to light

For peer review only - http://bmjopen.bmj.com/site/about/guidelines.xhtml

	Visual symptoms (without an obvious underlying visual pathology)<br>None of the above<br>All the above<br>Case-by-case assessment

*Statement was amended and added as consensus statements in the second round of voting.

**Statements were added to get an overview of the panel's opinion regarding specific therapies for symptom profiles. The results resulted in the final statement denoted in supplementary material 2.1.

MDT, multidisciplinary team; MRI, Magnetic Resonance Imaging; TBI, Traumatic Brain Injury

Supplementary material 3: Consensus Statement Voting Round 2

**Statement included in voting round 2**	**Agreement**	**Outcome**
There should be a direct patient pathway from ED to specialist outpatient services available to all patients to ensure patients with persistent symptoms will be identified early.	81%	None
There is not sufficient evidence for injury-related events (i.e. presence of loss of consciousness, post-traumatic amnesia, vomiting) to predict prolonged symptoms.	80%	None
There is not sufficient evidence to support the clinical application of objective biomarkers (i.e. fluid, imaging) for identifying and screening for persistent symptoms after a mild TBI.	90%	None
There is not enough evidence to use prognostic tools to predict the risk of developing persistent symptoms.	75%	None
There should be a system for patients to be triaged to a specialist in (mild) TBI clinics based on persistent symptoms.	100%	None

For peer review only - http://bmjopen.bmj.com/site/about/guidelines.xhtml

Mild TBI patients with persistent symptoms should be seen in specialist clinics.	90%	None
Digital tools should be utilised to screen for persistent symptoms.	89%	None
Symptom scales are currently the most appropriate method available for measuring symptom burdens in patients.	74%	None
Symptom scales are currently the most appropriate method available to screen for persistent symptoms in patients.	74%	None
A bespoke symptom scale should be used for screening symptoms.	86%	None
All patients should complete a symptom scale at the first point of contact with a clinician in the acute phase.	70%	None
A bespoke patient-reported outcome measure (PROM) should be developed for mild TBI and used in routine clinical practice.	95%	None
There should be dedicated mild TBI clinics, or where not possible, TBI clinics with dedicated time to mild TBI patients.	95%	None
Specialist (mild) TBI clinics should comprise of a multidisciplinary team.	95%	None
All patients should be directed to a single bespoke online information portal on mild TBI to help with expectations and recommendations.	81%	None
Patients should be advised to rest (cognitive and physical rest) for 24-48 hours, after which they should gradually increase activity.	75%	None
There is not sufficient evidence to support the use of advanced imaging techniques in clinical practice.	81%	None

For peer review only - http://bmjopen.bmj.com/site/about/guidelines.xhtml

There is not sufficient evidence to support screening for clinical subtypes based on symptom clusters.	67%	Eliminated
All patients with persistent symptoms should complete a cognitive assessment reviewed by a neuropsychologist.	42%	Eliminated
All clinics should have access to standardised protocols for assessing pituitary dysfunction.	76%	None
Adjusting expectation and education should be the primary initial approach when managing mild TBI, regardless of symptoms.	90%	None
Co-morbidities should be factored into clinical decision-making on management.	100%	None
Socioeconomic status and social support should be considered during clinical decision-making on management.	86%	None
Where patients are involved in sports, they should be advised to return to work or studies without symptom exacerbation before returning to unsupervised physical exercise.	62%	Eliminated
Low-intensity physical activity should be recommended as long as it does not exacerbate symptoms.	95%	None
Treatment should be symptom-based and assessed on a case-by-case basis as there is currently not sufficient evidence to support a specific treatment approach for all mild TBI patients.	95%	None

ED, Emergency Department; TBI, Traumatic Brain Injury

For peer review only - http://bmjopen.bmj.com/site/about/guidelines.xhtml

Rationale for the choice of the Delphi technique	Report on page(s) in main document
1. *Justification.* The choice of the Delphi technique as a method of systematically collating expert consultation and building consensus needs to be well justified. When selecting the method to answer a particular research question, it is important to keep in mind its constructivist nature	1-2
Planning and design	
2. *Planning and process.* The Delphi technique is a flexible method and can be adjusted to the respective research aims and purposes. Any modifications should be justified by a rationale and be applied systematically and rigorously	2-4
3. *Definition of consensus.* Unless not reasonable due to the explorative nature of the study, an a priori criterion for consensus should be defined. This includes a clear and transparent guide for action on (a) how to proceed with certain items or topics in the next survey round, (b) the required threshold to terminate the Delphi process and (c) procedures to be followed when consensus is (not) reached after one or more iterations	3-4
Study conduct	
4. *Informational input.* All material provided to the expert panel at the outset of the project and throughout the Delphi process should be carefully reviewed and piloted in advance in order to examine the effect on experts' judgements and to prevent bias	3
5. *Prevention of bias.* Researchers need to take measures to avoid directly or indirectly influencing the experts' judgements. If one or more members of the research team have a conflict of interest, entrusting an independent researcher with the main coordination of the Delphi study is advisable	2
6. *Interpretation and processing of results.* Consensus does not necessarily imply the 'correct' answer or judgement; (non)consensus and stable disagreement provide informative insights and highlight differences in perspectives concerning the topic in question	3-4
Reporting	
7. *Purpose and rationale.* The purpose of the study should be clearly defined and demonstrate the appropriateness of the use of the Delphi technique as a method to achieve the research aim. A rationale for the choice of the Delphi technique as the most suitable method needs to be provided	1-2
8. *Expert panel.* Criteria for the selection of experts and transparent information on recruitment of the expert panel, socio-demographic details including information on expertise regarding the topic in question, (non)response and response rates over the ongoing iterations should be reported	2
9. *Description of the methods.* The methods employed need to be comprehensible; this includes information on preparatory steps (How was available evidence on the topic in question synthesised?), piloting of material and survey instruments, design of the survey instrument(s), the number and design of survey rounds, methods of data analysis,	2-4

processing and synthesis of experts’ responses to inform the subsequent survey round and methodological decisions taken by the research team throughout the process	
10. *Procedure.* Flow chart to illustrate the stages of the Delphi process, including a preparatory phase, the actual ‘Delphi rounds’, interim steps of data processing and analysis, and concluding steps	2
11. *Definition and attainment of consensus.* It needs to be comprehensible to the reader how consensus was achieved throughout the process, including strategies to deal with non-consensus	3-4
12. *Results.* Reporting of results for each round separately is highly advisable in order to make the evolving of consensus over the rounds transparent. This includes figures showing the average group response, changes between rounds, as well as any modifications of the survey instrument such as deletion, addition or modification of survey items based on previous rounds	4-8
13. *Discussion of limitations.* Reporting should include a critical reflection of potential limitations and their impact of the resulting guidance	12-13
14. *Adequacy of conclusions.* The conclusions should adequately reflect the outcomes of the Delphi study with a view to the scope and applicability of the resulting practice guidance	13
15. *Publication and dissemination.* The resulting guidance should be clearly identifiable from the publication, including recommendations for transfer into practice and implementation. If the publication does not allow for a detailed presentation of either the resulting practice guidance or the methodological features of the applied Delphi technique, or both, reference to a more detailed presentation elsewhere should be made (e.g. availability of the full guideline from the authors or online; publication of a separate paper reporting on methodological details and particularities of the process (e.g. persistent disagreement and controversy on certain issues)). A dissemination plan should include endorsement of the guidance by professional associations and health care authorities to facilitate implementation	8-12

only
